# N‐Substituted Nipecotic Acids as (*S*)‐SNAP‐5114 Analogues with Modified Lipophilic Domains

**DOI:** 10.1002/cmdc.201900719

**Published:** 2020-04-07

**Authors:** Michael C. Böck, Georg Höfner, Klaus T. Wanner

**Affiliations:** ^1^ Department of Pharmacy – Center for Drug Research Ludwig-Maximilians-Universität München Butenandtstraße 5–13 81377 Munich Germany

**Keywords:** GABA uptake inhibitors, mGAT4, medicinal chemistry, neurochemistry, structure-activity relationships

## Abstract

Potential mGAT4 inhibitors derived from the lead substance (*S*)‐SNAP‐5114 have been synthesized and characterized for their inhibitory potency. Variations from the parent compound included the substitution of one of its aromatic 4‐methoxy and 4‐methoxyphenyl groups, respectively, with a more polar moiety, including a carboxylic acid, alcohol, nitrile, carboxamide, sulfonamide, aldehyde or ketone function, or amino acid partial structures. Furthermore, it was investigated how the substitution of more than one of the aromatic 4‐methoxy groups affects the potency and selectivity of the resulting compounds. Among the synthesized test substances (*S*)‐1‐{2‐[(4‐formylphenyl)bis(4‐methoxyphenyl)‐methoxy]ethyl}piperidine‐3‐carboxylic acid, that features a carbaldehyde function in place of one of the aromatic 4‐methoxy moieties of (*S*)‐SNAP‐5114, was found to have a pIC_50_ value of 5.89±0.07, hence constituting a slightly more potent mGAT4 inhibitor than the parent substance while showing comparable subtype selectivity.

## Graphical abstract

**Searching for novel GABA uptake inhibitors**: We report the synthesis and biological evaluation of a series of N‐substituted nipecotic acids as potential inhibitors of the GABA transporter mGAT4. Derived from the lead substance (*S*)‐SNAP‐5114, the compounds feature polar moieties in the lipophilic domain in order to potentially enable increased polar interactions with the target.

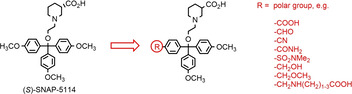

## Introduction

The neuronal signal transduction in the mammalian central nervous system (CNS) is regulated by a complex equilibrium of various excitatory and inhibitory neurotransmitters. γ‐Aminobutyric acid (GABA) is the most abundant of the latter,^1^ with approximately 40 % of synapses estimated to be GABAergic.^2^ Pathologically deficient GABA release into the synaptic cleft results in attenuation of the inhibitory component, which is associated with a variety of severe neurological disorders including epilepsy,^3^, ^4^ neuropathic pain,^5^ anxiety disorders,^6^ depression,^6^, ^7^ and Alzheimer's disease.^8^ A promising approach to the treatment of these disorders is the application of drugs that inhibit the GABA reuptake^8^, ^9^, ^10^, ^11^ into the presynaptic neurons and the surrounding glial cells, respectively, thereby enhancing the GABA concentration in the synaptic cleft and thus prolonging the effect of the released GABA. The specific and high affinity membrane based^12^ transport proteins accomplishing the GABA reuptake (GATs) are thus an important therapeutic target. Belonging to the solute carrier 6 (SLC6) family,^13^ they consist of 12 transmembrane helices, four of which (TM 1, TM3, TM6 and TM8) form the inner ring that in its center halfway across the cell membrane holds the central substrate binding site S1. Separated from the S1 binding site by the extracellular gate a second substrate binding site exists at the bottom of the extracellular vestibule, termed S2,^12^ the occupation of which is assumed to trigger the conformational changes necessary for the release of the substrate from the S1 pocket into the cell.^14^ Four GAT subtypes have been identified, termed GAT1, GAT2, GAT3, and BGT1 in accordance with the nomenclature proposed by the Gene Nomenclature Committee of the Human Genome Organisation (HUGO),^15^ or, when cloned from mouse brain, mGAT1 (≙GAT1), mGAT2 (≙BGT1), mGAT3 (≙GAT2) and mGAT4 (≙GAT3).^16^, ^17^ Since the biological test system developed in our group is based on GABA transporters cloned from mouse cells, in this paper the corresponding nomenclature will be used.

The predominant GABA transporter in the mammalian CNS, mGAT1, is located primarily in pre‐synaptic neuronal membranes,^18^ with its highest densities found in neocortex, spinal cord, brainstem, cerebellum, basal ganglia, and hippocampus.^2^, ^19^ As the occurrence of mGAT2 and mGAT3 in the CNS is restricted to low densities in specific brain structures, these subtypes play only a marginal role in the termination of the cerebral GABAergic neurotransmission.^20^, ^21^ mGAT4 is the second most abundant GABA transporter after mGAT1 and found particularly in olfactory bulb, brainstem, and diencephalon,^22^ where it is most commonly expressed on glia cells.^18^ The selective targeting of mGAT4 may therefore provide the possibility of treating neurological disorders associated with these brain regions with minimal impairment of the GABA reuptake in other parts of the CNS. Compared to mGAT1‐selective inhibitors such as Tiagabine (Gabatril®) (**1**, Table 1, entry 1), adverse effects, including dizziness, somnolence, headache, memory loss, and tremor,^23^ could hence be possibly reduced, resulting in more tolerable medication and better patient compliance.

**Table 1:** Binding affinities (p*K*_i_) and inhibitory potencies (pIC_50_) of reference compounds **1**–**5** from the literature.

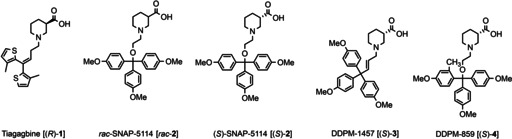						
Entry	Compound	p*K*_i_^[a]^	pIC_50_^[b]^			
		mGAT1	mGAT1	mGAT2	mGAT3	mGAT4
1	Tiagabine [(*R*)‐**1**]^[d]^	7.43±0.11	6.88±0.12	50 %	64 %	73 %
2	*rac*‐SNAP‐5114 [*rac*‐**2**] ^[c]^	–	4.08	–	4.96	5.64±0.05
3	(*S*)‐SNAP‐5114 [(*S*)‐**2**] ^[c]^	4.56±0.02	4.07±0.09	56 %	5.29±0.04	5.71±0.07
4	DDPM‐1457 [(*S*)‐**3**] ^[d]^	4.33±0.06	4.40±0.05	4.42±0.11	5.47±0.02	5.87±0.08
5	DDPM‐859 [(*S*)‐**4**] ^[d]^	–	4.19±0.07	4.12±0.08	4.85±0.04	5.78±0.03

[a] Results of the MS Binding Assays are given as p*K*_i_±SEM. [b] Results of the [^3^H]GABA uptake assays are given as pIC_50_±SEM. Percent values represent remaining [^3^H]GABA uptake in presence of 100 μM compound. [c] Reference literature ^[25]^. [d] Reference literature ^[26]^.

(*S*)‐SNAP‐5114 (*S*)‐**2** (Table 1, entry 3) can be considered the benchmark mGAT4 inhibitor. Since its publication,^24^ it has been the prototype for the development of further mGAT4 inhibitors with the goal of increasing potency, subtype selectivity, and chemical stability. The structural modifications implemented in the (*S*)‐SNAP‐5114 scaffold so far include variations of the spacer between the nipecotic acid partial structure and the trityl rest, as it is for example the case with DDPM‐1457 **3** (Table 1, entry 4),^25^ resulting in compounds with enhanced stability due to the labile trityl ether function being avoided. Variations of the substitution pattern of the trityl structure, e. g. by introduction of an additional methyl group in the 2‐position of one of the three aryl residues (**4**, Table 1, entry 5), was found to lead to compounds with slightly improved subtype selectivity. Unfortunately, the moderate potency inherent to the parent compound (*S*)‐**2**, which is characterized by a pIC_50_ value of 5.71±0.07 (Table 1, entry 3), remains largely unaffected by these structural modifications. This underlines the necessity of further research in order to advance the general understanding of the structure activity relationship (SAR) of mGAT4 inhibitors.

**Scheme 1:**
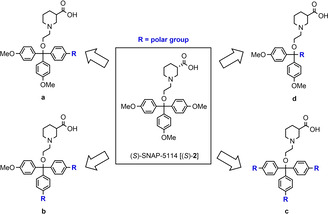
Overview of the structural modifications of (S)‐SNAP‐5114 conducted in this study.

To this end, the present study aims at the synthesis of (*S*)‐SNAP‐5114 [(*S*)‐**2**] analogues that feature a more polar moiety in place of one of the methoxy groups present in the trityl rest of the parent compound (*S*)‐**2** (Scheme 1, a). As such polar moieties carboxylic acid, alcohol aldehyde, nitrile, carboxamide, sulfonamide, aldehyde or ketone functions were taken into consideration, as well as amino acid partial structures. These modifications might result in increased polar interactions of the inhibitor with the target, thus possibly affecting its potency and subtype selectivity. In that context, we also aimed to clarify how a wider variation of the original (*S*)‐SNAP‐5114 [(*S*)‐**2**] structure, comprising the replacement of one entire 4‐methoxyphenyl moiety of the trityl residue by a polar group (Scheme 1, d), would influence the biological activity. A further question addressed with this study is how the inhibitory potency of (*S*)‐SNAP‐5114 [(*S*)‐**2**] analogues that have two (Scheme 1, b) or all three (Scheme 1, c) 4‐methoxy groups in the trityl moiety of (*S*)‐**2** substituted with other residues compare to inhibitors with only one such alteration. The findings would allow to draw conclusions about the SAR of mGAT4 inhibitors with regard to polarity, size, and number of matching substituents in the aromatic domain, and therefore point out important aspects to consider in future development of more potent mGAT4‐inhibitors.

## Results and Discussion

### Chemistry

**Scheme 2:**
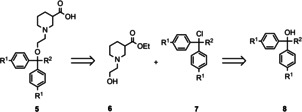
Retrosynthetic analysis for the preparation of (S)‐SNAP‐5114 analogues

Synthesis of the desired (*S*)‐SNAP‐5114 [(*S*)‐**2**] analogues was performed according to the reaction sequence shown in Scheme 2 that had been described by Schirrmacher et al.^27^ for the preparation of [^18^F] labelled (*S*)‐SNAP‐5114 analogues. According to this plan, the respective tertiary alcohols **8** exhibiting the desired polar function attached to the aryl moieties, or suitable precursors thereof, are transformed into the corresponding trityl chlorides, which can e. g. be accomplished by reaction with acetyl chloride. Subsequent treatment of the prepared trityl chlorides **7** with N‐(2‐hydroxyethyl)nipecotinate **6**, which may be prepared according to literature,^28^ will furnish the fully assembled target compounds in form of their carboxylic acid esters and finally the free nipecotic acids **5** upon hydrolysis of the carboxylic acid ester function. Although the stereochemistry of the nipecotic acid partial structure is known to play a decisive role in the biological activity of mGAT4 inhibitors,^24^ we opted for the synthesis of racemic compounds for economic reasons. However, for those racemic compounds showing equal or higher activity than (*S*)‐SNAP‐5114 in the biological testing, additionally the (*R*)‐ and (*S*)‐isomers should be synthesized and evaluated for their biological activity. To achieve the synthesis of these enantiopure compounds following the depicted synthetic pathway (Scheme 2), only *rac*‐**6** has to be replaced by its (*R*)‐ and (*S*)‐isomer, respectively.

### Synthesis of the tertiary alcohols 8 a–k

Initially for the construction of the target compounds **5 a**–**i** in which one or more of the three methoxy substituents of (*S*)‐SNAP‐5114 [(*S*)‐**2**] are replaced by an alternative polar moiety (compound type a‐c in Scheme 1), the corresponding tertiary alcohols **8 a**‐**i** had to be synthesized (Table 2, entry 1–9, Table 3, entry 9). This was accomplished by transforming aryl halides **9 a**–**h** into the corresponding Grignard or organolithium reagents, which were subsequently reacted with the appropriate electrophiles, i. e. with 4,4′‐dimethoxybenzophenone (**10 a**, Table 2, entry 1–6), 4‐cyanobenzoyl chloride (**10 b**, Table 2, entry 7), methyl 4‐methoxybenzoate (**10 c**, Table 2, entry 8), and dimethyl carbonate (**10 d**, Table 2, entry 9). This led to the differently substituted trityl alcohols **8 a**–**i** in acceptable to good yields of 53 %–99 %, most of which exhibit two of the three 4‐methoxyphenyl units present in (*S*)‐SNAP‐5114 [(*S*)‐**2**] together with a third aryl moiety with a different structure (Table 2, entry 1–7). Trityl alcohol **8 j** (Table 3, entry 9), featuring an amide group and two methoxy groups, respectively, in the 4‐position of the three aromatic moieties, was obtained in a yield of 96 % by hydration of the nitrile function of **8 g** (Table 2, entry 7), which was accomplished by the treatment with potassium *tert*‐butoxide in *tert*‐butanol, following a general procedure from the literature.^29^ 4,4′‐Dimethoxybenzilic acid methyl ester **8 k** (Table 3, entry 10) as precursor for the preparation of compounds of type d (Scheme 1) was synthesized by benzylic acid rearrangement of 4,4’‐dimethoxybenzil,^30^ followed by esterification with methyl iodide in analogy to a method published for benzylic acid.^31^

**Table 2:** Synthesis of the trityl alcohols **2 a**–**i**.

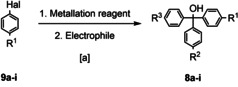										
Entry	Aryl halide		Metal‐ation reagent	Electrophile		Product	R^1^	R^2^	R^3^	Yield [%]
1	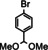	**9 a**	*t*‐BuLi	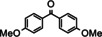	**10 a**	**8 a**	−CH(OMe)_2_	−OMe	−OMe	99
2	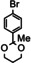	**9 b**	*t*‐BuLi	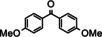	**10 a**	**8 b**	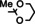	−OMe	−OMe	87
3	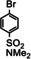	**9 c^[b]^**	*t*‐BuLi	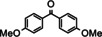	**10 a**	**8 c**	−SO_2_NMe_2_	−OMe	−OMe	53
4	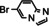	**9 d**	*n‐*BuLi	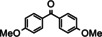	**10 a**	**8 d**	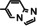	−OMe	−OMe	56
5	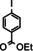	**9 e**	*i‐*PrMgCl	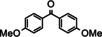	**10 a**	**8 e**	−COOEt	−OMe	−OMe	77
6	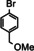	**9 f**	Mg	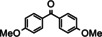	**10 a**	**8 f**	−CH_2_OMe	−OMe	−OMe	92
7	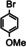	**9 g**	Mg	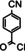	**10 b**	**8 g**	−CN	−OMe	−OMe	94
8	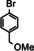	**9 f**	Mg	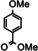	**10 c**	**8 h**	−CH_2_OMe	−CH_2_OMe	−OMe	70
9	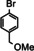	**9 f**	Mg	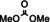	**10 d**	**8 i**	−CH_2_OMe	−CH_2_OMe	−CH_2_OMe	85

Reagents and conditions: [a] entry 1–3: *t*‐BuLi (2.0 eq), THF, −78 °C, 2 h, 4,4′‐dimethoxybenzophenone (1.0 eq), THF, −78 °C‐rt; entry 5: *i*‐PrMgCl (1.0 eq), THF, −20 °C, 1.5 h, 4,4′‐dimethoxybenzophenone (1.0 eq), THF, −20 °C‐rt; entry 6: n‐BuLi (1.0 eq), diethyl ether, −78 °C, 0.5 h, 4,4′‐dimethoxybenzophenone (0.83 eq), diethyl ether, −78 °C‐rt; entry 7: magnesium (1.0 eq), THF, rt, 4‐cyanobenzoylchloride, THF, 0 °C‐rt; entry 8: magnesium (1.0 eq), THF, rt, methyl 4‐methoxybenzoate (0.89 eq), THF, reflux; entry 9: magnesium (1.0 eq), THF, rt, dimethyl carbonate (0.33 eq), THF, reflux. [b] Synthesized by N‐alkylation of 4‐bromobenzenesulfonamide with dimethyl sulfate (2.0 eq) in presence of potassium carbonate (4.0 eq) and tetrabutylammonium tetrafluoroborate (10 mol%) under reflux conditions.

**Table 3:** Synthesis of the N‐substituted nipecotic acid ethyl esters **12 a**–**k** and their hydrolysis to the free nipecotic acid derivatives **5 b**–**k**.

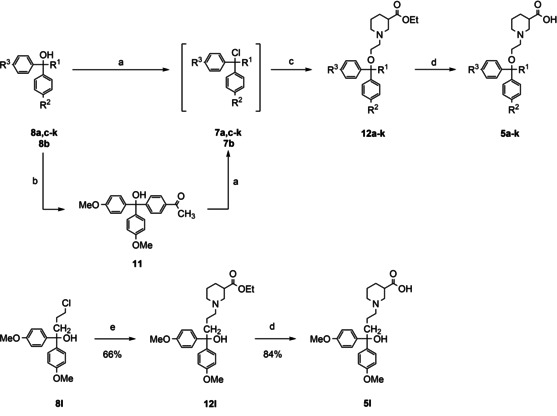										
Entry	Starting material	R^1^	R^2^	R^3^	Product of step a+b	R^1^	Yield [%]^[f]^	Product of step d	R^1^	Yield [%]
1	**8 a**	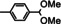	−OMe	−OMe	**12 a**	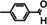	70	**5 a**	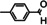	89
2	**8 b**	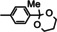	−OMe	−OMe	**12 b**	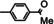	50	**5 b**	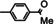	86
3	**8 c**	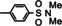	−OMe	−OMe	**12 c**	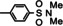	64	**5 c**	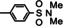	88
4	**8 d**	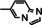	−OMe	−OMe	**12 d**	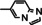	48	**5 d**	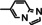	80
5	**8 e**	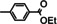	−OMe	−OMe	**12 e**	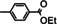	54	**5 e**	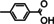	61
6	**8 f**	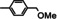	−OMe	−OMe	**12 f**	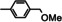	44	**5 f**	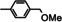	81
7	**8 g**	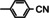	−OMe	−OMe	**12 g**	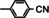	85	**5 g**	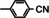	86
8	**8 h**	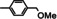	−CH_2_OMe	−OMe	**12 h**	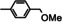	46	**5 h**	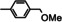	94
9	**8 i**	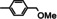	−OMe	−CH_2_OMe	**12 i**	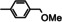	42	**5 i**	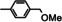	90
10	**8 j**	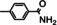	−OMe	−OMe	**12 j**	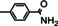	49	**5 j**	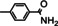	87
11	**8 k**	−COOMe	−OMe	−OMe	**12 k**	−COOMe	88	**5 k**	−COOH	97

Reagents and conditions: [a] acetyl chloride, dimethyl formamide (cat.), rt; [b] HCl, THF/H_2_O, reflux; [c] 1‐(2‐hydroxyethyl)nipecotic acid ethyl ester (**6**) (1.1 eq), potassium carbonate (2.5 eq), acetonitrile, rt; [d] barium hydroxide octahydrate (2–4 eq), methanol/water 4 : 1; carbon dioxide; [e] ethyl nipecotinate (1.1 eq), potassium carbonate (2.5 eq), potassium iodide (0.1 eq), acetonitrile, microwave, 80 °C. [f] Yield over two steps.

### Construction of target compounds 5 a–l from N‐(2‐hydroxyethyl)nipecotic acid ethyl ester 6 and tertiary alcohols 8 a–l

The synthesis of the target compounds **5 a**–**k** should be achieved by etherification of the hydroxy function of N‐(2‐hydroxyethyl)nipecotic acid ethyl ester **6** with the tertiary alcohols **8 a**–**k** that had been prepared for this purpose. To this end, in the first step alcohols **8 a**–**k** were transformed into the corresponding tertiary chlorides **7 a**–**k** by reaction with acetyl chloride in the presence of a catalytic amount of dimethyl formamide (Table 3, step a). Under these conditions, also the acetal function of **8 a** was completely transformed in an aldehyde group as wanted. Therefore, the reaction mixture obtained after treatment of **8 a** with acetyl chloride, resulting in **7 a** with the deprotected aldehyde function, could be directly used for the next step, the etherification reaction with N‐(2‐hydroxyethyl)nipecotic acid ethyl ester (**6**). In contrast, the more stable acetal function of **8 b** was only partially transformed into the ketone under the conditions for the halide formation. Hence, **8 b** was first transformed into ketone **11** by refluxing in aqueous acid (93 % yield), which was subsequently converted in chloride **7 b**. Due to their high reactivity and susceptibility to hydrolysis upon exposure to moisture, the tertiary chlorides were directly used without prior purification or characterization for the next step, the alcoholysis with racemic 1‐(2‐hydroxyethyl)nipecotic acid ethyl ester (**6**) (Table 3, step b). The desired products **12 a**–**k** with the newly created trityl ether function were thus obtained in yields from 42 % to 88 % over both reaction steps (based on **8 a**–**k** as starting material).

For the synthesis of an analogue of *rac*‐SNAP‐5114 [(*S*)‐**2**] that features a hydroxyl function in place of one of the 4‐methoxyphenyl groups, we decided to use a propyl instead of the ethoxy linker to warrant chemical stability of the product. The synthesis was accomplished by reacting anisyl lithium with ethyl 4‐chlorobutanoate to give the required alcohol **8 l** with an γ‐chloropropyl residue, which upon reaction with ethyl nipecotinate led to the desired N‐substituted nipecotic acid ester **12 l** in 66 % yield (Table 3, step c).

In order to obtain the free nipecotic acid derivatives **5 a**–**l**, compounds **12 a**–**l** were treated with barium hydroxide octahydrate in methanol/water 4 : 1 for the hydrolysis of the ester function, followed by workup with carbon dioxide (Table 3, step d). Thereupon, the target compounds **5 a**–**l** could be isolated in yields from 61 % to 97 %.

### Synthesis of target compounds 5 m–t with amino acid derived residues substituting one of the three methoxy groups in (*S*)‐SNAP‐5114 [(*S*)‐2]

Next, we aimed at the synthesis *rac*‐SNAP‐5114 analogues that feature a hydroxy methyl moiety or an amino acid subunit as a polar group replacing one of the three methoxy residues in the lipophilic domain of (*S*)‐SNAP‐5114 [(*S*)‐**2**]. As starting material for the synthesis compound **12 a**, a nipecotic acid derivative with one of the three methoxy groups of the trityl moiety having been replaced by a formyl residue was used. Reduction of the aldehyde function in **12 a** was accomplished by treatment with sodium borohydride in methanol, providing the corresponding alcohol **12 m** in a yield of 89 % (Table 4, entry 1). Reductive amination of the aldehyde function in **12 a** was performed employing a set of amino acids and amino acid ester hydrochlorides (Table 4, entry 2–8). Upon application of a slightly modified standard method^32^ employing sodium acetoxyborohydride in dichloromethane as reductant (instead of dichloroethane) to **12 a** and the respective amino acid ester hydrochlorides led to the glycine methyl ester **12 n**, β‐alanine ethyl ester **12 o**, 1‐aminocyclopropane‐1‐carboxylic acid ethyl ester **12 p**, and 2‐amino‐2‐methylpropanoic acid ethyl ester derivatives **12 q** (Table 4, entry 2–5) in yields of 42 to 64 %. The γ‐aminobutyric acid ethyl ester derivative of **12 a** could not be obtained by this procedure since the product underwent partial γ‐lactamization during workup, resulting in a mixture of the desired compound and the corresponding γ‐butyrolactam. Attempts to reopen the lactam function under basic conditions (barium hydroxide or sodium hydroxide at rt or reflux) (Table 3, entry 6) led to a completion of the formation of lactam **5 r**, or to cleavage of the ether function when lithium hydroxide was used under reflux conditions. Hence, the reductive amination of **12 a** was attempted with the free γ‐aminobutyric acid (GABA). Applying the established reaction conditions did not lead to the desired product **12 s**, which is likely to be due to the very low solubility of GABA in dichloromethane. However, when dichloromethane was replaced by methanol and sodium triacetoxyborohydride by sodium cyanoborohydride, **12 s** (Table 4, entry 7) was formed in a yield of 40 %. The same method could also be successfully applied to the synthesis of p‐aminobenzoic acid derivative **12 t** (Table 4, entry 8). The free nipecotic acids **5 m**–**q** and **5 s**–**t** became finally available upon subjecting compounds **12 m**–**q** and **12 s**–**t** to alkaline hydrolysis using the procedure as already described above [Ba(OH)_2_ ⋅ 8 H_2_O, CO_2_ workup] for the transformation of compounds **12 a**–**l** into **5 a**–**l** (Table 4, step b), the yields of this transformation amounting to 65–96 %.

**Table 4:** syntesis of the target compounds **5 m**–**t** with amino acid derived residues substituting one of the three methoxy groups in (S)‐SNAP‐5114.

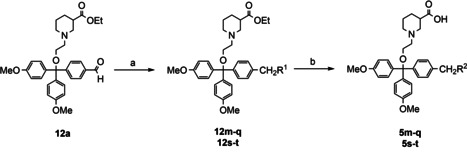						
Entry	Product of step a	R^1^	Yield [%]	Product of step b	R^2^	Yield [%]
1	**12 m**	−OH	89	**5 m**	−OH	69
2	**12 n**	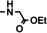	57	**5 n**	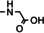	65
3	**12 o**	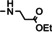	42	**5 o**	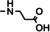	74
4	**12 p**	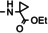	59	**5 p**	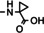	82
5	**12 q**	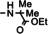	64	**5 q**	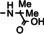	92
6				**5 r**	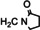	47^[c]^
7	**12 s**	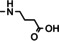	40	**5 s**	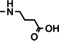	84
8	**12 t**	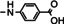	25	**5 t**	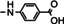	96

Reagents and conditions: [a] entry 1: sodium borohydride (2.5 eq), methanol, rt; entry 2–5: sodium triacetoxyborohydride (1.4 eq), amino acid ethyl ester hydrochloride (2.0 eq), dichloromethane, rt; entry 7–8: sodium cyanoborohydride (1.4 eq), free amino acid (2.0 eq), methanol, rt. [b] barium hydroxide octahydrate (2–4 eq), methanol/water 4 : 1; carbon dioxide. [c] Yield over two steps.

## Biological evaluation

The N‐substituted nipecotic acids **5 a**–**t**, and their ester precursors **12 a**–**q** and **12 s**–**t** were tested for their inhibitory potencies on the four GABA transporter subtypes mGAT1‐4 in a [^3^H]GABA uptake assay that was previously developed by our group.^33^ The tests were performed in a standardized manner using HEK293 cell lines, each expressing one of the four tested GABA transporter subtypes. Furthermore, binding affinities towards mGAT1 were examined employing a standardized MS Binding Assay with NO711 as native MS marker.^34^ For compounds that in preliminary experiments did not reduce [^3^H]GABA uptake beyond 50 % at a test concentration of 100 μM, which equals a pIC_50_ of≤4.0, only the percent values of the remaining [^3^H]GABA uptake are listed. Correspondingly, the percentage of remaining marker is given in cases when the tested compound did not cause a reduction of the MS marker binding beyond 50 %, equating to a p*K*_i_ of≤4.0. When [^3^H]GABA uptake or NO711 binding was reduced below 50 %, at a concentration of 100 μM, inhibitory potencies (pIC_50_ values) and binding affinities (p*K*_i_ values) were determined in full scale [^3^H]GABA uptake and MS Binding Assays, respectively, measurements being performed as triplicates. For compounds with pIC_50_ ([^3^H]GABA uptake assay) or p*K*_i_ (MS Binding Assays) values close to or above 5.0, these experiments were repeated twice and SEM have been calculated. The results are summarized in Table 5.

**Table 5:** Binding affinities (p*K*_i_) and inhibitory potencies (pIC_50_) of the N‐substituted nipecotic acids **6 a**, **c**–**u** and nipecotic acid ethyl esters **12 a**, **c**–**r**, **t**–**u**.

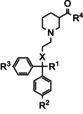											
Entry	Compound	R^1^	R^2^	R^3^	R^4^	X	p*K*_i_^[a]^	pIC_50_^[b]^			
								mGAT1	mGAT2	mGAT3	mGAT4
1	**12 a**	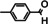	OMe	OMe	OEt	O	85.4 %	55.5 %	4.57	4.90	55.9 %
2	**5 a**		OMe	OMe	OH	O	4.41±0.11	4.17±0.08	66.3 %	4.98±0.08	5.77±0.04
3	**(*S*)**‐**5 a**		OMe	OMe	OH	O	77.4 %	4.14±0.00	72.6 %	5.16±0.05	5.89±0.07
4	**(*R*)**‐**5 a**		OMe	Ome	OH	O	4.91±0.09	4.45±0.08	50.1 %	4.47±0.05	4.86±0.03
5	**12 b**	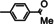	OMe	OMe	OEt	O	84.6 %	72.9 %	66.4 %	55.2 %	84.2 %
6	**5 b**		OMe	OMe	OH	O	4.51±0.03	64.6 %	64.2 %	4.24±0.02	5.43±0.04
7	**12 c**	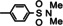	OMe	OMe	OEt	O	93.8 %	75.7 %	73.2 %	95.9 %	94.3 %
8	**5 c**		OMe	OMe	OH	O	62.1 %	67.9 %	78.0 %	77.1 %	82.0 %
9	**12 d**	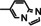	OMe	OMe	OEt	O	77.2 %	4.39	4.88	4.71	4.24
10	**5 d**		OMe	OMe	OH	O	4.03	84.9 %	72.9 %	81.8 %	4.58
11	**12 e**	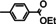	OMe	OMe	OEt	O	74.8 %	84.6 %	87.3 %	79.0 %	76.1 %
12	**5 e**	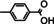	OMe	OMe	OH	O	71.4 %	96.5 %	85.2 %	4.24	4.86
13	**12 f**	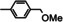	OMe	OMe	OEt	O	55.3 %	66.6 %	58.8 %	56.9 %	50.8 %
14	**5 f**		OMe	OMe	OH	O	89.4 %	67.1 %	72.7 %	4.51±0.08	5.42±0.10
15	**12 g**	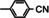	OMe	OMe	OEt	O	97.6 %	76.6 %	80.7 %	63.6 %	71.9 %
16	**5 g**		OMe	OMe	OH	O	4.40±0.10	74.8 %	82.9 %	4.31±0.12	5.07±0.12
17	**12 h**	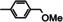	CH_2_OMe	OMe	OEt	O	72.9 %	64.9 %	4.30	50.6 %	4.05
18	**5 h**		CH_2_OMe	OMe	OH	O	74.1 %	70.5 %	55.2 %	52.2 %	4.77
19	**12 i**		CH_2_OMe	CH_2_OMe	OEt	O	87.5 %	70.3 %	65.0 %	4.02	51.4 %
20	**5 i**		CH_2_OMe	CH_2_OMe	OH	O	92.7 %	90.7 %	72.6 %	68.3 %	76.1 %
21	**12 j**	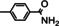	OMe	OMe	OEt	O	89.3 %	57.8 %	56.1 %	4.10	60.0 %
22	**5 j**		OMe	OMe	OH	O	87.0 %	79.6 %	96.1 %	82.1 %	65.2 %
23	**12 k**	−COOMe	OMe	OMe	OEt	O	90.8 %	89.1 %	92.6 %	64.1 %	78.2 %
24	**5 k**	−COOH	OMe	OMe	OH	O	96.9 %	100 %	101 %	94.0 %	96.5 %
25	**12 l**	−OH	OMe	OMe	OEt	CH _2_	104.0 %	79.3 %	70.4 %	65.0 %	73.8 %
26	**5 l**	−OH	OMe	OMe	OH	CH _2_	4.49	69.5 %	62.5 %	80.7 %	92.0 %
27	**12 m**	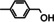	OMe	OMe	OEt	O	75.3 %	50.4 %	49.3 %	4.45	4.23
28	**5 m**		OMe	OMe	OH	O	55.0 %	78.1 %	91.6 %	58.7 %	4.90
29	**12 n**	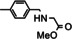	OMe	OMe	OEt	O	91.7 %	51.8 %	4.29	4.47	4.18
30	**5 n**	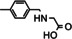	OMe	OMe	OH	O	86.2 %	81.8 %	96.1 %	90.9 %	72.2 %
31	**12 o**	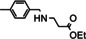	OMe	OMe	OEt	O	72.4 %	4.59	4.72	4.63	4.66
32	**5 o**	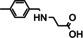	OMe	OMe	OH	O	82.3 %	94.4 %	69.5 %	89.1 %	56.3 %
33	**12 p**	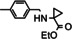	OMe	OMe	OEt	O	95.8 %	75.5 %	103 %	58.8 %	66.1 %
34	**5 p**	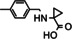	OMe	OMe	OH	O	58.8 %	78.2 %	76.0 %	69.1 %	4.88
35	**12 q**	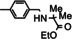	OMe	OMe	OEt	O	90.9 %	66.9 %	67.4 %	49.3 %	57.4 %
36	**5 q**	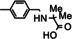	OMe	OMe	OH	O	77.0 %	80.7 %	77.0 %	61.1 %	4.34
37	**5 r**	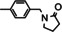	OMe	OMe	OH	O	97.0 %	76.0 %	79.7 %	64.2 %	4.19
38	**12 s**	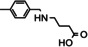	OMe	OMe	OEt	O	84.9 %	52.4 %	76.7 %	59.3 %	67.6 %
39	**5 s**		OMe	OMe	OH	O	86.5 %	48.5 %	51.5 %	4.33	4.13
40	**12 t**	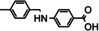	OMe	OMe	OEt	O	101.6 %	52.3 %	73.5 %	49.7 %	63.9 %
41	**5 t**		OMe	OMe	OH	O	54.1 %	99,5 %	4,18	96,0 %	50,0 %

[a] Results of the MS Binding Assays are given as p*K*_i_±SEM. For compounds with low p*K*_i_ values only one measurement was performed, therefore no SEM can be reported. Percent values represent remaining specific NO711 binding in presence of 100 μM compound. [b] Results of the [^3^H]GABA uptake assays are given as pIC_50_±SEM. For compounds with low pIC_50_ values only one measurement was performed, therefore no SEM can be reported. Percent values represent remaining [^3^H]GABA uptake in presence of 100 μM compound.

As outlined above, the structure of the prototypic mGAT4 inhibitor *rac*‐SNAP‐5114 (*rac*‐**2**) was modified by formally replacing one of the three aromatic methoxy moieties in its lipophilic domain with a variety of different functional groups. For the purpose of estimating the effect that these modifications exert on the polarity of the resulting molecule, the log *D* values of the compounds under physiological conditions (pH=7.4, electrolyte concentration=0.154 mmol/l) were calculated (clog *D*: calculated log *D*) using MarvinSketch.^35^

Replacement of one of the methoxy substituents in the *rac*‐SNAP‐5114 (*rac*‐**2**) molecule by the larger methoxymethylene group leads to **5 f**, which at mGAT4 exhibits a pIC_50_ value of 5.42±0.10 (Table 5, entry 14). This constitutes only a minor decrease in inhibitory potency at the transporter compared to the parent compound *rac*‐**2** (pIC_50_=5.64±0.05, Table 1, entry 2), suggesting that a slight increase in the space requirements of the substituent is tolerated relatively well by mGAT4. If a nitrile function is introduced in place of a methoxy moiety, the loss of potency at mGAT4 is more pronounced, amounting to a pIC_50_ value 5.07±0.12 (**5 g**, Table 5, entry 16). Since the nitrile function is also a hydrogen bridge acceptor and conveys very similar polarity compared to the methoxy moiety it replaces (clog *D* of **5 g** 2.33, clog D of *rac*‐**2** 2.32), this finding is likely attributable to the linear shape of the nitrile function not being ideal for interaction with the target. For *rac*‐**5 a**, featuring an aldehyde function in one of the aromatic 4‐positions of *rac*‐SNAP‐5114 (*rac*‐**2**), a pIC_50_ of 5.77±0.04 was determined (Table 5, entry 2), which places the potency of compound *rac*‐**5 a** nominally above that of *rac*‐SNAP‐5114 (*rac*‐**2**) despite the oxygen atom being placed one bond further away from the aromatic moiety. **5 b**, which has one of the methoxy groups of *rac*‐**2** replaced by the larger, but similar polar acetyl moiety (**5 b**: clog *D*=2.00), exhibits only a slightly decreased inhibitory potency compared to the parent compound, with its pIC_50_ value amounting to 5.43±0.04 (Table 5, entry 6). On the other hand, the introduction of a hydroxymethylene group, leading to the distinctly more polar compound **5 m** (clog *D*=1.71), is accompanied by a reduction of the pIC_50_ value from 5.64±0.05 for *rac*‐SNAP‐5114 to 4.90, which equates to a potency loss of _∼_0.75 log units (Table 5, entry 28). Interestingly, an equivalent pIC_50_ of 4.86 was determined for **5 e** (Table 5, entry 12), which has one of the three aromatic 4‐positions of *rac*‐**2** occupied by a carboxylic acid moiety. This finding is astonishing as the carboxyl group is significantly more polar than the corresponding alcohol moiety present in **5 m**, with the respective clog *D* value amounting to −1.40 under physiological conditions.

By contrast, the inhibitory potency of **5 j**, featuring a carboxamide moiety in this position, is strongly reduced (pIC_50_<4.0, remaining [^3^H]GABA at 100 μM=65.2 %, Table 5, entry 22), irrespective of the fact that this group is of similar size as the carboxylic acid found in **5 e** while conveying a less pronounced increase in polarity (clog *D*=1.32). Apparently, no correlation between polarity and inhibitory potency seems to exist. **5 c**, which features a N,N‐dimethyl sulfonamide group (clog *D*=1.98) in place of one of the methoxy groups of *rac*‐**2**, is characterized by an even lower activity; the compound causes a [^3^H]GABA uptake reduction to 82.0 % at 100 μM, denoting a pIC_50_ of well below 4.0 (Table 5, entry 8).

Of the compounds obtained from **12 a** by reductive amination and subsequent hydrolysis, **5 n**, featuring a glycine partial structure that is connected to the 4‐position of one of the aromatic moieties by a methylene spacer, reduces the [^3^H]GABA uptake to 72.2 % at 100 μM test compound concentration (Table 5, entry 30); its analogue derived from β‐alanine, **5 o**, causes a slightly more pronounced reduction to 56.3 % (Table 5, entry 32). In this context, it is noticeable that the potency of the free nipecotic acid derivatives **5 n** and **5 o** is even lower than that of the corresponding esters **12 n** [pIC_50_ (mGAT4)=4.18, Table 5, entry 29] and **12 o** [pIC_50_ (mGAT4)=4.66, Table 5, entry 31]. By contrast, compounds that contain bulky, sterically hindered amino acid partial structures in the same position, such as 1‐aminocyclopropane‐1‐carboxylic acid (**5 p**) and 2‐amino‐2‐methylpropanoic acid (**5 q**), are characterized by a pIC_50_ of 4.88 (Table 5, entry 34) and 4.34 (Table 5, entry 36), respectively. Since these substances retain comparatively more of the inhibitory activity at mGAT4 of the parent compound, increased space requirements do evidently not contribute to the decline in potency in this case. Compound **5 s**, which formally results from elongation of the amino acid chain of **5 o** by one methylene group, hence exhibiting a γ‐aminobutyric acid subunit, shows slightly higher inhibitory potency with a pIC_50_ value of 4.13 compared to its shorter chain analogues (compare Table 5, entry 39 to entry 30 and 32). Compound **5 t**, whose 4‐aminobenzoic acid partial structure resembles to some extent a more rigid homologue of the γ‐aminobutyric acid moiety present in **5 s**, is characterized by equal inhibitory potency (remaining [^3^H]GABA at 100 μM=50.0 %, Table 5, entry 41). Surprisingly, **5 r**, featuring a lactam structure derived from the γ‐aminobutyric acid substructures present in **5 s**, exhibits a similar activity at mGAT4 (pIC_50_=4.19, Table 5, entry 37), despite not possessing an ionized functional group in the aromatic domain and thus having a distinctly higher clog *D* value of 1.87 compared to the value of −0.44 calculated for **5 s**.

In conclusion, the data imply no clear correlation between the polarity and size of the compound and the inhibitory potency exerted at mGAT4. Still the fact remains conspicuous that all test compounds with a free carboxylic acid function of the nipecotic acid residue possessing a pIC_50_≥5.0 contain a functional group that can act as hydrogen bond acceptor, but not as donor, and that is of similar size as the 4‐methoxy moiety in the lipophilic domain it replaces. Furthermore, the clog *D* values calculated for these compounds consistently lie in the range of 2.0–2.4, as is the case with the parent compound *rac*‐**2** (clog *D*=2.32) and the most potent inhibitor synthesized within the scope of this study, **5 a** (clog *D*=2.15). This might turn out to be beneficial with regard to potential therapeutic applications, since compounds with log *D* values between 2 and 5,^36^ or even better with values closer to 2,^37^ are thought to possess the highest propensity for blood‐brain barrier (BBB) penetration.

Since **5 a** turned out to be the most potent mGAT4 inhibitor of all racemic compounds tested for this study, its enantiopure isomers (*S*)‐**5 a** and (*R*)‐**5 a** were synthesized and evaluated for their biological activities as well. As is the case with the lead substance SNAP‐5114, the (*S*)‐enantiomer of **5 a** was found to exhibit a more pronounced inhibitory potency at mGAT4 than the (*R*)‐enantiomer, with the respective pIC_50_ values amounting to 5.89±0.07 for (*S*)‐**5 a** (Table 5, entry 3) and 4.86±0.03 for (*R*)‐**5 a** (Table 5, entry 4). Hence, (*S*)‐**5 a** constitutes nominally a more potent mGAT4 inhibitor than the benchmark compound (*S*)‐**2** (pIC_50_=5.71±0.07, Table 1, entry 3).

Next, we aimed to elucidate whether the biological activity of **5 a** is solely attributable to competitive inhibition at the target, or whether a reaction of the aldehyde function with [^3^H]GABA serving as substrate in the [^3^H]GABA uptake assay might have affected the outcome of the biological study. Reaction of [^3^H]GABA with the aldehyde function of the test compounds **5 a**, (*S*)‐**5 a** and (*R*)‐**5 a** might lead to depletion of the substrate of the uptake assay, thus falsifying the outcome of the assay. Alternatively, a thus formed intermediate might be a potent inhibitor by itself. To this end the uptake experiment was repeated using an assay recently developed by our group,^38^ which has GABA replaced by the chemically inert imidazolacetic acid. Since **5 a** and its enantiopure isomers (*R*)‐**5 a** and (*S*)‐**5 a** display similar inhibitory activity when tested under these conditions as compared to the assay based on [^3^H]GABA, it appears reasonable to assume that possible reactions of the aldehyde function of **5 a** with the substrate does not, or at least not crucially, contribute to the biological effects observed for this compound.

Due to the presence of an aldehyde function in **5 a**, it had to be considered whether the compound **5 a** might form covalent bonds with the target. In order to explore this possibility, we examined **5 a** in comparison with (*S*)‐SNAP‐5114 [(*S*)‐**2**] for inhibition of GABA uptake at mGAT4 by varying the time period used for preincubation (i. e. the period in which target and test compound are in contact, before the substrate is added). We studied preincubation times of 10, 25 min (which is our standard preincubation time in our GABA uptake assays) as well as 60 min and compared the inhibitory potencies of **5 a** and (*S*)‐SNAP‐5114 [(*S*)‐**2**] under these conditions. The determined pIC_50_‐values revealed a very slight enhancement of inhibitory potencies with increasing preincubation times almost exactly to the same extent for both compounds (see supporting information, Table 1). As **5 a** and (*S*)‐SNAP‐5114 [(*S*)‐**2**] behaved identical in this respect, the basic way of interaction of both compounds with mGAT4 seems to be similar, arguing against a covalent interaction of **5 a** with mGAT4.

Of the *rac*‐SNAP‐5114 (*rac*‐**2**) analogues that have an entire 4‐methoxyphenyl residue replaced by a polar moiety, **5 d**, possessing an imidazo[1,2‐α]pyridine subunit instead of the aforementioned 4‐methoxyphenyl rest, displays a pIC_50_ of 4.58 (Table 5, entry 10). As compared to the potency of *rac*‐**2 a** at mGAT4 (pIC_50_=5.64±0.05) this equates to a distinct, but still moderate reduction of inhibitory potency of about one log unit. However, the introduction of a small, polar functional group in place of one of the 4‐methoxyphenyl residues is associated with a drastic loss of activity at mGAT4. For example, the compound featuring a carboxylic acid function in this position, **5 k**, is devoid of almost any inhibitory activity at mGAT4, reducing the [^3^H]GABA uptake to only 96.5 % at 100 μM (Table 5, entry 24). By comparison, **5 e**, which also features a carboxyl group, albeit located on one of the aromatic residues of the lipophilic domain of *rac*‐**2** supplanting a 4‐methoxy group, conserves significantly more of the inhibitory potency of the parent compound [pIC_50_(mGAT4)=4.86, Table 5, entry 12]. The same phenomenon is observed for **5 l**, that formally results from the replacement of one of the 4‐methoxyphenyl groups of *rac*‐**2** with a hydroxy moiety, in combination with a simultaneous exchange of the ether oxygen of the spacer by a methylene group to warrant chemical stability. The compound reduces the [^3^H]GABA uptake to 92.0 % at 100 μM (Table 5, entry 26). Compound **5 m** on the other hand, which also contains a hydroxy function, but retains triaryl pattern of the parent compound by substituting one of the three methoxy groups with a hydroxymethylene moiety, still possesses a pIC_50_ of 4.90 (Table 5, entry 28). These results suggest that decreasing size and steric demand of the lipophilic domain is accompanied by a pronounced decline in biological activity at mGAT4. The three aromatic moieties on the quaternary carbon atom can therefore be regarded as essential for high inhibitory potency at mGAT4 of these compounds, and variations, e. g. in order to alter the polarity of a compound, should only be implemented by modification of the substituents, but not by replacement of one of these aromatic moieties in favour of a smaller functional group.

Next, we aimed to study how the number of aryl moieties in the lipophilic domain, which are modified with regard to their substituents, affects the inhibitory potency. To that end, an array of *rac*‐SNAP‐5514 analogues was synthesized that had one, two, or all three of the methoxy groups replaced by methoxymethylene moieties. While the introduction of one methoxymethylene group caused only a minor decrease in inhibitory potency to a pIC_50_ of 5.42 (**5 f**, Table 5, entry 14) as compared to the reference compound *rac*‐**2** (pIC_50_=5.64±0.05, Table 1, entry 2), the loss of activity became increasingly more pronounced with each further methoxymethylene group (**5 h**, mGAT4: pIC_50_=4.77, Table 5, entry 18; **5 i**, mGAT4: remaining [^3^H]GABA at 100 μM=76.1 %, Table 5, entry 20). These findings support the notion that the biological system can tolerate an inapt substituent on one of the aryl groups relatively well as long as the other two 4‐methoxy moieties of the lipophilic domain remain unchanged and thus are still available for interactions with the target. However, substitution of further methoxy moieties will result in an exceeding decline of inhibitory potency at mGAT4.

Also the nipecotic acid ester derivatives **12** that have been synthesized in this study were evaluated for their inhibitory potencies at all four GAT subtypes. With regard to mGAT4 inhibition, most nipecotic acid esters **12** display distinctly lower potencies at this transporter subtype than their nipecotic acid analogues **5**, signifying the importance of the free nipecotic acid partial structure for mGAT4 inhibition. Only in case of the nipecotic acids **5 j**–**5 l** which exert very low inhibitory potencies at mGAT4 (pIC_50_<4.0), the inhibitory potencies of the free acids were lower than that of the corresponding esters **12 j**–**12 l**, the differences being, however, marginal. However, carboxylic acid ester **12 k** cannot be directly compared to nipecotic acid **5 k**, as the latter possesses a carboxy function instead of a methoxy carbonyl moiety attached to one of the aryl residues of the lipophilic domain.

Regarding the effects at other GAT subtypes, inhibition of mGAT1 is the pharmacologically most relevant due to its high abundance in the mammalian CNS. Of the compounds presented in this study, none of the carboxylic acid analogues of *rac*‐SNAP‐5114 **5** show a reasonable inhibitory potency at mGAT1, i. e. pIC_50_ values≥4, which is also true for the ester analogues **12** with two exceptions. Thus, for **12 d**, that has one of the three 4‐methoxyphenyl groups (of *rac*‐**2**) substituted by an imidazo[1,2‐a]pyridine‐7‐yl moiety, and **12 o**, featuring a β‐alanine ethyl ester partial structure which is linked to one of the three aromatic residues in the 4‐position by a methylene spacer, pIC_50_ values of 4.39 and 4.59 were found (**12 d**, Table 5, entry 9, and **12 o**, Table 5, entry 31). All other compounds lie below that mark, i. e. a pIC_50_=4.0, and can thus be regarded as less potent mGAT1 inhibitors than *rac*‐**2**, which has a pIC_50_ of 4.08 (**2**, Table 1, entry 2). Hence, contrary to what applies to the potency at mGAT4, transforming the ester function of **12** into a free acid moiety exerts little to no influence on inhibitory potency at mGAT1, the latter usually amounting to pIC_50_ values of below 4.0 for both compound classes.

The compounds synthesized and tested for this study were generally found to be weak binders at mGAT1, which correlates well with the uniformly low inhibitory potencies at this GABA transporter subtype. In that context, only the *rac*‐SNAP‐5114 analogues possessing a formyl [(**5 a**), (*R*)‐**5 a**], acetyl (**5 b**), or nitrile function (**5 g**) in the lipophilic domain or in which a 4‐methoxyphenyl residue has been replaced by an imidazo[1,2‐a]pyridine‐7‐yl moiety (**5 d**) or an hydroxy function (**5 l**) differ by displaying moderate p*K*_i_ values >4.0. In detail, the values determined for these compounds were 4.50±0.06 (**5 a**, Table 5, entry 2), 4.91±0.09 [(*R*)‐**5 a**, Table 5, entry 4], 4.51±0.03 (**5 b**, Table 5, entry 6), 4.40±0.01 (**5 g**, Table 5, entry 16), 4.03 (**5 d**, Table 5, entry 10) and 4.49 (**5 l**, Table 5, entry 26), respectively.

Furthermore, none of the free nipecotic acids **5** reduced [^3^H]GABA below 50 % at a concentration of 100 μM when tested at mGAT2, which corresponds to pIC_50_ values <4.0, except for **5 t** (pIC_50_=4.18, Table 5, entry 41). Nevertheless, some of the ester precursors **12** exceeded this mark, the most potent among these being the imidazo[1,2‐a]pyridine derivative **12 d** with a pIC_50_ of 4.88 (Table 5, entry 9). Likewise, the ester derivatives of the compounds that feature in the lipophilic domain a formyl (**12 a**, Table 5, entry 1, pIC_50_=4.57), a methoxymethyl (**12 h**, Table 5, entry 17, pIC_50_=4.30) or an amino acid residue (**12 n**, Table 5, entry 29, pIC_50_=4.29; **12 o**, Table 5, entry 31, pIC_50_=4.72) display pIC_50_ values≥4.00 at this transporter subtype.

Serving as lead structure of this study, *rac*‐SNAP‐5114 (*rac*‐**2**) possesses a comparatively high pIC_50_ value at mGAT3 of 4.96 (Table 1, entry 2); as a consequence, its desired mGAT4 selectivity is quite poor with regard to this GAT subtype. The modifications of the *rac*‐**2** structure that were undertaken for the purpose of this study resulted in compounds with lower activity at mGAT3, **5 a** being the only exception (pIC_50_=4.98±0.08, Table 5, entry 2). This is of particular importance for those compounds that show relatively high inhibitory potency at mGAT4 and are thus of interest as pharmacological tools. In case of **5 b** and **5 f**, which comprise an acetyl group and a methoxymethylene group in the lipophilic domain, the inhibitory potency at mGAT4 is moderately reduced to pIC_50_ values of 5.43±0.04 (**5 b**, Table 5, entry 6) and 5.42±0.10 (**5 f**, Table 5, entry 14), respectively. However, the potency loss at mGAT3 caused by these structural modifications is slightly more pronounced, with a pIC_50_ value of 4.24±0.02 determined for **5 b** (Table 5, entry 6) and 4.51±0.04 for **5 f** (Table 5, entry 14). Accordingly, these compounds display considerably higher mGAT4 selectivity than *rac*‐**2**, albeit at the cost of some inhibitory potency.

As compared to the enantiopure benchmark inhibitor (*S*)‐**2** (Table 1, entry 3), the most potent compound synthesized for this study, (*S*)‐**5 a**, displays almost identical subtype selectivity (Table 5, entry 3). In particular, the pIC_50_ values amount to 5.16±0.05 at mGAT3 [(*S*)‐**2**: 5.29±0.04] and 4.14±0.00 at mGAT1 [(*S*)‐**2**: 4.07±0.09]. At mGAT2 both compounds exert only weak inhibitory effects, reducing the [^3^H]GABA uptake to 72.6 % [(*S*)‐**5 a**] and 56 % [(*S*)‐**2**], respectively. Hence, (*S*)‐**5 a** can be considered a viable alternative to (*S*)‐**2** as a pharmacological tool.

## Conclusions

In order to gain insight into the structure activity relationship of mGAT4 inhibitors, analogues of *rac*‐SNAP‐5114 were synthesized that differ from the parent compound by having one or more of the 4‐methoxy groups attached to the lipophilic domain, or a complete 4‐methoxyphenyl group, replaced by a moiety with higher polarity. These modifications might increase interactions between the inhibitor and polar regions of binding site, and hence improve binding affinity, inhibitory potency, and selectivity.

The test compounds were accessible through conversion of tertiary alcohols featuring the respective functional groups or appropriate precursors into the corresponding chlorides, followed by etherification with ethyl N‐(2‐hydroxyethyl)nipecotinate. The obtained N‐substituted nipecotic acid esters were either directly hydrolyzed at this stage, or functional group interconversions were performed beforehand, including the introduction of various amino acid partial structures via reductive amination.

The biological evaluation of the obtained N‐substituted nipecotic acids revealed no direct correlation between the polarity of the newly introduced group and the biological activity of the resulting compound. However, compounds with clog *D* values of 2.0–2.4 consistently exert the highest inhibitory potencies. In this context, we found (*S*)‐1‐{2‐[(4‐formylphenyl)bis(4‐methoxyphenyl)methoxy]‐ethyl}piperidine‐3‐carboxylic acid [(*S*)‐**5 a**], that has one of the three methoxy groups of the parent substance substituted by a formyl moiety, to be the most potent test compound synthesized in this study. Being characterized by a pIC_50_ value of 5.89±0.07 at mGAT4, it even exhibits slightly higher inhibitory potency than the benchmark substance (*S*)‐SNAP‐5114, while showing comparable subtype selectivity. Furthermore, the racemic SNAP‐5114 analogues comprising in the lipophilic domain an acetyl and an methoxymethylene substituent, respectively, were found to be slightly less potent mGAT4 inhibitors than the parent compound, but somewhat more subtype selective, especially at mGAT3.

Also, it has been demonstrated that *rac*‐SNAP‐5114 analogues that have one of the aromatic moieties in the lipophilic domain replaced by a small, non‐aromatic moiety show a distinct deprivation of inhibitory potency. This finding indicates the essentiality of the triaryl structure in the lipophilic domain for the biological activity of mGAT4 inhibitors. Finally, we determined how the substitution of several methoxy groups in the lipophilic domain of *rac*‐SNAP‐5114 impacts the inhibitory potency of the resulting compounds using the example of the methoxymethylene group. As the results of the biological evaluation suggest, mGAT4 can tolerate one inapt substituent reasonably well. However, the replacement of additional methoxy groups will result in an exceeding decline of inhibitory potency.

These findings are expected to be beneficial for future developments of more potent mGAT4 inhibitors since they reveal essential structure activity relationships with regard to size, polarity, and electronic effects of the substituents in the lipophilic domain.

## Experimental Section

### Chemistry

Moisture‐sensitive reactions were carried out in oven‐dried glassware under inert gas atmosphere. Commercially available starting materials were used without further purification. Dry acetonitrile (MeCN) was purchased from VWR (HiPerSolv Chromanonorm, water content >30 ppm) and tetrahydrofuran (THF) was freshly distilled from sodium benzophenone ketyl. All other solvents were distilled prior to use. Microwave reactions were carried out with CEM Discover® SP Microwave Synthesizer (model no. 909 155). Flash column chromatography was performed according to Still et al.^39^ using Merck silica gel 60 (mesh 0.040–0.063 mm) as stationary phase. Thin‐layer chromatography (TLC) was carried out on Merck silica gel 60 F_254_ sheets. ^1^H and ^13^C NMR spectra were, unless stated otherwise, recorded at room temperature with J NMR‐GX (JEOL 400 or 500 MHz) or Bruker BioSpin Avance III HD (400 or 500 MHz) and integrated with the NMR software MestReNova. IR samples were measured as KBr pellets or film with Perkin‐Elmer FT‐IR 1600. HRMS data were obtained with JMS‐GCmate II (EI, Jeol) or Thermo Finnigan LTQ FT Ultra (ESI, Thermo Finnigan).

*General procedure for the synthesis of trityl alcohols by Grignard reaction* (**GP1**): Magnesium turnings were scraped with a glass rod and suspended in dry THF. The appropriate aryl halide or a solution thereof in THF was added portion wise. After completion of the Grignard reagent formation a solution of the electrophile in THF was added dropwise to the solution. The mixture was stirred for the prescribed time and temperature, quenched with saturated ammonium chloride (NH_4_Cl) solution, diluted with water and extracted thrice with dichloromethane (CH_2_Cl_2_). The combined organic phases were dried over magnesium sulfate (MgSO_4_), filtered and reduced in vacuum.

*General procedure for the synthesis of trityl alcohols by lithiation of an aryl halide and subsequent reaction with 4,4′‐dimethoxybenzophenone* (**GP2**): *tert*‐Butyllithium solution (1.7 M in pentane, 2.0 eq.) was added dropwise to a solution of the aryl halide (1.0 eq.) in THF at −78 °C. After 2 h a solution of 4,4′‐dimethoxybenzophenone (1.0 eq) in THF was added. The mixture was allowed to slowly warm up to room temperature overnight, quenched with water and extracted thrice with CH_2_Cl_2_. The combined organic phases were dried over MgSO_4_, filtered and reduced in vacuum.

*General procedure for the etherification of tertiary alcohols* (**GP3**): The tertiary alcohol (1.0 eq) was charged with a catalytic amount of DMF. Acetyl chloride was added and the reaction mixture was stirred at room temperature for 24 hours, reduced in vacuum and dried under high vacuum. The oily or solid residue was solved in dry MeCN. Racemic, (*R*)‐ or (*S*)‐ethyl 1‐(hydroxyalkyl)nipecotinate (1.1 eq) and oven‐dried potassium carbonate (K_2_CO_3_, 2.5 eq) were added. After stirring 16 hours at room temperature, the mixture was filtered and reduced in vacuum.

*General procedure for the hydrolysis of the ethyl ester function* (**GP4**): The ester (1.0 eq) was dissolved in MeOH. Double distilled water and barium hydroxide octahydrate (2.0–4.0 eq) were added and the mixture was stirred at room temperature until TLC indicated complete consumption of the ester. Carbon dioxide was passed through the solution until no further precipitate formed. The suspension was diluted with MeOH (1 : 1), filtered through a paper filter and reduced in vacuum. If necessary, the crude acid was purified by flash column chromatography. The solid residue was solved in MeOH (1.0 mL), filtered through a syringe filter (Perfect‐Flow®, WICOM Germany GmbH, PTFE, 0.2 μM), diluted with double distilled water (4.0 mL) and lyophilized.

*General procedure for reductive amination I* (**GP5**): The appropriate amino acid ester hydrochloride (2.0 eq) and sodium triacetoxyborohydride (1.4 eq) were added to a solution of aldehyde **3 a** (1.0 eq) in CH_2_Cl_2_. The reaction mixture was stirred at room temperature until TLC indicated complete consumption of the aldehyde, quenched with saturated sodium carbonate (Na_2_CO_3_) solution and extracted thrice with CH_2_Cl_2_. The combined organic phases were dried over MgSO_4_, filtered and reduced in vacuum.

*General procedure for reductive amination II* (**GP6**): The appropriate amino acid (2.0 eq) and sodium cyanoborohydride (1.4 eq) were added to a solution of aldehyde **3 a** (1.0 eq) in MeOH. The reaction mixture was stirred at room temperature until TLC indicated complete consumption of the aldehyde and reduced in vacuum.

***rac‐*** **1‐{2‐[(4‐Formylphenyl)bis(4‐methoxyphenyl)methoxy]ethyl}piper‐idine‐3‐carboxylic acid (5 a)**: GP4 was followed using **12 a** (101 mg, 0.190 mmol), barium hydroxide octahydrate (241 mg, 0.764 mmol), MeOH/H_2_O 4 : 1 (5.0 mL). The crude compound was purified by flash column chromatography on silica (eluent MeOH). Amorphous colourless solid (85 mg, 89 %).

**(*S*)‐1‐{2‐[(4‐Formylphenyl)bis(4‐methoxyphenyl)methoxy]ethyl}piper‐idine‐3‐carboxylic acid [(*S*)‐5 a]**: GP4 was followed using (*S*)‐**12 a** (38 mg, 0.071 mmol), barium hydroxide octahydrate (90 mg, 0.28 mmol), MeOH/H_2_O 4 : 1 (5.0 mL). The crude compound was purified by flash column chromatography on silica (eluent MeOH). Amorphous colourless solid (34 mg, 93 %).

**(*R*‐1‐{2‐[(4‐Formylphenyl)bis(4‐methoxyphenyl)methoxy]ethyl}piperidine‐3‐carboxylic acid [(*R*)‐5 a]**: GP4 was followed using (*R*)‐**12 a** (37 mg, 0.070 mmol), barium hydroxide octahydrate (89 mg, 0.28 mmol), MeOH/H_2_O 4 : 1 (5.0 mL). The crude compound was purified by flash column chromatography on silica (eluent MeOH). Amorphous colourless solid (32.5 mg, 92 %).

***rac*** **‐1‐{2‐[(4‐Acetylphenyl)bis(4‐methoxyphenyl)methoxy]ethyl}piperidine‐3‐carboxylic acid (5 b)**: GP4 was followed using **12 b** (87 mg, 0.16 mmol), barium hydroxide octahydrate (197 mg, 0.62 mmol), MeOH/H_2_O 4 : 1 (3.5 mL). The crude compound was purified by flash column chromatography on silica (eluent MeOH). Amorphous colourless solid (69 mg, 86 %).

***rac*** **‐1‐(2‐{[4‐(N,N‐dimethylsulfamoyl)phenyl]bis[4‐methoxyphenyl]‐methoxy}ethyl)piperidine‐3‐carboxylic acid (5 c)**: GP4 was followed using **12 c** (66 mg, 0.11 mmol), barium hydroxide octahydrate (137 mg, 0.434 mmol), MeOH/H_2_O 4 : 1 (3.0 mL). The crude compound was purified by flash column chromatography on silica (eluent MeOH). Amorphous colourless solid (55 mg, 88 %).

***rac‐*** **1‐(2‐{Imidazo[1,2‐a]pyridin‐6‐ylbis(4‐methoxyphenyl)methoxy}‐ethyl)piperidine‐3‐carboxylic acid (5 d)**: GP4 was followed using **12 d** (78 mg, 0.14 mmol), barium hydroxide octahydrate (170 mg, 0.54 mmol), MeOH/H_2_O 4 : 1 (5.0 mL). The crude compound was purified by flash column chromatography on silica (eluent MeOH/CH_2_Cl_2_ 1 : 1). Amorphous colourless solid (59 mg, 80 %).

***rac*** **‐1‐{2‐[(4‐Carboxyphenyl)bis(4‐methoxyphenyl)methoxy]ethyl}piper‐idine‐3‐carboxylic acid (5 e)**: GP4 was followed using **12 e** (200 mg, 0.350 mmol), barium hydroxide octahydrate (222 mg, 0.704 mmol), MeOH/H_2_O 4 : 1 (5.0 mL). The crude compound was purified by flash column chromatography on silica (eluent MeOH). Amorphous colourless solid (110 mg, 61 %).

***rac*** **‐1‐(2‐{[4‐(Methoxymethyl)phenyl]bis[4‐methoxyphenyl]methoxy}‐ethyl)piperidine‐3‐carboxylic acid (5 f)**: GP4 was followed using **12 f** (72 mg, 0.13 mmol), barium hydroxide octahydrate (83 mg, 0.26 mmol), MeOH/H_2_O 4 : 1 (12.0 mL). The crude compound was purified by flash column chromatography on silica (eluent MeOH). Amorphous colourless solid (56 mg, 81 %).

***rac*** **‐1‐{2‐[(4‐Cyanophenyl)bis(4‐methoxyphenyl)methoxy]ethyl}piperidine‐3‐carboxylic acid (5 g)**: GP4 was followed using **12 g** (48 mg, 0.090 mmol), barium hydroxide octahydrate (57 mg, 0.18 mmol), MeOH/H_2_O 4 : 1 (2.0 mL). The crude compound was purified by flash column chromatography on silica (eluent MeOH). Amorphous colourless solid (39 mg, 86 %).

***rac‐*** **1‐(2‐{Bis[4‐(methoxymethyl)phenyl][4‐methoxyphenyl]methoxy}‐ethyl)piperidine‐3‐carboxylic acid (5 h)**: GP4 was followed using **12 h** (56 mg, 0.10 mmol), barium hydroxide octahydrate (127 mg, 0.403 mmol), MeOH/H_2_O 4 : 1 (5.0 mL). The crude compound was purified by flash column chromatography on silica (eluent MeOH). Amorphous colourless solid (51 mg, 94 %).

***rac‐*** **1‐(2‐{Tris[4‐(methoxymethyl)phenyl]methoxy}ethyl)piperidine‐3‐carboxylic acid (5 i)**: GP4 was followed using **12 i** (61 mg, 0.11 mmol), barium hydroxide octahydrate (75 mg, 0.44 mmol), MeOH/H_2_O 4 : 1 (5.0 mL). The crude compound was purified by flash column chromatography on silica (eluent MeOH). Amorphous colourless solid (52 mg, 90 %).

***rac*** **‐1‐{2‐[(4‐Carbamoylphenyl)bis(4‐methoxyphenyl)methoxy]ethyl}‐piperidine‐3‐carboxylic acid (5 j)**: GP4 was followed using **12 j** (63 mg, 0.12 mmol), barium hydroxide octahydrate (146 mg, 0.46 mmol), MeOH/H_2_O 4 : 1 (5.0 mL). The crude compound was purified by flash column chromatography on silica (eluent MeOH). Amorphous colourless solid (52 mg, 87 %).

***rac*** **‐1‐{2‐[Carboxybis(4‐methoxyphenyl)methoxy]ethyl}piperidine‐3‐carboxylic acid (5 k)**: GP4 was followed using **12 k** (370 mg, 0.760 mmol), barium hydroxide octahydrate (481 mg, 1.52 mmol), MeOH/H_2_O 4 : 1 (5.0 mL). The crude compound was purified by flash column chromatography on silica (eluent MeOH). Amorphous colourless solid (327 mg, 97 %).

***rac*** **‐1‐[4‐Hydroxy‐4,4‐bis(4‐methoxyphenyl)butyl]piperidine‐3‐carboxylic acid (5 l)**: GP4 was followed using **12 l** (103 mg, 0.230 mmol), barium hydroxide octahydrate (148 mg, 0.469 mmol), MeOH/H_2_O 4 : 1 (3.6 mL). The crude compound was purified by flash column chromatography on silica (eluent MeOH). Amorphous colourless solid (81 mg, 84 %).

**rac‐1‐(2‐{[4‐(Hydroxymethyl)phenyl]bis[4‐methoxyphenyl]methoxy}‐ethyl)piperidine‐3‐carboxylic acid (5 m)**: GP4 was followed using **12 m** (58 mg, 0.11 mmol), barium hydroxide octahydrate (69 mg, 0.22 mmol), MeOH/H_2_O 4 : 1 (3.0 mL). The crude compound was purified by flash column chromatography on silica (eluent MeOH). Amorphous colourless solid (38 mg, 69 %).

***rac‐*** **1‐{2‐[(4‐{[(Carboxymethyl)amino]methyl}phenyl)bis(4‐methoxy‐phenyl)methoxy]ethyl}piperidine‐3‐carboxylic acid (5 n)**: GP4 was followed using **12 n** (35 mg, 0.060 mmol), barium hydroxide octahydrate (37 mg, 0.12 mmol), MeOH/H_2_O 4 : 1 (5.0 mL). The crude compound was purified by flash column chromatography on silica (eluent MeOH). Amorphous colourless solid (29 mg, 65 %).

***rac‐*** **1‐{2‐[(4‐{[(2‐Carboxyethyl)amino]methyl}phenyl)bis(4‐methoxy‐phenyl)methoxy]ethyl}piperidine‐3‐carboxylic acid (5 o)**: GP4 was followed using **12 o** (83 mg, 0.13 mmol), barium hydroxide octahydrate (171 mg, 0.542 mmol), MeOH/H_2_O 4 : 1 (5.0 mL). The crude compound was purified by flash column chromatography on silica (eluent MeOH). Amorphous colourless solid (57 mg, 74 %).

***rac‐*** **1‐{2‐[(4‐{[(1‐Carboxycyclopropyl)amino]methyl}phenyl)bis(4‐methoxyphenyl)methoxy]ethyl}piperidine‐3‐carboxylic acid (5 p)**: GP4 was followed using **12 p** (51 mg, 0.079 mmol), barium hydroxide octahydrate (100 mg, 0.317 mmol), MeOH/H_2_O 4 : 1 (6.0 mL). The crude compound was purified by flash column chromatography on silica (eluent MeOH). Amorphous colourless solid (50 mg, 82 %).

***rac*** **‐1‐{2‐[(4‐{[(2‐Carboxypropan‐2‐yl)amino]methyl}phenyl)bis(4‐methoxyphenyl)methoxy]ethyl}piperidine‐3‐carboxylic acid (5 q)**: GP4 was followed using **12 q** (35 mg, 0.054 mmol), barium hydroxide octahydrate (136 mg, 0.431 mmol), MeOH/H_2_O 4 : 1 (5.0 mL). The crude compound was purified by flash column chromatography on silica (eluent MeOH). Amorphous colourless solid (29.3 mg, 92 %).

***rac‐*** **1‐[2‐(Bis{4‐methoxyphenyl}{4‐[(2‐oxopyrrolidin‐1‐yl)methyl]phenyl}‐methoxy)ethyl]piperidine‐3‐carboxylic acid (5 r)**: GP5 was followed using **12 a** (173 mg, 0.330 mmol), ethyl 4‐aminobutanoate hydrochloride (109 mg, 0.650 mmol), sodium triacetoxyborohydride (155 mg, 0.730 mmol), CH_2_Cl_2_ (2.0 mL). The crude compound was purified by flash column chromatography on silica (eluent ethyl acetate+5 % triethylamine), yielding 107 mg of a mixture of *rac*‐ethyl 1‐[2‐(bis{4‐methoxyphenyl}{4‐[(2‐oxopyrrolidin‐1‐yl)methyl]phenyl}‐methoxy)ethyl]piperidine‐3‐carboxylate and *rac*‐ethyl 1‐{2‐[(4‐{[(4‐methoxy‐4‐oxobutyl)amino]methyl}phenyl)bis(4‐methoxyphenyl)methoxy]ethyl}piperidine‐3‐carboxylate. 84 mg thereof were subjected to GP4 using barium hydroxide octahydrate (178 mg, 0.564 mmol), MeOH/H_2_O 4 : 1 (3.5 mL). The crude compound was purified by flash column chromatography on silica (eluent MeOH). Amorphous colourless solid (69 mg, 47 % over both steps).

***rac*** **‐1‐{2‐[(4‐{[(3‐Carboxypropyl)amino]methyl}phenyl)bis(4‐methoxy‐phenyl)methoxy]ethyl}piperidine‐3‐carboxylic acid (5 s)**: GP4 was followed using **12 s** (50 mg, 0.081 mmol), barium hydroxide octahydrate (103 mg, 0.326 mmol), MeOH/H_2_O 4 : 1 (5.0 mL). Amorphous colourless solid (40 mg, 84 %).

***rac‐*** **1‐{2‐[(4‐{[(4‐Carboxyphenyl)amino]methyl}phenyl)bis(4‐methoxy‐phenyl)methoxy]ethyl}piperidine‐3‐carboxylic acid (5 t): 12 t** (37.2 mg, 0.057 mmol) was dissolved in THF (1.5 mL) and H_2_O (1.5 mL) and barium hydroxide octahydrate (38.4 mg, 4.0 eq) were added. The mixture was stirred at room temperature until TLC indicated complete consumption of **12 t**. Carbon dioxide was passed through the solution until no further precipitate formed. The suspension was filtered through a syringe filter (Perfect‐Flow®, WICOM Germany GmbH, PTFE, 0.2 μM) and lyophilized. Amorphous colourless solid (34.0 mg, 96 %).

**[4‐(Dimethoxymethyl)phenyl]bis(4‐methoxyphenyl)methanol (8 a)**: GP2 was followed using *tert*‐butyllithium solution (1.7 M in pentane, 1.2 mL, 2.04 mmol), 1‐bromo‐4‐(dimethoxymethyl)benzene **9 a** (235 mg, 1.02 mmol), THF (3.0 mL). Addition of 4,4′‐dimethoxybenzophenone **10 a** (245 mg, 1.00 mmol) in THF (5.0 mL). The crude compound was purified by flash column chromatography on silica (eluent pentane/Et_2_O 7 : 3). Colourless oil (393 mg, 99 %).

**Bis(4‐methoxyphenyl)[4‐(2‐methyl‐1,3‐dioxan‐2‐yl)phenyl]methanol (8 b)**: GP2 was followed using t*ert*‐butyllithium solution (1.7 M in pentane, 3.3 mL, 5.6 mmol), 2‐(4‐bromophenyl)‐2‐methyl‐1,3‐dioxane **9 b** (713 mg, 2.77 mmol), THF (12.0 mL). Addition of 4,4′‐dimethoxybenzophenone **10 a** (700 mg, 2.83 mmol) in THF (12.0 mL). The crude compound was purified by flash column chromatography on silica (eluent pentane/Et_2_O 8 : 2). Colourless semi‐solid (1.02 g, 87 %).

**4‐[Hydroxybis(4‐methoxyphenyl)methyl]‐N,N‐dimethylbenzenesulfon‐amide (8 c)**: GP2 was followed using *tert*‐butyllithium solution (1.7 M in pentane, 2.7 mL, 4.6 mmol), **9 c** (561 mg, 2.27 mmol), THF (30.0 mL). Addition of 4,4′‐ dimethoxybenzophenone **10 a** (561 mg, 2.27 mmol) in THF (12.0 mL). The crude compound was precipitated from isohexane/THF. Amorphous colourless solid (514 mg, 53 %).

**Imidazo[1,2‐a]pyridin‐6‐ylbis(4‐methoxyphenyl)methanol (8 d)**: *N*‐butyl‐lithium solution (2.4 M in hexane, 1.0 mL, 2.4 mmol) was added dropwise to a suspension of 6‐bromoimidazo[1,2‐a]pyridine **9 d** (483 mg, 2.40 mmol) in Et_2_O (24.0 mL) at −78 °C. After 30 min a suspension of 4,4′‐dimethoxybenzophenone **10 a** (494 mg, 2.0 mmol) in Et_2_O was added. The mixture was stirred 1 h at −78 °C, warmed to room temperature, quenched with water (20.0 mL) and extracted thrice with ethyl acetate (20.0 mL). The combined organic phases were washed with saturated Na_2_CO_3_ solution, dried over MgSO_4_, filtered and reduced in vacuum. The crude compound was purified by flash column chromatography on silica (eluent CH_2_Cl_2_+2 % MeOH). Amorphous slightly yellowish solid (400 mg, 56 %).

**Ethyl 4‐[hydroxybis(4‐methoxyphenyl)methyl]benzoate (8 e)**: Isopropyl‐magnesium chloride (2.0 M solution in THF, 1.0 mL, 2.0 mmol) was added dropwise to a solution of ethyl 4‐iodobenzoate **9 e** (547 mg, 2.02 mmol) in THF (5.0 mL) at −20 °C. The mixture was stirred for 1.5 h and a solution of 4,4′‐dimethoxybenzophenone **10 a** (445 mg, 1.80 mmol) in THF (5.0 mL) was slowly added via syringe. Stirring was continued at 0 °C over night. The reaction mixture was allowed to slowly warm up to room temperature, quenched with saturated NH_4_Cl solution and extracted thrice with CH_2_Cl_2_. The combined organic phases were dried over MgSO_4_, filtered and reduced in vacuum. The resulting crude compound was purified by flash column chromatography on silica (eluent pentane/Et_2_O 8 : 2). Colourless semi‐solid (328 mg, 77 %).

**[4‐(Methoxymethyl)phenyl]bis(4‐methoxyphenyl)methanol (8 f)**: GP2 was followed using t*ert*‐butyllithium solution (1.7 M in pentane, 5.0 mL, 8.50 mmol), 1‐bromo‐4‐(methoxymethyl)benzene **9 f** (848 mg, 4.22 mmol), THF (24.0 mL). Addition of 4,4′‐dimethoxybenzophenone **10 a** (1.05 g, 4.25 mmol) in THF (12.0 mL). The crude compound was purified by flash column chromatography on silica (eluent pentane/Et_2_O 7 : 3). Amorphous colourless solid (1.42 g, 92 %).

**4‐[Hydroxybis(4‐methoxyphenyl)methyl]benzonitrile (8 g)**: GP1 was followed using a Grignard reagent prepared from 4‐bromoanisol **9 g** (1.72 g, 9.01 mmol) and magnesium (219 mg, 9.01 mmol) in THF (20.0 mL). A solution of 4‐cyanobenzoyl chloride **10 c** (736 mg, 4.40 mmol) in THF (8.0 mL) was added dropwise at 0 °C. The reaction mixture was stirred 2 h at 0 °C and 30 min at 25 °C. The crude compound was purified by flash column chromatography on silica (eluent pentane/Et_2_O 6 : 4). Amorphous colourless solid (1.42 g, 94 %).

**Bis[4‐(methoxymethyl)phenyl](4‐methoxyphenyl)methanol (8 h)**: GP1 was followed using a Grignard reagent prepared from 1‐bromo‐4‐(methoxymethyl)benzene **9 f** (745 mg, 3.70 mmol) and magnesium (90 mg, 3.7 mmol) in THF (6.0 mL). A solution of methyl 4‐methoxybenzoate (560 mg, 3.30 mmol) in THF (6.0 mL) was added dropwise. The reaction mixture was stirred at room temperature for 16 h and refluxed 90 min. The crude compound was purified by flash column chromatography on silica (eluent pentane/Et_2_O 6 : 4). Pale yellowish oil (492 mg, 70 %).

**Tris[4‐(methoxymethyl)phenyl]methanol (8 i)**: GP1 was followed using a Grignard reagent prepared from 1‐bromo‐4‐(methoxymethyl)benzene **9 f** (1.41 g, 7.00 mmol) and magnesium (170 mg, 6.99 mmol) in THF (10.0 mL). Dimethyl carbonate (209 mg, 2.30 mmol) was added dropwise at room temperature. The reaction mixture was refluxed 90 min, then stirred at room temperature over night. The crude compound was purified by flash column chromatography on silica (eluent pentane/Et_2_O 1 : 1). Colourless oil (771 mg, 85 %).

**4‐[Hydroxybis(4‐methoxyphenyl)methyl]benzamide (8 j)**: A mixture of 4‐[hydroxybis(4‐methoxyphenyl)methyl]benzonitrile **8 g** (171 mg, 0.500 mmol) and potassium *tert*‐butoxide (340 mg, 3.00 mmol) was stirred in *tert*‐butanol (4.0 mL) at 50 °C for 40 h. Water (25 mL) was added ad the mixture was extracted thrice with CH_2_Cl_2_ (30.0 mL). The combined organic phases were dried over MgSO_4_ and reduced in vacuum. The crude compound was purified flash column chromatography on silica (eluent isohexane/ethyl acetate 3 : 7). Amorphous colourless solid (173 mg, 96 %).

**Methyl 2‐hydroxy‐2,2‐bis(4‐methoxyphenyl)acetate (8 k)**: A mixture of 4,4′‐dimethoxybenzilic acid (2.78 g, 9.64 mmol) and 1,8‐diazabicyclo[5.4.0]undec‐7‐ene (DBU, 2.65 g, 17.4 mmol) in MeCN (4.5 mL) was cooled to 0 °C and iodomethane (5.02 g, 35.0 mmol) was added. The ice bath was removed and stirring was continued for 48 h at ambient temperature. The reaction mixture was reduced in vacuum and purified by flash column chromatography on silica (eluent pentane/Et_2_O 2 : 1 to 1 : 1). Amorphous colourless solid (2.64 g, 91 %).

**4‐Chloro‐1,1‐bis(4‐methoxyphenyl)butan‐1‐ol (8 l)**: GP1 was followed using a Grignard reagent prepared from 4‐bromoanisol (0.94 g, 4.98 mmol) and magnesium (122 mg, 5.02 mmol) in THF (12.0 mL). A solution of 4‐chloro‐1‐(4‐methoxyphenyl)butan‐1‐one (1.06 g, 5.00 mmol) in THF (12.0 mL) was added dropwise at 0 °C. The reaction mixture was stirred 1 h at ambient temperature. The crude compound was purified by flash column chromatography on silica (eluent isohexane/ethyl acetate 9 : 1). Colourless oil (1.06 g, 66 %).

**4‐Bromo‐N,N‐dimethylbenzenesulfonamide (9 d)**: A mixture of 4‐bromobenzenesulfonamide (2.40 g, 10.2 mmol), tetrabutylammonium tetrafluoroborate (329 mg, 1.02 mmol), K_2_CO_3_ (5.58 g, 40.4 mmol) and dimethyl sulfate (2.53 g, 20.0 mmol) in MeCN (15.0 mL) was refluxed for 3 h. After cooling to room temperature concentrated ammonium hydroxide solution (5.0 mL) was added and stirring was continued for 16 h. The reaction mixture was poured into water (20.0 mL) and extracted thrice with CH_2_Cl_2_ (30.0 mL). The combined organic phases were dried over MgSO_4_, filtered and reduced in vacuum. The crude compound was purified by flash column chromatography on silica (eluent pentane/Et_2_O 7 : 3). Amorphous colourless solid (2.66 g, 99 %).

**1‐{4‐[Hydroxybis(4‐methoxyphenyl)methyl]phenyl}ethan‐1‐one (11)**: Concentrated hydrochloric acid (3.0 mL) was added to a solution of **8 b** (430 mg, 1.02 mmol) in THF (12.0 mL). The mixture was refluxed overnight, basified with 2 M NaOH and extracted thrice with CH_2_Cl_2_. The combined organic phases were dried over MgSO_4_, filtered and reduced in vacuum. The crude compound was purified by flash column chromatography on silica (eluent pentane/Et_2_O 1 : 1). Amorphous colourless solid (345 mg, 93 %).

***rac‐*** **Ethyl 1‐{2‐[(4‐formylphenyl)bis(4‐methoxyphenyl)methoxy]ethyl}‐piperidine‐3‐carboxylate (*rac*‐12 a)**: GP3 was followed using **8 a** (2.68 g, 6.35 mmol), acetyl chloride (10.0 mL), *rac*‐ethyl 1‐(2‐hydroxyethyl)nipecotinate (1.41 g, 6.99 mmol), K_2_CO_3_ (2.20 g, 15.9 mmol), acetonitrile (20.0 mL). The crude compound was purified by two consecutive flash column chromatographic runs on silica (eluent pentane/Et_2_O 1 : 1+5 % triethyl amine; Et_2_O 100 %). Colourless oil (2.35 g, 70 %).

**(*S*)‐Ethyl 1‐{2‐[(4‐formylphenyl)bis(4‐methoxyphenyl)methoxy]ethyl}‐piperidine‐3‐carboxylate [(*S*)‐12 a]**: GP3 was followed using **8 a** (509 mg, 1.21 mmol), acetyl chloride (3.0 mL), (S)‐ethyl 1‐(2‐hydroxyethyl)nipecotinate (318 mg, 1.58 mmol), K_2_CO_3_ (498 mg, 3.60 mmol), MeCN (5.0 mL). The crude compound was purified by two consecutive flash column chromatographic runs on silica (eluent pentane/Et_2_O 1 : 1+5 % triethyl amine; Et_2_O 100 %). Colourless oil (324 mg, 41 %).

**(*R*)‐Ethyl 1‐{2‐[(4‐formylphenyl)bis(4‐methoxyphenyl)methoxy]ethyl}‐piperidine‐3‐carboxylate [(*S*)‐12 a]**: GP3 was followed using **8 a** (380 mg, 0.900 mmol), acetyl chloride (1.0 mL), (*R*)‐ethyl 1‐(2‐hydroxyethyl)nipecotinate (201 mg, 1.00 mmol), K_2_CO_3_ (311 mg, 2.25 mmol), MeCN (1.0 mL). The crude compound was purified by two consecutive flash column chromatographic runs on silica (eluent pentane/Et_2_O 1 : 1+5 % triethyl amine;Et_2_O 100 %). Colourless oil (270 mg, 56 %).

***rac‐*** **Ethyl 1‐{2‐[(4‐acetylphenyl)bis(4‐methoxyphenyl)methoxy]ethyl}‐piperidine‐3‐carboxylate (12 b)**: GP3 was followed using **8 b** (344 mg, 0.950 mmol), acetyl chloride (3.0 mL), *rac*‐ethyl 1‐(2‐hydroxyethyl)nipecotinate (211 mg, 1.05 mmol), K_2_CO_3_ (329 mg, 2.38 mmol), MeCN (3.0 mL). The crude compound was purified by flash column chromatography on silica (eluent pentane/Et_2_O 6 : 4+5 % trimethylamine). Colourless oil (261 mg, 50 %).

***rac‐*** **Ethyl 1‐(2‐{[4‐(N,N‐dimethylsulfamoyl)phenyl]bis[4‐methoxyphenyl]‐methoxy}ethyl)piperidine‐3‐carboxylate (12 c)**: GP3 was followed using **8 c** (455 mg, 1.06 mmol), acetyl chloride (3.0 mL), *rac*‐ethyl 1‐(2‐hydroxyethyl)nipecotinate (213 mg, 1.06 mmol), K_2_CO_3_ (366 mg, 2.65 mmol), MeCN (3.0 mL). The crude compound was purified by flash column chromatography on silica (eluent pentane/Et_2_O 1 : 1+5 % trimethylamine). Yellowish semi‐solid (411 mg, 64 %).

***rac‐*** **Ethyl 1‐(2‐{imidazo[1,2‐a]pyridin‐6‐ylbis(4‐methoxyphenyl)methoxy}‐ethyl)piperidine‐3‐carboxylate (12 d)**: GP5 was followed using **8 d** (254 mg, 0.710 mmol), acetyl chloride (3.0 mL), *rac*‐ethyl 1‐(2‐hydroxyethyl)nipecotinate (157 mg, 0.780 mmol), K_2_CO_3_ (488 mg, 3.53 mmol), MeCN (5.0 mL). The crude compound was purified by flash column chromatography on silica (eluent Et_2_O/MeOH 9 : 1). Yellow semi‐solid (160 mg, 48 %).

***rac‐*** **Ethyl 1‐(2‐{[4‐(ethoxycarbonyl)phenyl]bis[4‐methoxyphenyl]‐methoxy}ethyl)piperidine‐3‐carboxylate (12 e)**: GP3 was followed using **8 e** (217 mg, 0.550 mmol), acetyl chloride (1.0 mL), *rac*‐ethyl 1‐(2‐hydroxyethyl)nipecotinate (133 mg, 0.661 mmol), K_2_CO_3_ (191 mg, 1.38 mmol), MeCN (2.0 mL). The crude compound was purified by two consecutive flash column chromatographic runs on silica (eluent pentane/Et_2_O 4 : 6; pentane/Et_2_O 8 : 2+5 % dimethylethylamine). Colourless semi‐solid (173 mg, 54 %).

***rac‐*** **Ethyl 1‐(2‐{[4‐(methoxymethyl)phenyl]bis[4‐methoxyphenyl]‐methoxy}ethyl)piperidine‐3‐carboxylate (12 f)**: GP3 was followed using **8 f** (310 mg, 0.850 mmol), acetyl chloride (5.0 mL), *rac*‐ethyl 1‐(2‐hydroxyethyl)nipecotinate (191 mg, 0.949 mmol), K_2_CO_3_ (294 mg, 2.13 mmol), MeCN (5.0 mL). The crude compound was purified by two consecutive flash column chromatographic runs on silica (eluent Et_2_O 100 %, pentane/Et_2_O 7 : 3+5 % triethylamine). Colourless oil (207 mg, 44 %).

***rac‐*** **Ethyl 1‐{2‐[(4‐cyanophenyl)bis(4‐methoxyphenyl)methoxy]ethyl}‐piperidine‐3‐carboxylate (12 g)**: GP3 was followed using **8 g** (364 mg, 1.00 mmol), acetyl chloride (1.0 mL), *rac*‐ethyl 1‐(2‐hydroxyethyl)nipecotinate (242 mg, 1.20 mmol), K_2_CO_3_ (346 mg, 2.50 mmol), MeCN (5.0 mL). The crude compound was purified by flash column chromatography on silica (eluent pentane/Et_2_O/triethyl amine 4 : 4 : 2). Yellowish semi‐solid (447 mg, 85 %).

***rac‐*** **Ethyl 1‐(2‐{bis[4‐(methoxymethyl)phenyl][4‐methoxyphenyl]‐methoxy}ethyl)piperidine‐3‐carboxylate (12 h)**: GP3 was followed using **8 h** (380 mg, 1.00 mmol), acetyl chloride (3.0 mL), *rac*‐ethyl 1‐(2‐hydroxyethyl)nipecotinate (221 mg, 1.10 mmol), K_2_CO_3_ (346 mg, 2.5 mmol), MeCN (5.0 mL). The crude compound was purified flash column chromatography on silica (eluent Et_2_O). Colourless oil (260 mg, 46 %).

***rac‐*** **Ethyl 1‐(2‐{tris[4‐(methoxymethyl)phenyl]methoxy}ethyl)piperidine‐3‐carboxylate (12 i)**: GP3 was followed using **8 i** (243 mg, 0.620 mmol), acetyl chloride (3.0 mL), *rac* ethyl 1‐(2‐hydroxyethyl)nipecotinate (452 mg, 2.25 mmol), K_2_CO_3_ (774 mg, 5.60 mmol), MeCN (5.0 mL). The crude compound was purified by flash column chromatography on silica (eluent isohexane/Et_2_O 8 : 1 to Et_2_O 100 %). Colourless oil (151 mg, 42 %).

***rac‐*** **Ethyl 1‐{2‐[(4‐carbamoylphenyl)bis(4‐methoxyphenyl)methoxy]‐ethyl}piperidine‐3‐carboxylate (12 j)**: GP3 was followed using **8 j** (512 mg, 1.41 mmol), acetyl chloride (5.0 mL), *rac‐*ethyl 1‐(2‐hydroxyethyl)nipecotinate (321 mg, 1.55 mmol), K_2_CO_3_ (488 mg, 3.53 mmol), MeCN (5.0 mL). The crude compound was purified flash column chromatography on silica (eluent ethyl acetate+5 % MeOH). Amorphous slightly yellowish solid (375 mg, 49 %).

***rac‐*** **Ethyl 1‐{2‐[2‐methoxy‐1,1‐bis(4‐methoxyphenyl)‐2‐oxoethoxy]ethyl}‐piperidine‐3‐carboxylate (12 k)**: GP3 was followed using **8 k** (680 mg, 2.24 mmol), acetyl chloride (5.0 mL), *rac*‐ethyl 1‐(2‐hydroxyethyl)nipecotinate (452 mg, 2.25 mmol), K_2_CO_3_ (774 mg, 5.60 mmol), MeCN (5.0 mL). The crude compound was purified by flash column chromatography on silica (eluent pentane/Et_2_O 1 : 1 to pentane/Et_2_O 1 : 1+20 % triethylamine). Colourless semi‐solid (956 mg, 88 %).

***rac*** **‐Ethyl 1‐[4‐hydroxy‐4,4‐bis(4‐methoxyphenyl)butyl]piperidine‐3‐carboxylate (12 l)**: A mixture of **8 l** (435 mg, 1.29 mmol), *rac‐*ethyl nipecotinate (233 mg, 1.42 mmol), K_2_CO_3_ (446 mg, 3.23 mmol) and KI (21 mg, 0.13 mmol) in MeCN (5.0 mL) was irradiated in the microwave at 80 °C for 24 h, filtered and reduced in vacuum. The crude compound was purified by flash column chromatography on silica (eluent pentane/Et_2_O 2 : 1+5 % triethylamine). Amorphous slightly yellowish solid (374 mg, 66 %).

***rac*** **‐Ethyl 1‐(2‐{[4‐(hydroxymethyl)phenyl]bis[4‐methoxyphenyl]methoxy}‐ethyl)piperidine‐3‐carboxylate (12 m)**: Sodium borohydride (46 mg, 1.2 mmol) was added to a solution of **12 a** (254 mg, 0.480 mmol) in MeOH (7.5 mL) at 0 °C. The ice bath was removed and stirring was continued at ambient temperature for 3 h. The mixture was poured into water (30.0 mL) and extracted thrice with Et_2_O (30.0 mL). The combined organic phases were dried over MgSO_4_, filtered and reduced in vacuum. Colourless oil (226 mg, 89 %).

***rac*** **‐Ethyl 1‐{2‐[(4‐{[(2‐methoxy‐2‐oxoethyl)amino]methyl}phenyl)bis(4‐methoxyphenyl)methoxy]ethyl}piperidine‐3‐carboxylate (12 n)**: GP5 was followed using **12 a** (449 mg, 0.850 mmol), methyl glycinate hydrochloride (212 mg, 1.69 mmol), sodium triacetoxyborohydride (250 mg, 1.18 mmol), CH_2_Cl_2_ (5.0 mL). The crude compound was purified by flash column chromatography on silica (eluent isohexane/ethyl acetate 7 : 3+5 % triethylamine). Colourless semi‐solid (289 mg, 57 %).

***rac*** **‐Ethyl 1‐{2‐[(4‐{[(3‐ethoxy‐3‐oxopropyl)amino]methyl}phenyl)bis(4‐methoxyphenyl)methoxy]ethyl}piperidine‐3‐carboxylate (12 o)**: GP5 was followed using **12 a** (480 mg, 0.900 mmol), β‐alanine methyl ester hydrochloride (314 mg, 2.25 mmol), sodium triacetoxyborohydride (267 mg, 1.26 mmol), CH_2_Cl_2_ (10.0 mL). The crude compound was purified by flash column chromatography on silica (eluent Et_2_O+4 % triethylamine). Pale yellow oil (236 mg, 42 %).

***rac‐*** **Ethyl 1‐(2‐{[4‐({[1‐(ethoxycarbonyl)cyclopropyl]amino}methyl)‐phenyl]bis[4‐methoxyphenyl]methoxy}ethyl)piperidine‐3‐carboxylate (12 p)**: GP5 was followed using **12 a** (233 mg, 0.44 mmol), ethyl 1‐aminocyclopropanecarboxylate hydrochloride (145 mg, 0.88 mmol), sodium triacetoxyborohydride (131 mg, 0.620 mmol), CH_2_Cl_2_ (2.0 mL). The crude compound was purified by flash column chromatography on silica (eluent pentane/Et_2_O+4 % triethylamine). Colourless semi‐solid (168 mg, 59 %).

***rac‐*** **Ethyl 1‐{2‐[(4‐{[(1‐ethoxy‐2‐methyl‐1‐oxopropan‐2‐yl)amino]methyl}‐phenyl)bis(4‐methoxyphenyl)methoxy]ethyl}piperidine‐3‐carboxylate (12 q)**: GP5 was followed using **12 a** (152 mg, 0.290 mmol), ethyl 2‐amino‐2‐methylpropanoate hydrochloride (96 mg, 0.57 mmol), sodium triacetoxyborohydride (85 mg, 0.40 mmol), CH_2_Cl_2_ (2.0 mL). The crude compound was purified by flash column chromatography on silica (eluent pentane/Et_2_O 1 : 1+4 % triethylamine). Colourless semi‐solid (119 mg, 64 %).

***rac*** **‐4‐{[4‐({2‐[3‐(Ethoxycarbonyl)piperidin‐1‐yl]ethoxy}bis{4‐methoxy‐phenyl}methyl)benzyl]amino}butanoic acid (12 s)**: GP6 was followed 4‐aminobutanoic acid (83 mg, 0.80 mmol), **12 a** (213 mg, 0.40 mmol), sodium cyanoborohydride (37 mg, 0.56 mmol), MeOH (5.0 mL). The crude compound was purified by flash column chromatography on silica (eluent MeOH). Amorphous colourless solid (100 mg, 40 %).

***rac‐*** **4‐{[4‐({2‐[3‐(Ethoxycarbonyl)piperidin‐1‐yl]ethoxy}bis{4‐methoxy‐phenyl}methyl)benzyl]amino}benzoic acid (12 t)**: GP6 was followed using 4‐aminobutanoic acid (217 mg, 1.58 mmol), **12 a** (420 mg, 0.790 mmol), sodium cyanoborohydride (73 mg, 1.1 mmol), MeOH (10.0 mL). The crude compound was purified by flash column chromatography on silica (eluent MeCN/H_2_O 9 : 1). Amorphous slightly yellowish solid (132 mg, 25 %).

### Analytical data

The analytical data of the synthesized compounds **5 a**–**t**, **8 a**–**l**, **9 c**, **11**, **12 a**–**q** and **12 s**–**t** are presented in the Supporting Information.

### Biological evaluation

**[^3^H]GABA uptake assays**: The [^3^H]GABA uptake assays were performed according to Kragler et al.^33^ Cells were grown in 150 cm^2^ plates to a confluence of 70–90 % up to passage 25. After detachment the cells were centrifuged 5 min at 500×g, washed for three times in phosphate buffered saline and, finally, resuspended in Krebs buffer (2.5 mM CaCl_2_, 1.2 mM MgSO_4_, 1.2 mM KH_2_PO_4_, 4.7 mM KCl, 11 mM glucose, 25 mM Tris, 119 mM NaCl, pH 7.2). The uptake assays were performed with aliquots of the resulting cell suspension (about 100000–200000 cells per well) in a total volume of 250 μl in 96 well 2.2 mL deep well plates. The cells were equilibrated for 25 min in Krebs buffer in the presence of the test compound at 37 °C in a gently shaking water bath. In the case of poor solubility of the test compounds all samples contained 1 % DMSO. After addition of 25 μl of a solution containing [^3^H]GABA (2.22 TBq/mmol, ARC, St Louis, MO, USA) and unlabeled GABA in Krebs (final concentration 8 nM [^3^H]GABA and 32 nM unlabeled GABA for mGAT1, mGAT3, mGAT4 and 20 nM [^3^H]GABA and 20 nM unlabeled GABA for mGAT2) the cells were incubated for further 4 min (mGAT1, mGAT3, mGAT4) or 10 minutes (mGAT2), respectively. The incubation was stopped by filtration through Sartorius MGC micro glass fiber filters pre‐soaked for 1 h in 0.9 % NaCl by means of a Brandell MWXR‐96TI cell harvester (Brandell, Gaithersburg, MD, USA) under reduced pressure. The filters were rinsed two times with cold 0.9 % NaCl and subsequently transferred to 96 well sample plates (Perkin Elmer LAS, Boston, MA, USA). After addition of 200 μl Rotiszint Eco Plus (Roth, Karlsruhe, Germany) per well the radioactivity was determined in a microbeta liquid scintillation counter (Perkin Elmer LAS, Boston, MA, USA). Non‐specific uptake was determined in presence of 10 mM GABA for mGAT1, mGAT3 and mGAT4 or 100 mM GABA for mGAT2, respectively. The results are expressed as means±S.E.M. of at least three separate experiments, each carried out in triplicate.

**MS Binding Assays**: The MS Binding Assays were performed with mGAT1 membrane preparations obtained from a stably mGAT1 expressing HEK293 cell line and NO711 as unlabeled marker in competitive binding experiments as previously described in detail.^34^ In short, test compounds were investigated at 7 concentrations in presence of 10 nM NO 711 in competition experiments. Non specific binding was defined as binding remaining in the presence of 100 mM GABA. Incubation was terminated by transfer of 200 μL per well onto a 96‐well filter plate (Acroprep, glass fibre, 1.0 μm, 350 μL, Pall, Dreieich, Germany) with a 12 channel pipette. After rapid vacuum‐filtration the filters were washed with ice cold 0.9 % NaCl (5×150 μL) and dried at 50 °C for 60 min. Afterwards, the filters were eluted with 100 μL methanol containing [^2^H_10_]NO711 (1.4 nM) as internal standard per well into a deep‐well plate by application of vacuum. This step was repeated twice resulting in a total elution volume of 300 μL. Finally, a volume of 130 μL ammonium formate buffer (10 mM, pH 7.0) was added per well and the generated samples analyzed by LC‐ESI‐MS/MS using a API 3200 as described in detail previously.^40^

## Conflict of interest

The authors declare no conflict of interest.

## Supporting information

As a service to our authors and readers, this journal provides supporting information supplied by the authors. Such materials are peer reviewed and may be re‐organized for online delivery, but are not copy‐edited or typeset. Technical support issues arising from supporting information (other than missing files) should be addressed to the authors.

Supplementary
