# Supplementary material for: N‐Substituted Nipecotic Acids as (S)‐SNAP‐5114 Analogues with Modified Lipophilic Domains
Source: ChemMedChem. 2020 Apr 7;15(9):756–71. doi: 10.1002/cmdc.201900719 (PMC7317212; doi:10.1002/cmdc.201900719)
Supplement: Supplementary file 1 — Supplementary [file CMDC-15-756-s001.pdf]

ChemMedChem

Supporting Information

N-Substituted Nipecotic Acids as (*S*)-SNAP-5114 Analogues with Modified Lipophilic Domains

Michael C. Böck, Georg Höfner, and Klaus T. Wanner*© 2020 The Authors. Published by Wiley-VCH Verlag GmbH & Co. KGaA. This is an open access article under the terms of the Creative Commons Attribution License, which permits use, distribution and reproduction in any medium, provided the original work is properly cited.

Supporting Information

Contents:

1. pIC_{50} values determined for **5a** and the reference compound (*S*)-SNAP-5114 [(*S*)-**2**] in relation to various preincubation times.

2. Analytical data for compounds **5a-t**, **8a-l**, **9c**, **11**, **12a-q** and **12s-t**.

1. pIC$_{50}$ values determined for **5a** and the reference compound (*S*)-SNAP-5114 [(*S*)-**2**] in relation to various preincubation times

Table 1: pIC$_{50}$ values of **5a** and (*S*)-SNAP-5114 [(*S*)-**2**] at various incubation times.

Experiment No.	Preincubation time [min] 10	25	25	60	60
1	5.87 (**5a**)	5.98 (**5a**)		6.04 (**5a**)	
	5.68 [(*S*)-**2**]	5.97 [(*S*)-**2**]		5.97 [(*S*)-**2**]	
2	5.57 (**5a**)	5.73 (**5a**)		5.91 (**5a**)	
	5.53 [(*S*)-**2**]	5.59 [(*S*)-**2**]		5.57 [(*S*)-**2**]	
3	5.57 (**5a**)	5.83 (**5a**)		5.78 (**5a**)	
	5.44 [(*S*)-**2**]	5.57 [(*S*)-**2**]		5.80 [(*S*)-**2**]	
4			5.70 (**5a**)		6.21 (**5a**)
			5.72 [(*S*)-**2**]		6.13 [(*S*)-**2**]
5			5.80 (**5a**)		5.90 (**5a**)
			6.43 [(*S*)-**2**]		6.53 [(*S*)-**2**]
6			5.86 (**5a**)		5.99 (**5a**)
			6.05 [(*S*)-**2**]		6.23 [(*S*)-**2**]
Average	5.67 (**5a**)	5.85 (**5a**)	5.79 (**5a**)	5.91 (**5a**)	6.03 (**5a**)
	5.55 [(*S*)-**2**]	5.71 [(*S*)-**2**]	6.07 [(*S*)-**2**]	5.78 [(*S*)-**2**]	6.30 [(*S*)-**2**]
Normalized	97.98 (**5a**)	100.00 (**5a**)	100.00 (**5a**)	101.08 (**5a**)	104.26 (**5a**)
	97.20 [(*S*)-**2**]	100.00 [(*S*)-**2**]	100.00 [(*S*)-**2**]	101.23 [(*S*)-**2**]	103.79 [(*S*)-**2**]

2. Analytical data for compounds **5a-t**, **8a-l**, **9c**, **11**, **12a-q** and **12s-t**

***rac*-1-{2-[(4-Formylphenyl)bis(4-methoxyphenyl)methoxy]ethyl}piperidine-3-carboxylic acid (5a):** ^{1}H NMR (400 MHz, MeOD + NaOD) δ = 1.25 – 1.41 (m, 1H, $NCH_2CH_2CH_{ax}H_{eq}$), 1.54 (qt, *J*=12.1, 3.9, 1H, $NCH_2CH_{ax}H_{eq}CH_2$), 1.61 – 1.70 (m, 1H, $NCH_2CH_{ax}H_{eq}CH_2$), 1.88 – 2.02 (m, 2H, $NCH_2CH_2CH_{ax}H_{eq}$ + $NCH_{ax}H_{eq}CH_2CH_2$), 2.08 (t, *J*=11.3, 1H, $NCH_{ax}H_{eq}CHCOO$), 2.34 (tt, *J*=11.6, 3.7, 1H, NCH_2CHCOO), 2.63 (t, *J*=6.3, 2H, NCH_2CH_2O), 2.82 (d, *J*=11.3, 1H, $NCH_{ax}H_{eq}CH_2CH_2$), 2.99 – 3.07 (m, 1H, $NCH_{ax}H_{eq}CHCOO$), 3.18 – 3.31 (m, 2H, NCH_2CH_2O), 3.78 (s, 6H, $C_{Ar}OCH_3$), 6.81 – 6.91 (m, 4H, $CCHCHCOCH_3$), 7.23 – 7.34 (m, 4H, $CCHCHCOCH_3$), 7.54 – 7.64 (m, 2H, CC*H*CHCCHO), 7.70 (d, *J*=8.2, 2H, CCHC*H*CCHO), 8.70 (br s, 1H, C*H*O). ^{13}C NMR (101 MHz, CD_2Cl_2) δ = 22.56 ($NCH_2CH_2CH_2$), 27.08 ($NCH_2CH_2CH_2$), 40.90 (NCH_2CHCOO), 53.32 ($NCH_2CH_2CH_2$), 55.81 ($C_{Ar}OCH_3$), 56.10 (NCH_2CHCOO), 57.70 (NCH_2CH_2O), 59.97 (NCH_2CH_2O), 87.02 (NCH_2CH_2OC), 113.87 ($CCHCHCOCH_3$), 128.66 (C*C*HCHCCHO), 129.77 (CCH*C*HCCHO), 130.84 (d, *J*=3.6, $CCHCHCOCH_3$), 135.10 (d, *J*=5.5, $CCHCHCOCH_3$), 135.51 (CCHCH*C*CHO), 153.17 (*C*CHCHCCHO), 159.54 ($CCHCHCOCH_3$), 176.74 (*C*OO), 192.29 (CHO). IR (KBr): ṽ = 2933, 2835, 1702, 1606, 1574, 1509, 1463, 1410, 1303, 1251, 1213, 1175, 1153, 1115, 1068, 1033, 916, 822, 732, 677, 582, 536 cm^{-1}. HRMS-ESI- *m/z* $[M-H]^-$ calcd for $C_{30}H_{33}NO_6$: 502.2235, found: 502.2241.

(*S*)-1-{2-[(4-Formylphenyl)bis(4-methoxyphenyl)methoxy]ethyl}piperidine-3-carboxylic acid [(*S*)-5a]: The analytical data are consistent with racemic compound **5a**. $[\alpha]_D^{22}$ = +0.14 (c=1.93 g/100 ml in EtOH).

(*R*)-1-{2-[(4-Formylphenyl)bis(4-methoxyphenyl)methoxy]ethyl}piperidine-3-carboxylic acid [(*R*)-5a]: The analytical data are consistent with racemic compound **5a**. $[\alpha]_D^{22}$ = -0.15 (c=2.03 g/100 ml in EtOH).

***rac*-1-{2-[(4-Acetylphenyl)bis(4-methoxyphenyl)methoxy]ethyl}piperidine-3-carboxylic acid (5b):** ^{1}H NMR (400 MHz, MeOD + NaOD) δ = 1.25 – 1.41 (m, 1H, $NCH_2CH_2CH_{ax}H_{eq}$), 1.48 – 1.63 (m, 1H, $NCH_2CH_{ax}H_{eq}CH_2$), 1.63 – 1.73 (m, 1H, $NCH_2CH_{ax}H_{eq}CH_2$), 1.92 – 2.05 (m, 2H, $NCH_2CH_2CH_{ax}H_{eq}$ + $NCH_{ax}H_{eq}CH_2CH_2$), 2.10 (t, *J*=11.3, 1H, $NCH_{ax}H_{eq}CHCOO$), 2.36 (ddt, *J*=12.0, 7.4, 3.9, 1H, NCH_2CHCOO), 2.66 (t, *J*=6.3, 2H, NCH_2CH_2O), 2.85 (d, *J*=11.4, 1H, $NCH_{ax}H_{eq}CH_2CH_2$), 3.03 – 3.11 (m, 1H, $NCH_{ax}H_{eq}CHCOO$), 3.21-3.31 (m, 2H, NCH_2CH_2O), 3.80 (s, 6H, $C_{Ar}OCH_3$), 6.85 – 6.93 (m, 4H, $CCHCHCOCH_3$), 7.27 – 7.37 (m, 4H, $CCHCHCOCH_3$), 7.58 – 7.66 (m, 2H, $CCHCHCCCH_3$), 7.90 – 7.95 (m, 2H, $CCHCHCCCH_3$). ^{13}C NMR (101 MHz, MeOD + NaOD) δ = 25.98 ($NCH_2CH_2CH_2$), 29.33 ($NCH_2CH_2CH_2$), 46.38 (NCH_2CHCOO), 55.49 ($NCH_2CH_2CH_2$), 55.80 ($C_{Ar}OCH_3$), 58.80 (NCH_2CHCOO), 59.49 (NCH_2CH_2O), 62.51 (NCH_2CH_2O), 87.60 (NCH_2CH_2OC), 114.30 ($CCHCHCOCH_3$), 128.08 ($CCHCHCCCH_3$), 128.09 ($CCHCHCCCH_3$), 131.46 ($CCHCHCOCH_3$), 136.15 ($CCHCHCOCH_3$), 136.35 ($CCHCHCCCH_3$), 153.14 ($CCHCHCCCH_3$), 160.37 ($CCHCHCOCH_3$), 182.81 ($COOCH_2CH_3$), 200.68 (CCH_3). IR (KBr): ṽ = 2934, 2361, 2342, 1683, 1606, 1509, 1407, 1251, 1176, 1070, 1034, 828, 668, 605 cm^{-1}. HRMS-ESI+ *m/z* $[M+H]^+$ calcd for $C_{31}H_{35}NO_6$: 518.2534, found: 518.2537.

***rac*-1-(2-{[4-(N,N-dimethylsulfamoyl)phenyl]bis[4-methoxyphenyl]methoxy}ethyl)piperidine-3-carboxylic acid (5c):** ^{1}H NMR (500 MHz, MeOD + NaOD) δ = 1.25 – 1.41 (m, 1H, $NCH_2CH_2CH_{ax}H_{eq}$), 1.55 (qt, *J*=13.0, 3.9, 1H, $NCH_2CH_{ax}H_{eq}CH_2$), 1.62 – 1.70 (m, 1H, $NCH_2CH_{ax}H_{eq}CH_2$), 1.92 – 2.02 (m, 2H, $NCH_2CH_2CH_{ax}H_{eq}$ + $NCH_{ax}H_{eq}CH_2CH_2$), 2.08 (t, *J*=11.4, 1H, $NCH_{ax}H_{eq}CHCOO$), 2.35 (tt, *J*=11.8, 3.7, 1H, NCH_2CHCOO), 2.61 – 2.69 (m,

8H, NCH_2CH_2O + SO_2NCH_3), 2.79 – 2.85 (m, 1H, $NCH_{ax}H_{eq}CH_2CH_2$), 3.06 (dt, *J*=11.4, 1.8, 1H, $NCH_{ax}H_{eq}CHCOO$), 3.21 – 3.30 (m, 2H, NCH_2CH_2O), 3.79 (s, 6H, $C_{Ar}OCH_3$), 6.85 – 6.93 (m, 4H, $CCHCHCOCH_3$), 7.26 – 7.34 (m, 4H, $CCHCHCOCH_3$), 7.66 – 7.77 (m, 4H, CCHCHCS + CCHCHCS). ^{13}C NMR (126 MHz, MeOD + NaOD) δ = 25.99 ($NCH_2CH_2CH_2$), 29.33 ($NCH_2CH_2CH_2$), 38.30 (NSO_2CH_3), 46.38 (NCH_2CHCOO), 54.49 ($NCH_2CH_2CH_2$), 55.82 ($C_{Ar}OCH_3$), 58.80 (NCH_2CHCOO), 59.46 (NCH_2CH_2O), 62.56 (NCH_2CH_2O), 87.48 (NCH_2CH_2OC), 114.42 ($CCHCHCOCH_3$), 128.45 (CCHCHCS), 129.49 (CCHCHCS), 131.51($CCHCHCOCH_3$), 134.64 (CCHCHCS), 135.98 ($CCHCHCOCH_3$), 153.19 (CCHCHCS), 160.48 ($CCHCHCOCH_3$), 182.82 (COO). IR (KBr): ṽ = 2934, 2361, 2341, 1607, 1578, 1508, 1460, 1411, 1342, 1251, 1165, 1033, 953, 829, 753, 693, 599, 586 cm^{-1}. HRMS-ESI+ *m/z* $[M+H]^+$ calcd for $C_{31}H_{38}N_2O_7S$: 583.2473, found: 583.2474.

***rac*-1-(2-{Imidazo[1,2-a]pyridin-6-ylbis(4-methoxyphenyl)methoxy}ethyl)piperidine-3-carboxylic acid (5d):** ^1H NMR (500 MHz, MeOD + NaOD) δ = 1.25 – 1.39 (m, 1H, $NCH_2CH_2CH_{ax}H_{eq}$), 1.56 (qt, *J*=13.0, 3.8, 1H, $NCH_2CH_{ax}H_{eq}CH_2$), 1.64 – 1.70 (m, 1H, $NCH_2CH_{ax}H_{eq}CH_2$), 1.93 – 2.03 (m, 2H, $NCH_2CH_2CH_{ax}H_{eq}$ + $NCH_{ax}H_{eq}CH_2CH_2$), 2.10 (t, *J*=11.4, 1H, $NCH_{ax}H_{eq}CHCOO$), 2.37 (tt, *J*=11.9, 3.7, 1H, NCH_2CHCOO), 2.62 – 2.72 (m, 2H, NCH_2CH_2O), 2.82 (br d, *J*=11.3, 1H, $NCH_{ax}H_{eq}CH_2CH_2$), 3.09 – 3.15 (m, 1H, $NCH_{ax}H_{eq}CHCOO$), 3.32 – 3.36 (m, 2H, NCH_2CH_2O), 3.79 (s, 6H, $C_{Ar}OCH_3$), 6.88 – 6.94 (m, 4H, $CCHCHCOCH_3$), 7.13 (dd, *J*=9.6, 1.8, 1H, CCHCHCN), 7.33 – 7.40 (m, 5H, $CCHCHCOCH_3$ + CCHCHCN), 7.52 (d, *J*=1.4, 1H, CCHNCHCHN), 7.86 (dd, *J*=1.4, 0.7, 1H, CCHNCHCHN), 8.69 (dd, *J*=1.9, 1.0, 1H, CCHNCHCHN). ^{13}C NMR (126 MHz, MeOD + NaOD) δ = 25.97 ($NCH_2CH_2CH_2$), 29.34 ($NCH_2CH_2CH_2$), 46.34 (NCH_2CHCOO), 55.46 ($NCH_2CH_2CH_2$), 55.83 ($C_{Ar}OCH_3$), 58.79 (NCH_2CHCOO), 59.47 (NCH_2CH_2O), 62.46 (NCH_2CH_2O), 86.23 (NCH_2CH_2OC), 114.51 ($CCHCHCOCH_3$), 115.23 (CCHNCHCHN), 116.24 (CCHCHCN), 126.20 (CCHNCHCHN), 128.27 (CCHCHCN), 130.43 ($CCHCHCOCH_3$), 133.17 (CCHNCHCHN), 133.49 (CCHNCHCHN), 135.38 ($CCHCHCOCH_3$), 145.38 (CCHCHCN), 160.58 ($CCHCHCOCH_3$), 182.82 (COO). IR (KBr): ṽ = 2936, 1608, 1578, 1508, 1458, 1397, 1315, 1251, 1177, 1131, 1085, 1031, 925, 829, 732, 676, 621, 586 cm^{-1}. HRMS-ESI- *m/z* $[M-H]^-$ calcd for $C_{30}H_{33}O_5N_3$: 514.2347, found: 514.2352.

***rac*-1-{2-[(4-Carboxyphenyl)bis(4-methoxyphenyl)methoxy]ethyl}piperidine-3-carboxylic acid (5e):** ^1H NMR (500 MHz, D_2O + NaOD) δ = 0.88 – 1.02 (m, 1H, $NCH_2CH_2CH_{ax}H_{eq}$), 1.05 – 1.17 (m, 1H, $NCH_2CH_{ax}H_{eq}CH_2$), 1.18 – 1.27 (m, 1H, $NCH_2CH_{ax}H_{eq}CH_2$), 1.41 (t, *J*=11.3, 1H, $NCH_{ax}H_{eq}CH_2CH_2$), 1.63 (br d, *J*=11.7, 1H, $NCH_2CH_2CH_{ax}H_{eq}$), 1.75 (t, *J*=11.5, 1H, $NCH_{ax}H_{eq}CHCOO$), 1.99 – 2.20 (m, 2H, NCH_2CHCOO + $NCH_{ax}H_{eq}CH_2CH_2$), 2.25 – 2.37 (m, 2H, NCH_2CH_2O), 2.68 (br d, *J*=10.7, 1H, $NCH_{ax}H_{eq}CHCOO$), 2.95 (br s, 2H, NCH_2CH_2O), 3.30 (s, 6H, $C_{Ar}OCH_3$), 6.38 – 6.52 (m, 4H, $CCHCHCOCH_3$), 7.02 (br d, *J*=8.4, 4H, $CCHCHCOCH_3$), 7.21 (br d, *J*=8.1, 2H, CCHCHCCOO), 7.61 (br d, *J*=8.1, 2H, CCHCHCCOO). ^{13}C NMR (126 MHz, D_2O + NaOD) δ = 23.89 ($NCH_2CH_2CH_2$), 27.40 ($NCH_2CH_2CH_2$), 44.34 (NCH_2CHCOO), 52.53 ($NCH_2CH_2CH_2$), 54.73 (d, $C_{Ar}OCH_3$), 56.76 (NCH_2CHCOO), 57.19 (NCH_2CH_2O), 60.37 (NCH_2CH_2O), 86.19 (NCH_2CH_2OC), 113.00 ($CCHCHCOCH_3$), 127.65 (CCHCHCCOO), 128.66 (CCHCHCCOO), 129.65 (d, $CCHCHCOCH_3$), 134.88 (CCHCHCCOO), 135.60 (d, $CCHCHCOCH_3$), 147.10 (CCHCHCCOO), 157.89 ($CCHCHCOCH_3$), 174.28 ($C_{Ar}COO$), 182.68 (CHCOO). IR (KBr): ṽ = 2935, 1718, 1607, 1543, 1509, 1396, 1301, 1251, 1176, 1089, 1034, 828, 796 cm^{-1}. HRMS-ESI+ *m/z* $[M+H]^+$ calcd for $C_{30}H_{33}NO_7$: 520.2330, found: 520.2331.

***rac*-1-(2-{[4-(Methoxymethyl)phenyl]bis[4-methoxyphenyl]methoxy}ethyl)piperidine-3-carboxylic acid (5f):** ^1H NMR (400 MHz, MeOD + NaOD) δ = 1.25 – 1.40 (m, 1H, $NCH_2CH_2CH_{ax}H_{eq}$), 1.53 (qt, *J*=13.2, 3.9, 1H, $NCH_2CH_{ax}H_{eq}CH_2$), 1.61 – 1.68 (m, 1H,

$NCH_2CH_{ax}H_{eq}CH_2$), 1.89-2.02 (m, 2H, $NCH_2CH_2CH_{ax}H_{eq}$ + $NCH_{ax}H_{eq}CH_2CH_2$), 2.07 (t, *J*=11.4, 1H, $NCH_{ax}H_{eq}CHCOO$), 2.34 (tt, *J*=11.8, 3.7, 1H, NCH_2CHCOO), 2.63 (t, *J*=6.4, 2H, NCH_2CH_2O), 2.83 (br d, *J*=11.4, 1H, $NCH_{ax}H_{eq}CH_2CH_2$), 3.01 – 3.08 (m, 1H, $NCH_{ax}H_{eq}CHCOO$), 3.20 – 3.30 (m, 2H, NCH_2CH_2O), 3.35 (s, 3H, CH_2OCH_3), 3.77 (s, 6H, $C_{Ar}OCH_3$), 4.42 (s, 2H, CH_2OCH_3), 6.79 – 6.88 (m, 4H, $CCHCHCOCH_3$), 7.23 – 7.33 (m, 6H, $CCHCHCOCH_3$ + $CCHCHCCH_2OCH_3$), 7.38 – 7.45 (m, 2H, $CCHCHCCH_2OCH_3$). ^{13}C NMR (101 MHz, MeOD + NaOD) δ = 25.95 ($NCH_2CH_2CH_2$), 29.32 ($NCH_2CH_2CH_2$), 46.35 (NCH_2CHCOO), 55.47 ($NCH_2CH_2CH_2$), 55.77 ($C_{Ar}OCH_3$), 58.35 (CH_2OCH_3), 58.82 (NCH_2CHCOO), 59.56 (NCH_2CH_2O), 62.38 (NCH_2CH_2O), 75.35 (CH_2OCH_3), 87.55 (NCH_2CH_2OC), 114.11 ($CCHCHCOCH_3$), 128.40 ($CCHCHCCH_2OCH_3$), 129.32 ($CCHCHCCH_2OCH_3$), 131.22 ($CCHCHCOCH_3$), 137.44 ($CCHCHCOCH_3$), 137.80 ($CCHCHCCH_2OCH_3$), 146.32 ($CCHCHCCH_2OCH_3$), 160.10 ($CCHCHCOCH_3$), 182.82 (*C*OO). IR (KBr): ṽ = 2933, 1608, 1508, 1457, 1405, 1302, 1250, 1176, 1153, 1091, 1033, 916, 826, 582 cm^{-1}. HRMS-ESI- *m/z* $[M\text{-}H]^-$ calcd for $C_{31}H_{37}NO_6$: 518.2548, found: 518.2556.

***rac*-1-{2-[(4-Cyanophenyl)bis(4-methoxyphenyl)methoxy]ethyl}piperidine-3-carboxylic acid (5g):** ^1H NMR (500 MHz, MeOD + NaOD) δ = 1.24 – 1.40 (m, 1H, $NCH_2CH_2CH_{ax}H_{eq}$), 1.55 (qt, *J*=12.9, 3.8, 1H, $NCH_2CH_{ax}H_{eq}CH_2$), 1.62 – 1.70 (m, 1H, $NCH_2CH_{ax}H_{eq}CH_2$), 1.97 (td, *J*=11.9, 3.0, 2H, $NCH_2CH_2CH_{ax}H_{eq}$ + $NCH_{ax}H_{eq}CH_2CH_2$), 2.08 (t, *J*=11.4, 1H, $NCH_{ax}H_{eq}CHCOO$), 2.35 (tt, *J*=11.8, 3.8, 1H, NCH_2CHCOO), 2.64 (t, *J*=6.2, 2H, NCH_2CH_2O), 2.81 (d, *J*=11.1, 1H, $NCH_{ax}H_{eq}CH_2CH_2$), 3.01 – 3.09 (m, 1H, $NCH_{ax}H_{eq}CHCOO$), 3.19 – 3.29 (m, 2H, NCH_2CH_2O), 3.79 (s, 6H, $C_{Ar}OCH_3$), 6.83 – 6.96 (m, 4H, $CCHCHCOCH_3$), 7.23 – 7.36 (m, 4H, $CCHCHCOCH_3$), 7.61 – 7.71 (m, 4H, CCHCHCCN + CCHCHCCN). ^{13}C NMR (126 MHz, MeOD + NaOD) δ = 25.98 ($NCH_2CH_2CH_2$), 29.33 ($NCH_2CH_2CH_2$), 46.39 (NCH_2CHCOO), 55.49 ($NCH_2CH_2CH_2$), 55.79 ($C_{Ar}OCH_3$), 58.78 (NCH_2CHCOO), 59.42 (NCH_2CH_2O), 62.54 (NCH_2CH_2O), 87.45 (NCH_2CH_2OC), 111.14 (CCHCHCCN), 114.42 ($CCHCHCOCH_3$), 119.83 ($C_{Ar}CN$), 129.62 (CCHCHCCN), 131.37 ($CCHCHCOCH_3$), 132.79 (CCHCHCCN), 135.75 ($CCHCHCOCH_3$), 153.50 (CCHCHCCN), 160.52 ($CCHCHCOCH_3$), 182.79 (*C*OO). IR (KBr): ṽ = 2928, 2362, 2343, 1718, 1608, 1508 1458, 1395, 1300, 1251, 1176, 1032, 826, 668 cm^{-1}. HRMS-ESI+ *m/z* $[M+H]^+$ calcd for $C_{30}H_{32}N_2O_5$: 501.2384, found: 501.2386.

***rac*-1-(2-{Bis[4-(methoxymethyl)phenyl][4-methoxyphenyl]methoxy}ethyl)piperidine-3-carboxylic acid (5h):**
^1H NMR (500 MHz, MeOD + NaOD) δ = 1.25 – 1.40 (m, 1H, $NCH_2CH_2CH_{ax}H_{eq}$), 1.53 (qt, *J*=13.2, 4.0, 1H, $NCH_2CH_{ax}H_{eq}CH_2$), 1.65 (dt, *J*=13.4, 2.6, 1H, $NCH_2CH_{ax}H_{eq}CH_2$), 1.89-2.02 (m, 2H, $NCH_2CH_2CH_{ax}H_{eq}$ + $NCH_{ax}H_{eq}CH_2CH_2$), 2.07 (t, *J*=11.4, 1H, $NCH_{ax}H_{eq}CHCOO$), 2.34 (tt, *J*=11.8, 3.7, 1H, NCH_2CHCOO), 2.59 – 2.68 (m, 2H, NCH_2CH_2O), 2.82 (br d, *J*=11.4, 1H, $NCH_{ax}H_{eq}CH_2CH_2$), 2.99 – 3.07 (m, 1H, $NCH_{ax}H_{eq}CHCOO$), 3.19 – 3.30 (m, 2H, NCH_2CH_2O), 3.35 (s, 6H, CH_2OCH_3), 3.78 (s, 3H, $C_{Ar}OCH_3$), 4.43 (s, 4H, CH_2OCH_3), 6.83 – 6.89 (m, 2H, $CCHCHCOCH_3$), 7.25 – 7.31 (m, 6H, $CCHCHCOCH_3$ + $CCHCHCCH_2OCH_3$), 7.39 – 7.44 (m, 4H, $CCHCHCCH_2OCH_3$). ^{13}C NMR (126 MHz, MeOD + NaOD) δ = 25.95 ($NCH_2CH_2CH_2$), 29.31 ($NCH_2CH_2CH_2$), 46.34 (NCH_2CHCOO), 55.45 ($NCH_2CH_2CH_2$), 55.82 ($C_{Ar}OCH_3$), 58.38 (CH_2OCH_3), 58.83 (NCH_2CHCOO), 59.52 (NCH_2CH_2O), 62.49 (NCH_2CH_2O), 75.31 (CH_2OCH_3), 87.74 (NCH_2CH_2OC), 114.17 ($CCHCHCOCH_3$), 128.44 ($CCHCHCCH_2OCH_3$), 129.56 ($CCHCHCCH_2OCH_3$), 131.55 ($CCHCHCOCH_3$), 136.76 ($CCHCHCOCH_3$), 138.06 ($CCHCHCCH_2OCH_3$), 145.66 ($CCHCHCCH_2OCH_3$), 160.26 ($CCHCHCOCH_3$), 182.85 (*C*OO). IR (KBr): ṽ = 2930, 1609, 1508, 1458, 1405, 1296, 1250, 1176, 1036, 827 cm^{-1}. HRMS-ESI+ *m/z* $[M+H]^+$ calcd for $C_{32}H_{39}NO_6$: 534.2850, found: 534.2848.

***rac*-1-(2-{Tris[4-(methoxymethyl)phenyl]methoxy}ethyl)piperidine-3-carboxylic acid (5i):** ^{1}H NMR (500 MHz, MeOD + NaOD) δ = 1.24 – 1.37 (m, 1H, $NCH_2CH_2CH_{ax}H_{eq}$), 1.53 (qt, *J*=13.0, 3.8, 1H, $NCH_2CH_{ax}H_{eq}CH_2$), 1.61 – 1.70 (m, 1H, $NCH_2CH_{ax}H_{eq}CH_2$), 1.89-2.01 (m, 2H, $NCH_2CH_2CH_{ax}H_{eq}$ + $NCH_{ax}H_{eq}CH_2CH_2$), 2.07 (t, *J*=11.4, 1H, $NCH_{ax}H_{eq}CHCOO$), 2.34 (tt, *J*=11.8, 3.7, 1H, NCH_2CHCOO), 2.59 – 2.71 (m, 2H, NCH_2CH_2O), 2.82 (br d, *J*=11.4, 1H, $NCH_{ax}H_{eq}CH_2CH_2$), 3.00 - 3.06 (m, 1H, $NCH_{ax}H_{eq}CHCOO$), 3.21 – 3.30 (m, 2H, NCH_2CH_2O), 3.36 (s, 9H, CH_2OCH_3), 4.44 (s, 6H, CH_2OCH_3), 7.24 – 7.32 (m, 6H, $CCHCHCCH_2OCH_3$), 7.38 – 7.46 (m, 6H, $CCHCHCCH_2OCH_3$). ^{13}C NMR (126 MHz, MeOD + NaOD) δ = 25.96 ($NCH_2CH_2CH_2$), 29.32 ($NCH_2CH_2CH_2$), 46.36 (NCH_2CHCOO), 55.44 ($NCH_2CH_2CH_2$), 58.40 (CH_2OCH_3), 58.86 (NCH_2CHCOO), 59.49 (NCH_2CH_2O), 62.65 (NCH_2CH_2O), 75.27 (CH_2OCH_3), 87.91 (NCH_2CH_2OC), 128.45 ($CCHCHCCH_2OCH_3$), 129.84 ($CCHCHCCH_2OCH_3$), 138.35 ($CCHCHCCH_2OCH_3$), 144.99 ($CCHCHCCH_2OCH_3$), 182.81 (*C*OO). IR (KBr): ṽ = 2930, 2361, 2343, 1609, 1508, 1405, 1296, 1250, 1036, 827, 575 cm^{-1}. HRMS-ESI+ *m/z* $[M+H]^+$ calcd for $C_{33}H_{41}NO_6$: 548.3007, found: 548.3001.

***rac*-1-{2-[(4-Carbamoylphenyl)bis(4-methoxyphenyl)methoxy]ethyl}piperidine-3-carboxylic acid (5j):** ^{1}H NMR (500 MHz, MeOD + NaOD) δ = 1.26 – 1.42 (m, 1H, $NCH_2CH_2CH_{ax}H_{eq}$), 1.57 (qt, *J*=12.9, 3.8, 1H, $NCH_2CH_{ax}H_{eq}CH_2$), 1.64 – 1.72 (m, 1H, $NCH_2CH_{ax}H_{eq}CH_2$), 1.94 – 2.04 (m, 2H, $NCH_2CH_2CH_{ax}H_{eq}$ + $NCH_{ax}H_{eq}CH_2CH_2$), 2.10 (t, *J*=11.4, 1H, $NCH_{ax}H_{eq}CHCOO$), 2.38 (tt, *J*=12.0, 3.7, 1H, NCH_2CHCOO), 2.66 (t, *J*=6.3, 2H, NCH_2CH_2O), 2.85 (d, *J*=11.4, 1H, $NCH_{ax}H_{eq}CH_2CH_2$), 3.06 – 3.12 (m, 1H, $NCH_{ax}H_{eq}CHCOO$), 3.24 – 3.31 (m, 2H, NCH_2CH_2O), 3.80 (s, 6H, $C_{Ar}OCH_3$), 6.85 – 6.94 (m, 4H, $CCHCHCOCH_3$), 7.28 – 7.37 (m, 4H, $CCHCHCOCH_3$), 7.55 – 7.62 (m, 2H, $CCHCHCNH_2$), 7.78 – 7.85 (m, 2H, m, 2H, $CCHCHCNH_2$). ^{13}C NMR (126 MHz, MeOD + NaOD) δ = 25.99 ($NCH_2CH_2CH_2$), 29.34 ($NCH_2CH_2CH_2$), 46.42 (NCH_2CHCOO), 55.52 ($NCH_2CH_2CH_2$), 55.76 ($C_{Ar}OCH_3$), 58.80 (NCH_2CHCOO), 59.51 (NCH_2CH_2O), 62.50 (NCH_2CH_2O), 87.53 (NCH_2CH_2OC), 114.24 ($CCHCHCOCH_3$), 128.20 ($CCHCHCC(O)NH_2$), 129.02 ($CCHCHCCNH_2$), 131.40 ($CCHCHCOCH_3$), 133.11 ($CCHCHCCNH_2$), 136.63 ($CCHCHCOCH_3$), 151.35 ($CCHCHCCNH_2$), 160.32 ($CCHCHCOCH_3$), 172.21 (CNH_2), 182.77 (*C*OO). IR (KBr): ṽ = 2934, 2360, 1670, 1609, 1567, 1508, 1464, 1409, 1302, 1250, 1175, 1070, 1034, 829, 770, 583 cm^{-1}. HRMS-ESI- *m/z* $[M-H]^-$ calcd for $C_{30}H_{34}N_2O_6$: 517.2344, found: 517.2350.

***rac*-1-{2-[Carboxybis(4-methoxyphenyl)methoxy]ethyl}piperidine-3-carboxylic acid (5k):** ^{1}H NMR (500 MHz, D_2O + NaOD) δ = 0.95 – 1.05 (m, 1H, $NCH_2CH_2CH_{ax}H_{eq}$), 1.17 – 1.30 (m, 1H, $NCH_2CH_{ax}H_{eq}CH_2$), 1.38 – 1.52 (m, 2H, $NCH_2CH_{ax}H_{eq}CH_2$ + $NCH_{ax}H_{eq}CH_2CH_2$), 1.61 (t, *J*=11.4, 1H, $NCH_{ax}H_{eq}CHCOO$), 1.69 (br d, *J*=9.9, 1H, $NCH_2CH_2CH_{ax}H_{eq}$), 2.08 (tt, *J*=12.0, 4.5, 2H, NCH_2CHCOO + NCH_2CH_2O), 2.17 (dt, *J*=11.9, 5.7, 1H, NCH_2CH_2O), 2.38 (br d, *J*=11.1, 1H, $NCH_{ax}H_{eq}CH_2CH_2$), 2.56 – 2.66 (m, 1H, $NCH_{ax}H_{eq}CHCOO$), 2.85 (t, *J*=5.7, 2H, NCH_2CH_2O), 3.58 (d, *J*=7.2, 6H, $C_{Ar}OCH_3$), 6.67 – 6.75 (m, 4H, $CCHCHCOCH_3$), 7.12 (dq, *J*=8.5, 3.2, 4H, $CCHCHCOCH_3$). ^{13}C NMR (101 MHz, CH_2Cl_2) δ = 22.50 ($NCH_2CH_2CH_2$), 26.87 ($NCH_2CH_2CH_2$), 40.84 (d, *J*=12.7, NCH_2CHCOO), 53.54 ($NCH_2CH_2CH_2$), 55.70 ($C_{Ar}OCH_3$), 56.38 (NCH_2CHCOO), 58.10 (NCH_2CH_2O), 61.08 (NCH_2CH_2O), 90.11 (NCH_2CH_2OC), 112.70 (d, *J*=1.4, $CCHCHCOCH_3$), 132.19 ($CCHCHCOCH_3$), 133.91 (d, *J*=7.2, $CCHCHCOCH_3$), 158.91 ($CCHCHCOCH_3$), 176.67 (COO). IR (KBr): ṽ = 2956, 1588, 1508, 1252, 1176, 1030, 829, 810, 780, 601, 567 cm^{-1}. HRMS-ESI- *m/z* $[M-H]^-$ calcd for $C_{24}H_{29}O_7N$: 443.1871, found: 442.1878.

***rac*-1-[4-Hydroxy-4,4-bis(4-methoxyphenyl)butyl]piperidine-3-carboxylic acid (5l):** ^{1}H NMR (400 MHz, MeOD + NaOD) δ = 1.26 – 1.40 (m, 1H, $NCH_2CH_2CH_{ax}H_{eq}CH$), 1.47 – 1.69 (m, 4H, $NCH_2CH_{ax}H_{eq}CH_2CH$ + $NCH_2CH_2CH_2COH$ + $NCH_2CH_{ax}H_{eq}CH_2CH$), 1.80 (td, *J*=11.6, 3.2, 1H,

$NCH_{ax}H_{eq}CH_2CH_2CH$), 1.89 – 2.02 (m, 2H, $NCH_2CH_2CH_{ax}H_{eq}CH$ + $NCH_{ax}H_{eq}CHCOO$), 2.24 – 2.43 (m, 5H, $NCH_2CH_2CH_2COH$ + $NCH_2CH_2CH_2COH$ + NCH_2CHCOO), 2.70 (br d, *J*=11.2, 1H, $NCH_{ax}H_{eq}CH_2CH_2CH$), 2.94 – 3.01 (m, 1H, $NCH_{ax}H_{eq}CHCOO$), 3.75 (d, *J*=3.5, 6H, $C_{Ar}OCH_3$), 6.78 – 6.86 (m, 4H, $CCHCHCOCH_3$), 7.25 – 7.33 (m, 4H, $CCHCHCOCH_3$). ^{13}C NMR (126 MHz, Methanol-d_4) δ = 22.59 ($NCH_2CH_2CH_2COH$), 25.91 ($NCH_2CH_2CH_2CH$), 29.54 ($NCH_2CH_2CH_2CH$), 42.58 ($NCH_2CH_2CH_2COH$), 46.21 (NCH_2CHCOO), 54.64 ($NCH_2CH_2CH_2CH$), 55.82 ($C_{Ar}OCH_3$), 58.12 (NCH_2CHCOO), 60.63 ($NCH_2CH_2CH_2COH$), 78.12 (*C*OH), 114.20 (d, $CCHCHCOCH_3$), 128.56 (d, $CCHCHCOCH_3$), 141.57 (d, $CCHCHCOCH_3$), 159.54 (d, $CCHCHCOCH_3$), 182.92 (*C*OO). IR (KBr): ṽ = 2937, 2834, 1608, 1582, 1508, 1463, 1403, 1301, 1247, 1175, 1092, 1033, 829, 781, 668, 635, 592, 574 cm^{-1}. HRMS-ESI- *m/z* [*M*-H]$^-$ calcd for $C_{24}H_{31}NO_5$: 412.2129, found: 412.2137.

***rac*-1-(2-{[4-(Hydroxymethyl)phenyl]bis[4-methoxyphenyl]methoxy}ethyl)piperidine-3-carboxylic acid (5m):** ^1H NMR (500 MHz, MeOD + NaOD) δ = 1.32 (td, *J*=12.7, 4.1, 1H, $NCH_2CH_2CH_{ax}H_{eq}$), 1.54 (qt, *J*=13.0, 3.9, 1H, $NCH_2CH_{ax}H_{eq}CH_2$), 1.64 (dt, *J*=13.4, 3.4, 1H, $NCH_2CH_{ax}H_{eq}CH_2$), 1.93 – 2.00 (m, 2H, $NCH_2CH_2CH_{ax}H_{eq}$ + $NCH_{ax}H_{eq}CH_2CH_2$), 2.07 (t, *J*=11.4, 1H, $NCH_{ax}H_{eq}CHCOO$), 2.34 (tt, *J*=11.9, 3.8, 1H, NCH_2CHCOO), 2.63 (t, *J*=6.4, 2H, NCH_2CH_2O), 2.83 (br d, *J*=11.4, 1H, $NCH_{ax}H_{eq}CH_2CH_2$), 3.00 – 3.08 (m, 1H, $NCH_{ax}H_{eq}CHCOO$), 3.26 (qt, *J*=9.5, 6.5, 2H, NCH_2CH_2O), 3.77 (s, 6H, $C_{Ar}OCH_3$), 4.57 (s, 2H, CH_2OH), 6.80 – 6.87 (m, 4H, $CCHCHCOCH_3$), 7.24 – 7.33 (m, 6H, $CCHCHCOCH_3$ + $CCHCHCCH_2OH$), 7.38 – 7.44 (m, 2H, $CCHCHCCH_2OH$). ^{13}C NMR (126 MHz, MeOD + NaOD) δ = 25.94 ($NCH_2CH_2CH_2$), 29.31 ($NCH_2CH_2CH_2$), 46.35 (NCH_2CHCOO), 55.45 ($NCH_2CH_2CH_2$), 55.73 ($C_{Ar}OCH_3$), 58.78 (NCH_2CHCOO), 59.55 (NCH_2CH_2O), 62.32 (NCH_2CH_2O), 64.90 (CH_2OH), 87.54 (NCH_2CH_2OC), 114.04 ($CCHCHCOCH_3$), 127.48 ($CCHCHCCH_2OH$), 129.32 ($CCHCHCCH_2OH$), 131.18 ($CCHCHCOCH_3$), 137.57 ($CCHCHCOCH_3$), 141.26 ($CCHCHCCH_2OH$), 145.61 ($CCHCHCCH_2OH$), 160.05 ($CCHCHCOCH_3$), 182.77 (*C*OO). IR (KBr): ṽ = 3332, 2935, 2836, 1733, 1607, 1581, 1508, 1463, 1441, 1411, 1301, 1250, 1176, 1153, 1115, 1068, 1034, 916, 828, 735, 701 cm^{-1}. HRMS-ESI- *m/z* [*M*-H]$^-$ calcd for $C_{30}H_{35}NO_6$: 504.2392, found: 504.2396.

***rac*-1-{2-[(4-{[(Carboxymethyl)amino]methyl}phenyl)bis(4-methoxyphenyl)methoxy]ethyl}piperidine-3-carboxylic acid (5n):** ^1H NMR (400 MHz, MeOD + NaOD) δ = 1.23 – 1.39 (m, 1H, $NCH_2CH_2CH_{ax}H_{eq}$), 1.46 – 1.61 (m, 1H, $NCH_2CH_{ax}H_{eq}CH_2$), 1.61 – 1.69 (m, 1H, $NCH_2CH_{ax}H_{eq}CH_2$), 1.91 – 2.02 (m, 2H, $NCH_2CH_2CH_{ax}H_{eq}$ + $NCH_{ax}H_{eq}CH_2CH_2$), 2.07 (t, *J*=11.4, 1H, $NCH_{ax}H_{eq}CHCOO$), 2.35 (tt, *J*=11.8, 3.8, 1H, NCH_2CHCOO), 2.63 (td, *J*=6.5, 2.5, 2H, NCH_2CH_2O), 2.83 (br d, *J*=11.3, 1H, $NCH_{ax}H_{eq}CH_2CH_2$), 3.01 – 3.08 (m, 1H, $NCH_{ax}H_{eq}CHCOO$), 3.15 (s, 2H, CH_2NHCH_2COO), 3.19 – 3.30 (m, 2H, NCH_2CH_2O), 3.70 (s, 2H, CH_2NHCH_2COO), 3.77 (s, 6H, $C_{Ar}OCH_3$), 6.81 – 6.87 (m, 4H, $CCHCHCOCH_3$), 7.26 – 7.32 (m, 6H, $CCHCHCOCH_3$ + $CCHCHCCH_2NH$), 7.37 – 7.42 (m, 2H, $CCHCHCCH_2NH$). ^{13}C NMR (101 MHz, MeOD + NaOD) δ = 25.97 ($NCH_2CH_2CH_2$), 29.35 ($NCH_2CH_2CH_2$), 46.38 (NCH_2CHCOO), 53.43 (CH_2NHCH_2COO), 53.87 (CH_2NHCH_2COO), 55.46 ($NCH_2CH_2CH_2$), 55.71 ($C_{Ar}OCH_3$), 58.89 (NCH_2CHCOO), 59.59 (NCH_2CH_2O), 62.38 (NCH_2CH_2O), 87.52 (NCH_2CH_2OC), 114.05 ($CCHCHCOCH_3$), 128.95 ($CCHCHCCH_2NH$), 129.34 ($CCHCHCCH_2NH$), 131.25 (d, $CCHCHCOCH_3$), 137.49 (d, $CCHCHCOCH_3$), 139.06 ($CCHCHCCH_2NH$), 145.73 ($CCHCHCCH_2NH$), 160.11 ($CCHCHCOCH_3$), 178.84 ($NHCH_2COO$), 182.74 (NCH_2CHCOO). IR (KBr): ṽ = 2933, 1607, 1581, 1508, 1411, 1301, 1250, 1176, 1070, 1035, 827, 582 cm^{-1}. HRMS-ESI- *m/z* [*M*-H]$^-$ calcd for $C_{32}H_{38}N_2O_7$: 561.2606, found: 561.2599.

***rac*-1-{2-[(4-{[(2-Carboxyethyl)amino]methyl}phenyl)bis(4-methoxyphenyl)methoxy]ethyl}piperidine-3-carboxylic acid (5o):** ^1H NMR (500 MHz, MeOD

+ NaOD) δ = 1.24 – 1.37 (m, 1H, $NCH_2CH_2CH_{ax}H_{eq}$), 1.53 (qt, *J*=12.8, 3.8, 1H, $NCH_2CH_{ax}H_{eq}CH_2$), 1.61 – 1.68 (m, 1H, $NCH_2CH_{ax}H_{eq}CH_2$), 1.88 – 2.00 (m, 2H, $NCH_2CH_2CH_{ax}H_{eq}$ + $NCH_{ax}H_{eq}CH_2CH_2$), 2.07 (t, *J*=11.4, 1H, $NCH_{ax}H_{eq}CHCOO$), 2.33 (ddt, *J*=11.9, 7.5, 3.7, 1H, NCH_2CHCOO), 2.39 (t, *J*=6.8, 2H, $NHCH_2CH_2COO$), 2.57 – 2.67 (m, 2H, NCH_2CH_2O), 2.76 – 2.86 (m, 3H $NCH_{ax}H_{eq}CH_2CH_2$ + $NHCH_2CH_2COO$), 2.99 – 3.07 (m, 1H, $NCH_{ax}H_{eq}CHCOO$), 3.19 – 3.30 (m, 2H, NCH_2CH_2O), 3.72 (s, 2H, $C_{Ar}CH_2NH$), 3.77 (s, 6H, $C_{Ar}OCH_3$), 6.81 – 6.86 (m, 4H, $CCHCHCOCH_3$), 7.25 – 7.31 (m, 6H, $CCHCHCOCH_3$ + $CCHCHCCH_2NH$), 7.36 – 7.40 (m, 2H, $CCHCHCCH_2NH$). ^{13}C NMR (101 MHz, MeOD + NaOD) δ = 25.94 ($NCH_2CH_2CH_2$), 29.30 ($NCH_2CH_2CH_2$), 38.19 ($NHCH_2CH_2COO$), 46.32 (NCH_2CHCOO), 47.10 ($NHCH_2CH_2COO$), 54.02 (CH_2NHCH_2COO), 55.45 ($NCH_2CH_2CH_2$), 55.82 ($C_{Ar}OCH_3$), 58.82 (NCH_2CHCOO), 59.57 (NCH_2CH_2O), 62.35 (NCH_2CH_2O), 87.55 (NCH_2CH_2OC), 114.08 ($CCHCHCOCH_3$), 128.92 ($CCHCHCCH_2NH$), 129.36 ($CCHCHCCH_2NH$), 131.22 ($CCHCHCOCH_3$), 137.51 (d, $CCHCHCOCH_3$), 139.19 ($CCHCHCCH_2NH$), 145.54 ($CCHCHCCH_2NH$), 160.07 ($CCHCHCOCH_3$), 181.09 ($NHCH_2COO$), 182.89 (NCH_2CHCOO). IR (KBr): ṽ = 2936, 1560, 1508, 1406, 1302, 1250, 11176, 1068, 1035, 826, 583 cm^{-1}. HRMS-ESI+ *m/z* [*M*+H]$^+$ calcd for $C_{33}H_{40}N_2O_7$: 577.2908, found: 577.2909.

***rac*-1-{2-[(4-{[(1-Carboxycyclopropyl)amino]methyl}phenyl)bis(4-methoxyphenyl)methoxy]ethyl}piperidine-3-carboxylic acid (5p):** ^1H NMR (400 MHz, MeOD + NaOD) δ = 0.80 (q, *J*=3.8, 2H, $NHCCH_{ax}$), 1.11 (q, *J*=3.8, 2H, , $NHCCH_{eq}$), 1.25 – 1.39 (m, 1H, $NCH_2CH_2CH_{ax}H_{eq}$), 1.46 – 1.71 (m, 2H, $NCH_2CH_{ax}H_{eq}CH_2$ + $NCH_2CH_{ax}H_{eq}CH_2$), 1.89 – 2.01 (m, 2H, $NCH_2CH_2CH_{ax}H_{eq}$ + $NCH_{ax}H_{eq}CH_2CH_2$), 2.07 (t, *J*=11.3, 1H, $NCH_{ax}H_{eq}CHCOO$), 2.34 (tt, *J*=11.7, 3.5, 1H, NCH_2CHCOO), 2.58 – 2.67 (m, 2H, NCH_2CH_2O), 2.83 (br d, *J*=11.4, 1H, $NCH_{ax}H_{eq}CH_2CH_2$), 2.99 – 3.07 (m, 1H, $NCH_{ax}H_{eq}CHCOO$), 3.20 – 3.29 (m, 2H, NCH_2CH_2O), 3.77 (s, 6H, $C_{Ar}OCH_3$), 3.78 (s, 2H, $C_{Ar}CH_2NH$), 6.81 – 6.87 (m, 4H, $CCHCHCOCH_3$), 7.25 – 7.33 (m, 6H, $CCHCHCOCH_3$ + $CCHCHCCH_2NH$), 7.35 – 7.39 (m, 2H, $CCHCHCCH_2NH$). ^{13}C NMR (101 MHz, MeOD + NaOD) δ = 15.41 ($NHCCH_2$), 25.94 ($NCH_2CH_2CH_2$), 29.31 ($NCH_2CH_2CH_2$), 43.73 (NHC), 46.33 (NCH_2CHCOO), 52.57 ($C_{Ar}CH_2NH$), 55.43 ($NCH_2CH_2CH_2$), 55.81 ($C_{Ar}OCH_3$), 58.84 (NCH_2CHCOO), 59.57 (NCH_2CH_2O), 62.35 (NCH_2CH_2O), 87.57 (NCH_2CH_2OC), 114.07 ($CCHCHCOCH_3$), 129.14 ($CCHCHCCH_2NH$), 129.33 ($CCHCHCCH_2NH$), 131.22 ($CCHCHCOCH_3$), 137.56 ($CCHCHCOCH_3$), 139.79 ($CCHCHCCH_2NH$), 145.41 ($CCHCHCCH_2NH$), 160.06 ($CCHCHCOCH_3$), 182.03 (CHCOO), 182.87 ($C(CH_2)_2COO$). IR (KBr): ṽ = 2933, 1608. 1557, 1508, 1442, 1406, 1338, 1301, 1250, 1175, 1034, 916, 827, 582, 518 cm^{-1}. HRMS-ESI- *m/z* [*M*-H]$^-$ calcd for $C_{34}H_{40}N_2O_7$: 587.2763, found: 587.2773.

***rac*-1-{2-[(4-{[(2-Carboxypropan-2-yl)amino]methyl}phenyl)bis(4-methoxyphenyl)methoxy]ethyl}piperidine-3-carboxylic acid (5q):** ^1H NMR (500 MHz, MeOD + NaOD) δ = 1.26 – 1.36 (m, 7H, CCH_3 + $NCH_2CH_2CH_{ax}H_{eq}$), 1.53 (qt, *J*=13.0, 3.9, 1H, $NCH_2CH_{ax}H_{eq}CH_2$), 1.60 – 1.68 (m, 1H, $NCH_2CH_{ax}H_{eq}CH_2$), 1.90 – 2.00 (m, 2H, $NCH_2CH_2CH_{ax}H_{eq}$ + $NCH_{ax}H_{eq}CH_2CH_2$), 2.07 (t, *J*=11.4, 1H, $NCH_{ax}H_{eq}CHCOO$), 2.34 (tt, *J*=11.9, 3.7, 1H, , NCH_2CHCOO), 2.57 – 2.68 (m, 2H, NCH_2CH_2O), 2.83 (br d, *J*=11.4, 1H, $NCH_{ax}H_{eq}CH_2CH_2$), 3.00 – 3.06 (m, 1H, $NCH_{ax}H_{eq}CHCOO$), 3.20 – 3.29 (m, 2H, NCH_2CH_2O), 3.57 (s, 2H, $C_{Ar}CH_2NH$), 3.77 (s, 6H, $C_{Ar}OCH_3$), 6.77 – 6.93 (m, 4H, $CCHCHCOCH_3$), 7.26 – 7.33 (m, 6H, $CCHCHCOCH_3$ + $CCHCHCCH_2NH$), 7.36 – 7.41 (m, 2H, $CCHCHCCH_2NH$). ^{13}C NMR (126 MHz, MeOD + NaOD) δ = 25.89 ($NHCCH_3$), 25.91 ($NCH_2CH_2CH_2$), 29.27 ($NCH_2CH_2CH_2$), 46.29 (NCH_2CHCOO), 49.27 ($C_{Ar}CH_2NH$), 55.40 ($NCH_2CH_2CH_2$), 55.79 ($C_{Ar}OCH_3$), 58.81 (NCH_2CHCOO), 59.54 (NCH_2CH_2O), 61.74 ($NHC(CH_3)_2$), 62.32 (NCH_2CH_2O), 87.53 (NCH_2CH_2OC), 114.05 ($CCHCHCOCH_3$), 129.13 ($CCHCHCCH_2NH$), 129.35 ($CCHCHCCH_2NH$), 131.20 ($CCHCHCOCH_3$), 137.48 ($CCHCHCOCH_3$), 160.04 ($CCHCHCOCH_3$), 182.85 (CHCOO), 184.22 ($C(CH_3)_2COO$). IR (KBr): ṽ = 2932, 1608, 1578,

1508, 1464, 1403, 1302, 1250, 1174, 1116, 1069, 1033, 916, 827, 583, 519 cm^{-1}. HRMS-ESI- *m/z* [*M*-H]$^-$ calcd for $C_{34}H_{42}N_2O_7$: 589.2919, found: 589.2926.

***rac*-1-[2-(Bis{4-methoxyphenyl}{4-[(2-oxopyrrolidin-1-yl)methyl]phenyl}methoxy)ethyl]piperidine-3-carboxylic acid (5r):** ^{1}H NMR (400 MHz, MeOD + NaOD) δ = 1.25 – 1.40 (m, 1H, NCH$_2$CH$_2$C*H*$_{ax}$H$_{eq}$CH), 1.46 – 1.60 (m, 1H, NCH$_2$C*H*$_{ax}$H$_{eq}$CH$_2$CH), 1.60 – 1.69 (m, 1H, NCH$_2$CH$_{ax}$*H*$_{eq}$CH$_2$CH), 1.88 – 2.17 (m, 5H, NCH$_2$CH$_2$CH$_{ax}$*H*$_{eq}$CH + NC*H*$_{ax}$H$_{eq}$CHCOO + NC*H*$_{ax}$H$_{eq}$CH$_2$CH$_2$CH + NCH$_2$C*H*$_2$CH$_2$CO), 2.28 – 2.38 (m, 1H, NCH$_2$C*H*COO), 2.42 (t, *J*=8.1, 2H, NCH$_2$CH$_2$C*H*$_2$CO), 2.61 (t, *J*=6.4, 2H, NC*H*$_2$CH$_2$O), 2.81 (br d, *J*=12.5, 1H, NCH$_{ax}$*H*$_{eq}$CH$_2$CH$_2$CH), 2.98 – 3.05 (m, 1H, NCH$_{ax}$*H*$_{eq}$CHCOO), 3.18 – 3.29 (m, 2H, NCH$_2$C*H*$_2$O), 3.32 – 3.35 (m, 2H, NC*H*$_2$CH$_2$CH$_2$CO), 3.77 (s, 6H, C$_{Ar}$OCH$_3$), 4.42 (s, 2H, C$_{Ar}$C*H*$_2$N), 6.80 – 6.87 (m, 4H, CCHC*H*COCH$_3$), 7.14 – 7.20 (m, 2H, CCHC*H*CCH$_2$N), 7.24 – 7.31 (m, 4H, CC*H*CHCOCH$_3$), 7.38 – 7.43 (m, 2H, CC*H*CHCCH$_2$N). ^{13}C NMR (101 MHz, MeOD + NaOD) δ = 18.69 (NCH$_2$*C*H$_2$CH$_2$CO), 25.94 (NCH$_2$*C*H$_2$CH$_2$CH), 29.29 (NCH$_2$CH$_2$*C*H$_2$CH), 31.96 (NCH$_2$CH$_2$*C*H$_2$CO), 46.30 (NCH$_2$*C*HCOO), 47.06 (C$_{Ar}$*C*H$_2$N), 48.31 (N*C*H$_2$CH$_2$CH$_2$CO), 55.46 (N*C*H$_2$CH$_2$CH$_2$), 55.86 (C$_{Ar}$O*C*H$_3$), 58.80 (N*C*H$_2$CHCOO), 59.54 (N*C*H$_2$CH$_2$O), 62.37 (NCH$_2$*C*H$_2$O), 87.50 (NCH$_2$CH$_2$O*C*), 114.16 (CCH*C*HCOCH$_3$), 128.43 (CCH*C*HCCH$_2$NH), 129.60 (C*C*HCHCCH$_2$NH), 131.22 (C*C*HCHCOCH$_3$), 136.12 (CCHCH*C*CH$_2$NH), 137.33 (*C*CHCHCOCH$_3$), 146.29 (*C*CHCHCCH$_2$NH), 160.10 (CCHCH*C*OCH$_3$), 177.68 (N*C*O), 182.91 (*C*OO). IR (KBr): ṽ = 2933, 1670, 1608, 1581, 1508, 1463, 1410, 1300, 1249, 1175, 1115, 1069, 1032, 915, 828, 667, 635, 582, 515 cm^{-1}. HRMS-ESI+ *m/z* [*M*+H]$^+$ calcd for $C_{34}H_{40}NO_6$: 573.2959, found: 573.2957.

***rac*-1-{2-[(4-{[(3-Carboxypropyl)amino]methyl}phenyl)bis(4-methoxyphenyl)methoxy]ethyl}piperidine-3-carboxylic acid (5s):** ^{1}H NMR (400 MHz, MeOD + NaOD) δ = 1.24 – 1.38 (m, 1H, NCH$_2$CH$_2$C*H*$_{ax}$H$_{eq}$), 1.46 – 1.60 (m, 1H, NCH$_2$C*H*$_{ax}$H$_{eq}$CH$_2$), 1.60 – 1.70 (m, 1H, NCH$_2$CH$_{ax}$*H*$_{eq}$CH$_2$), 1.70 – 1.85 (m, 2H, NHCH$_2$C*H*$_2$CH$_2$COO), 1.89 – 2.01 (m, 2H, NCH$_2$CH$_2$CH$_{ax}$*H*$_{eq}$ + NC*H*$_{ax}$H$_{eq}$CH$_2$CH$_2$), 2.07 (t, *J*=11.3, 1H, NC*H*$_{ax}$H$_{eq}$CHCOO), 2.14 – 2.21 (m, 2H, NHCH$_2$CH$_2$C*H*$_2$COO), 2.34 (tt, *J*=11.7, 3.7, 1H, NCH$_2$C*H*COO), 2.57 – 2.67 (m, 4H, NHC*H*$_2$CH$_2$CH$_2$COO + NC*H*$_2$CH$_2$O), 2.83 (br d, *J*=11.3, 1H, NCH$_{ax}$*H*$_{eq}$CH$_2$CH$_2$), 2.99 – 3.07 (m, 1H, NCH$_{ax}$*H*$_{eq}$CHCOO), 3.20 – 3.29 (m, 2H, NCH$_2$C*H*$_2$O), 3.70 (s, 2H, C$_{Ar}$C*H*$_2$NH), 3.77 (s, 6H, C$_{Ar}$OCH$_3$), 6.81 – 6.88 (m, 4H, CCHC*H*COCH$_3$), 7.24 – 7.33 (m, 6H, CC*H*CHCOCH$_3$ + CCHC*H*CCH$_2$NH), 7.36 – 7.41 (m, 2H, CC*H*CHCCH$_2$NH). ^{13}C NMR (101 MHz, MeOD + NaOD) δ = 25.94 (NCH$_2$*C*H$_2$CH$_2$), 27.33 (NHCH$_2$*C*H$_2$CH$_2$COOH), 29.31 (NCH$_2$CH$_2$*C*H$_2$), 36.94 (NHCH$_2$CH$_2$*C*H$_2$COOH), 46.32 (NCH$_2$*C*HCOO), 50.14 (NH*C*H$_2$CH$_2$CH$_2$COOH), 54.05 (C$_{Ar}$*C*H$_2$NH), 55.44 (N*C*H$_2$CH$_2$CH$_2$), 55.81 (C$_{Ar}$O*C*H$_3$), 58.83 (N*C*H$_2$CHCOO), 59.58 (N*C*H$_2$CH$_2$O), 62.35 (NCH$_2$*C*H$_2$O), 87.55 (NCH$_2$CH$_2$O*C*), 114.09 (CCH*C*HCOCH$_3$), 128.93 (d, CCH*C*HCCH$_2$NH), 129.35 (C*C*HCHCCH$_2$NH), 131.23 (C*C*HCHCOCH$_3$), 137.52 (CCHCH*C*CH$_2$NH), 139.23 (*C*CHCHCOCH$_3$), 145.52 (*C*CHCHCCH$_2$NH), 160.07 (CCHCH*C*OCH$_3$), 182.53 (CH$_2$*C*OO), 182.87 (CH*C*OO). IR (KBr): ṽ = 2926, 1628, 1508, 1458, 1250, 1175, 1033, 701, 470 cm^{-1}. HRMS-ESI- *m/z* [*M*-H]$^-$ calcd for $C_{36}H_{42}N_2O_7$: 589.2919, found: 589.2917.

***rac*-1-{2-[(4-{[(4-Carboxyphenyl)amino]methyl}phenyl)bis(4-methoxyphenyl)methoxy]ethyl}piperidine-3-carboxylic acid (5t):** ^{1}H NMR (500 MHz, D$_2$O + NaOD) δ = 1.19 – 1.34 (m, 1H, NCH$_2$CH$_2$C*H*$_{ax}$H$_{eq}$), 1.34 – 1.47 (m, 1H, NCH$_2$C*H*$_{ax}$H$_{eq}$CH$_2$), 1.52 – 1.61 (m, 1H, NCH$_2$CH$_{ax}$*H*$_{eq}$CH$_2$), 1.80 – 1.88 (m, 1H, NC*H*$_{ax}$H$_{eq}$CH$_2$CH$_2$), 1.88 – 1.99 (m, 1H, NCH$_2$CH$_2$CH$_{ax}$*H*$_{eq}$), 2.07-2.23 (m, 1H, NC*H*$_{ax}$H$_{eq}$CHCOO), 2.27 – 2.38 (m, 1H, NCH$_2$C*H*COO), 2.50 – 2.70 (m, 3H, NCH$_{ax}$*H*$_{eq}$CH$_2$CH$_2$ + NC*H*$_2$CH$_2$O), 2.88 (br d, *J*=11.1, 1H, NCH$_{ax}$*H*$_{eq}$CHCOO), 3.15 – 3.27 (m, 2H, NCH$_2$C*H*$_2$O), 3.58 (s, 6H, C$_{Ar}$OCH$_3$), 4.11 (s, 2H, C$_{Ar}$C*H*$_2$NH), 6.52 (br d, *J*=8.7, 2H,

NHCC*H*CHCCOO), 6.67 (br d, *J*=8.3, 4H, CCHC*H*COCH$_3$), 7.09 (br d, *J*=8.0, 2H, CCHC*H*CCCH$_2$NH), 7.22 (br dd, *J*=22.2, 8.2, 6H, CCHC*H*COCH$_3$ + CC*H*CHCCCH$_2$NH), 7.58 – 7.67 (m, 2H, NHCCHC*H*CCOO). ^{13}C NMR (126 MHz, D$_2$O + NaOD) δ = 24.49 (NCH$_2$*C*H$_2$CH$_2$), 27.89 (NCH$_2$CH$_2$*C*H$_2$), 44.94 (NCH$_2$*C*HCOO), 46.88 (C$_{Ar}$*C*H$_2$NH), 53.02 (N*C*H$_2$CH$_2$CH$_2$), 55.21 (C$_{Ar}$O*C*H$_3$), 57.33 (N*C*H$_2$CHCOO), 57.80 (N*C*H$_2$CH$_2$O), 60.93 (NCH$_2$*C*H$_2$O), 86.68 (NCH$_2$CH$_2$O*C*), 112.31 (NHC*C*HCHCCOO), 113.49 (CCH*C*HCOCH$_3$), 125.08 (CCHCH*C*COO), 127.43 (CCH*C*HCCCH$_2$NH), 128.71 (C*C*HCHCCCH$_2$NH), 130.13 (C*C*HCHCOCH$_3$), 131.46 (NHCC*C*HCCOO), 143.67 (*C*CHCHCCH$_2$NH), 136.56 (d, *C*CHCHCOCH$_3$), 138.18 (CCHCH*C*CH$_2$NH), 150.90 (NH*C*CHCHCCOO), 158.30 (CCHCH*C*OCH$_3$), 175.58 (C$_{Ar}$*C*OO) 183.20 (CH*C*OO). IR (KBr): ṽ = 2934, 1606, 1508, 1464, 1385, 1332, 1302, 1250, 1177, 1084, 1032, 917, 827, 788, 703, 583 cm^{-1}. HRMS-ESI- *m/z* [*M*-H]$^-$ calcd for $C_{37}H_{40}N_2O_7$: 623.2763, found: 623.2764.

[4-(Dimethoxymethyl)phenyl]bis(4-methoxyphenyl)methanol (8a): ^{1}H NMR (500 MHz, CD$_2$Cl$_2$) δ = 2.81 (s, 1H, COH), 3.30 (s, 6H, CHOC*H$_3$*), 3.78 (s, 6H, C$_{Ar}$OC*H$_3$)*, 5.34 (s, 1H, C*H*OCH$_3$), 6.78 – 6.88 (m, 4H, CCHC*H*COCH$_3$), 7.11 – 7.20 (m, 4H, CC*H*CHCOCH$_3$), 7.22 – 7.29 (m, 2H, CC*H*CHCCHO), 7.32 – 7.40 (m, 2H, CCHC*H*CCHO). ^{13}C NMR (126 MHz, CD$_2$Cl$_2$) δ = 53.13 (CHO*C*H$_3$), 55.61 (C$_{Ar}$O*C*H$_3$), 81.51 (COH), 103.56 (CCHCHC*C*HO), 113.47 (CCH*C*HCOCH$_3$), 126.53 (C*C*HCHCCHO), 127.89 (CCH*C*HCCHO), 129.41 (C*C*HCHCOCH$_3$), 137.61 (CCHCH*C*CHO), 139.81 (*C*CHCHCOCH$_3$), 148.02 (*C*CHCHCCHO), 159.14 (CCHCH*C*OCH$_3$). IR (KBr): ṽ = 3448, 2935, 2833, 1608, 1508, 1436, 1353, 1299, 1250, 1214, 1176, 1101, 1035, 984, 907, 828, 587 cm^{-1}. HRMS-ESI- *m/z* [*M*-H]$^-$ calcd for $C_{24}H_{26}O_5$: 393.1707, found: 393.1706.

Bis(4-methoxyphenyl)[4-(2-methyl-1,3-dioxan-2-yl)phenyl]methanol (8b): ^{1}H NMR (500 MHz, CD$_2$Cl$_2$) δ = 1.21 – 1.28 (m, 1H, COCH$_2$C*H$_{ax}$*H$_{eq}$CH$_2$), 1.44 (s, 3H, CCH$_3$), 1.97 – 2.09 (m, 1H, COCH$_2$CH$_{ax}$*H$_{eq}$*CH$_2$), 2.79 (s, 1H, OH), 3.72 – 3.85 (m, 10H, COC*H$_2$*CH$_2$CH$_2$ + COCH$_2$CH$_2$C*H$_2$* + C$_{Ar}$OCH$_3$), 6.82 – 6.86 (m, 4H, CCHC*H*COCH$_3$), 7.16 – 7.20 (m, 4H, CC*H*CHCOCH$_3$), 7.26 – 7.30 (m, 2H, CC*H*CHCCCH$_3$), 7.33 – 7.37 (m, 2H, CCHC*H*CCCH$_3$). ^{13}C NMR (126 MHz, CD$_2$Cl$_2$) δ = 26.10 (COCH$_2$*C*H$_2$), 32.61 (C*C*H$_3$), 55.79 (C$_{Ar}$O*C*H$_3$), 61.72 (CO*C*H$_2$CH$_2$), 81.76 (COH), 100.81 (*C*CH$_3$), 113.64 (CCH*C*HCOCH$_3$), 126.89 (CCH*C*HCCCH$_3$), 128.53 (C*C*HCHCCCH$_3$), 129.56 (C*C*HCHCOCH$_3$), 140.06 (*C*CHCHCOCH$_3$), 140.89 (CCHCH*C*CCH$_3$), 147.28 (*C*CHCHCCCH$_3$), 159.32 (CCHCH*C*OCH$_3$). IR (KBr): ṽ = 3463, 2958, 1608, 1509, 1463, 1370, 1298, 1249, 1189, 1145, 1081, 1034, 969, 940, 897, 860, 829, 606, 585 cm^{-1}. HRMS-ESI- *m/z* [*M*-H]$^-$ calcd for $C_{26}H_{28}O_5$: 419.1864, found: 419.1875.

4-[Hydroxybis(4-methoxyphenyl)methyl]-N,N-dimethylbenzenesulfonamide (8c): ^{1}H NMR (400 MHz, THF-*d*$_8$) δ = 2.62 (s, 6H, NSO$_2$CH$_3$), 3.74 (s, 6H, C$_{Ar}$OCH$_3$), 6.75 – 6.84 (m, 4H, CCHC*H*COCH$_3$), 7.10 – 7.18 (m, 4H, CC*H*CHCOCH$_3$), 7.48 – 7.56 (m, 2H, CCHC*H*CS), 7.61 – 7.68 (m, 2H, CC*H*CHCS). ^{13}C NMR (101 MHz, THF-*d*$_8$) δ = 37.04 (NSO$_2$CH$_3$), 54.37 (C$_{Ar}$O*C*H$_3$), 80.09 (COH), 112.70 (CCH*C*HCOCH$_3$), 126.92 (C*C*HCHCS), 128.22 (CCH*C*HCS), 129.02 (C*C*HCHCOCH$_3$), 134.18 (CCHCH*C*S), 139.66 (*C*CHCHCOCH$_3$), 153.42 (*C*CHCHCS), 158.81 (CCHCH*C*OCH$_3$). IR (KBr): ṽ = 3514, 2961, 2834, 1608, 1580, 1508, 1463, 1397, 1329, 1297, 1255, 1171, 1090, 1032, 951, 913, 837, 800, 754, 737, 720, 695, 638, 619, 596, 585, 520, 508, 492 cm^{-1}. HRMS-ESI+ *m/z* [*M*+H]$^+$ calcd for $C_{23}H_{25}NO_5S$: 428.1526, found: 428.1529.

Imidazo[1,2-a]pyridin-6-ylbis(4-methoxyphenyl)methanol (8d): ^{1}H NMR (500 MHz, CD$_2$Cl$_2$) δ = 3.79 (s, 6H, C$_{Ar}$OC*H$_3$*), 3.91 (s, 1H, OH), 6.58 (td, *J*=6.9, 1.2, 1H, CCHNC*H*CHN), 6.79 (s, 1H, CC*H*NCHCHN), 6.83 – 6.88 (m, 4H, CCHC*H*COCH$_3$), 7.09 (ddd, *J*=9.1, 6.7, 1.3, 1H, CC*H*CHCN), 7.18 – 7.24 (m, 4H, CC*H*CHCOCH$_3$), 7.41 (dt, *J*=9.1, 1.2, 1H, CCHC*H*CN), 8.04 (dt, *J*=7.0, 1.2, 1H,

CCHNCHC*H*N). ^{13}C NMR (126 MHz, CD_2Cl_2) δ = 55.80 ($C_{Ar}OCH_3$), 77.25 (COH), 111.97 (CCHNCHCHN), 114.06 (CCHCHCOCH$_3$), 117.86 (CCHCHCN), 125.03 (CCHCHCN), 127.58 (CCHNC*H*CHN), 128.46 (CCHCHCOCH$_3$), 129.16 (CCHNCHCHN), 135.33 (CCHNCHCHN), 136.91 (CCHCHCOCH$_3$), 147.42 (CCHCHCN), 159.69 (CCHCHCOCH$_3$). HRMS-EI+ *m/z* [*M*]$^+$ calcd for $C_{22}H_{20}N_2O_3$ 360.1468, found: 360.1472.

Ethyl 4-[hydroxybis(4-methoxyphenyl)methyl]benzoate (8e): ^{1}H NMR (500 MHz, CD_2Cl_2) δ = 1.36 (t, *J*=7.1, 3H, COOCH$_2$C*H$_3$*), 2.91 (s, 1H, CO*H*), 3.78 (s, 6H, $C_{Ar}OCH_3$), 4.33 (q, *J*=7.1, 2H, COOC*H$_2$*CH$_3$), 6.79 – 6.88 (m, 4H, CCHC*H*COCH$_3$), 7.11 – 7.18 (m, 2H, CC*H*CHCOCH$_3$), 7.36 – 7.42 (m, 2H, CC*H*CHCCOO), 7.91 – 7.97 (m, 2H, CCHC*H*CCOO). ^{13}C NMR (126 MHz, CD_2Cl_2) δ = 14.51 (COOCH$_2$CH$_3$), 55.64 ($C_{Ar}OCH_3$), 61.32 (COOCH$_2$CH$_3$), 81.55 (COH), 113.63 (CCHCHCOCH$_3$), 128.06 (CCHCHCCOO), 129.32 (CCHCHCCOO), 129.45 (CCHCHCOCH$_3$), 129.67 (CCHCHCCOO), 139.29 (CCHCHCOCH$_3$), 152.67 (CCHCHCCOO), 159.31 (CCHCHCOCH$_3$), 166.62 (COOCH$_2$CH$_3$). IR (KBr): ṽ = 3475, 2835, 2361, 2342, 1716, 1608, 1509, 1277, 1250, 1176, 1104, 1032, 828, 765 cm^{-1}. HRMS-EI+ *m/z* [*M*]$^+$ calcd for $C_{24}H_{24}O_5$: 392.1624, found: 392.1655.

[4-(Methoxymethyl)phenyl]bis(4-methoxyphenyl)methanol (8f): ^{1}H NMR (500 MHz, CD_2Cl_2) δ = 2.78 (s, 1H, OH), 3.36 (s, 3H, CH$_2$OC*H$_3$*), 3.78 (s, 6H, $C_{Ar}OCH_3$), 4.42 (s, 2H, C*H$_2$*OCH$_3$), 6.80 – 6.85 (m, 4H, CCHC*H*COCH$_3$), 7.13 – 7.18 (m, 4H, CC*H*CHCOCH$_3$), 7.22 – 7.28 (m, 4H, CC*H*CHCCH$_2$OCH$_3$ + CCHC*H*CCH$_2$OCH$_3$). ^{13}C NMR (126 MHz, CD_2Cl_2) δ = 55.63 ($C_{Ar}OCH_3$), 58.41 (CH$_2$OCH$_3$), 74.61 (CH$_2$OCH$_3$), 81.54 (COH), 113.48 (CCHCHCOCH$_3$), 127.52 (CCHCHCCH$_2$OCH$_3$), 128.08 (CCHCHCCH$_2$OCH$_3$), 129.43 (CCHCHCOCH$_3$), 137.79 (CCHCHCCH$_2$OCH$_3$), 139.90 (CCHCHCOCH$_3$), 147.24 (CCHCHCCH$_2$OCH$_3$), 159.15 (CCHCHCOCH$_3$). IR (KBr): ṽ = 3437, 2342, 1608, 1508, 1299, 1250, 1176, 1033, 828, 585 cm^{-1}. HRMS+ESI- *m/z* [*M*-H]$^-$ calcd for $C_{23}H_{24}O_4$: 363.1602, found: 363.1605.

4-[Hydroxybis(4-methoxyphenyl)methyl]benzonitrile (8g): ^{1}H NMR (500 MHz, CD_2Cl_2) δ = 2.86 (s, 1H, OH), 3.78 (s, 6H, $C_{Ar}OCH_3$), 6.81 – 6.88 (m, 4H, CCHC*H*COCH$_3$), 7.10 – 7.16 (m, 4H, CC*H*CHCOCH$_3$), 7.43 – 7.47 (m, 2H, CC*H*CHCCN), 7.57 – 7.63 (m, 2H, CCHC*H*CCN). ^{13}C NMR (126 MHz, CD_2Cl_2) δ = 55.42 ($C_{Ar}OCH_3$), 81.45 (COH), 111.20 (CCHCHCCN), 113.79 (CCHCHCOCH$_3$), 119.20 (CCHCHCCN), 128.79 (CCHCHCCN), 129.43 (CCHCHCOCH$_3$), 132.09 (CCHCHCCN), 138.74 (CCHCHCOCH$_3$), 152.93 (CCHCHCCN), 159.49 (CCHCHCOCH$_3$). IR (KBr): ṽ = 3461, 2835, 2227, 1607, 1582, 1509, 1462, 1298, 1250, 1175, 1152, 1114, 1032, 905, 828, 732, 619 cm^{-1}. HRMS+ESI- *m/z* [*M*-H]$^-$ calcd for $C_{22}H_{19}NO_3$: 344.1292, found: 344.1295.

Bis[4-(methoxymethyl)phenyl](4-methoxyphenyl)methanol (8h): ^{1}H NMR (500 MHz, CD_2Cl_2) δ = 2.82 (s, 1H, OH), 3.36 (s, 6H, CH$_2$OC*H$_3$*), 3.78 (s, 3H, $C_{Ar}OCH_3$), 4.42 (s, 4H, C*H$_2$*OCH$_3$), 6.80 – 6.85 (m, 2H, CCHC*H*COCH$_3$), 7.13 – 7.18 (m, 2H, CC*H*CHCOCH$_3$), 7.22 – 7.28 (m, 8H, CC*H*CHCCH$_2$OCH$_3$ + CCHC*H*CCH$_2$OCH$_3$). ^{13}C NMR (126 MHz, CD_2Cl_2) δ = 55.64 ($C_{Ar}OCH_3$), 58.43 (CH$_2$OCH$_3$), 74.60 (CH$_2$OCH$_3$), 81.73 (COH), 113.54 (CCHCHCOCH$_3$), 127.56 (CCHCHCCH$_2$OCH$_3$), 128.14 (CCHCHCCH$_2$OCH$_3$), 129.51 (CCHCHCOCH$_3$), 137.93 (CCHCHCCH$_2$OCH$_3$), 139.63 (CCHCHCOCH$_3$), 146.95 (CCHCHCCH$_2$OCH$_3$), 159.23 (CCHCHCOCH$_3$). IR (KBr): ṽ = 3447, 2929, 2834, 1608, 1508, 1299, 1250, 1179, 1033, 828, 581 cm^{-1}. HRMS-EI+ *m/z* [*M*]$^+$ calcd for $C_{24}H_{26}O_4$: 378.1831, found: 378.1825.

Tris[4-(methoxymethyl)phenyl]methanol (8i): ^{1}H NMR (400 MHz, CD_2Cl_2) δ = 2.86 (s, 1H, OH), 3.36 (s, 9H, CH$_2$OC*H$_3$*), 4.42 (s, 6H, C*H$_2$*OCH$_3$), 7.19 – 7.31 (m, 12H, CC*H*CHCCH$_2$OCH$_3$ + CCHC*H*CCH$_2$OCH$_3$). ^{13}C NMR (101 MHz, CD_2Cl_2) δ = 58.56 ($C_{Ar}OCH_3$), 74.73 (CH$_2$OCH$_3$), 82.05

(COH), 127.75 (CCH*C*HCCH$_2$OCH$_3$), 128.37 (C*C*HCHCCH$_2$OCH$_3$), 138.14 (CCHCH*C*CH$_2$OCH$_3$), 146.87 (*C*CHCHCCH$_2$OCH$_3$). IR (KBr): ṽ = 2929, 2834, 1717, 1700, 1654, 1608, 1582, 1508, 1458, 1412, 1379, 1300, 1250, 1212, 1179, 1155, 1096, 1033, 967, 907, 828, 730, 638, 581, 546 cm^{-1}. HRMS-EI+ *m/z* [*M*]$^+$ calcd for $C_{25}H_{28}O_4$: 392.1988, found: 392.1985.

4-[Hydroxybis(4-methoxyphenyl)methyl]benzamide (8j): ^{1}H NMR (400 MHz, tetrachloroethane-d_2, 80°C) δ = 2.74 (s, 1H, COH), 3.84 (s, 6H, C$_{Ar}$OC*H$_3$*), 5.75 (br s, 2H, CN*H$_2$*), 6.84 – 6.92 (m, 4H, CCHC*H*COCH$_3$), 7.17 – 7.24 (m, 4H, CC*H*CHCOCH$_3$), 7.42 – 7.49 (m, 2H, CC*H*CHCCNH$_2$), 7.73 – 7.79 (m, 2H, CCHC*H*CCNH$_2$). ^{13}C NMR (101 MHz, tetrachloroethane-d_2, 80°C) δ = 55.23 (C$_{Ar}$O*C*H$_3$), 81.11 (COH), 113.45 (CCH*C*HCOCH$_3$), 126.68 (CCH*C*HCCNH$_2$), 127.86 (C*C*HCHCCNH$_2$), 128.94 (C*C*HCHCOCH$_3$), 131.95 (CCHCH*C*CNH$_2$), 138.79 (*C*CHCHCOCH$_3$), 151.25 (C*C*HCHCCNH$_2$), 158.80 (CCHCH*C*OCH$_3$), 168.56 (*C*ONH$_2$). IR (KBr): ṽ = 3451, 3197, 2360, 1650, 1610, 1566, 1509, 1408, 1388, 1296, 1251, 1177, 1030, 916, 831, 774 cm^{-1}. HRMS-EI+ *m/z* [*M*]$^+$ calcd for $C_{17}H_{18}O_5$: 363.1471, found: 363.1468.

Methyl 2-hydroxy-2,2-bis(4-methoxyphenyl)acetate (8k): ^{1}H NMR (500 MHz, $CDCl_3$) δ = 3.81 (s, 6H, C$_{Ar}$OC*H$_3$*), 3.84 (s, 3H, COOC*H$_3$*), 4.08 (s, 1H, OH), 6.82 – 6.92 (m, 4H, CCHC*H*COCH$_3$), 7.30 – 7.37 (m, 4H, CC*H*CHCOCH$_3$). ^{13}C NMR (126 MHz, $CDCl_3$) δ = 53.48 (COO*C*H$_3$), 55.28 (C$_{Ar}$O*C*H$_3$), 80.46 (*C*OH), 113.42 (CCH*C*HCOCH$_3$), 128.61 (C*C*HCHCOCH$_3$), 134.29 (C*C*HCHCOCH$_3$), 159.29 (CCHCH*C*OCH$_3$), 175.38 (*C*OOCH$_3$). IR (KBr): ṽ = 3496, 2361, 1731, 1610, 1510, 1248, 1175, 1160, 1071, 1030, 839, 776 cm^{-1}. HRMS-EI+ *m/z* [*M*]$^+$ calcd for $C_{17}H_{18}O_5$: 302.1154, found: 302.1138.

4-Chloro-1,1-bis(4-methoxyphenyl)butan-1-ol (8l): ^{1}H NMR (400 MHz, CD_2Cl_2) δ = 1.91 (p, *J*=7.2, 2H, ClCH$_2$C*H$_2$*CH$_2$), 2.42 – 2.51 (m, 2H, ClCH$_2$CH$_2$C*H$_2$*), 3.75 (s, 6H, C$_{Ar}$OC*H$_3$*), 3.97 (t, *J*=7.1, 2H, ClC*H$_2$*CH$_2$CH$_2$), 6.77 – 6.86 (m, 4H, CCHC*H*COCH$_3$), 7.24 – 7.33 (m, 4H, CC*H*CHCOCH$_3$). ^{13}C NMR (101 MHz, CD_2Cl_2) δ = 26.04 (ClCH$_2$*C*H$_2$CH$_2$), 39.16 (ClCH$_2$CH$_2$*C*H$_2$), 55.73 (C$_{Ar}$O*C*H$_3$), 67.71 (Cl*C*H$_2$CH$_2$CH$_2$), 87.89 (COH), 113.84 (CCH*C*HCOCH$_3$), 127.44 (C*C*HCHCOCH$_3$), 139.60 (*C*CHCHCOCH$_3$), 158.86 (CCHCH*C*OCH$_3$). IR (KBr): ṽ = 3444, 2930, 2360, 1607, 1508, 1462, 1301, 1246, 1180, 1028, 988, 829, 740, 597, 574 cm^{-1}. HRMS-EI+ *m/z* [*M-HCl*]$^+$ calcd for $C_{18}H_{21}ClO_3$: 284.1407, found: 284.1407.

4-Bromo-N,N-dimethylbenzenesulfonamide (9c): ^{1}H NMR (400 MHz, CD_2Cl_2) δ = 2.68 (s, 6H, NSO$_2$CH$_3$), 7.58 – 7.65 (m, 2H, CCHC*H*CS), 7.69 – 7.76 (m, 2H, CC*H*CHCS). ^{13}C NMR (101 MHz, CD_2Cl_2) δ = 38.31 (NSO$_2$*C*H$_3$), 128.14 (*C*CHCHCS), 129.80 (CCH*C*HCS), 132.90 (C*C*HCHCS), 135.20 (CCHCH*C*S). IR (KBr): ṽ = 2963, 1574, 1458, 1389, 1343, 1262, 1163, 1085, 1067, 1007, 944, 821, 749, 707, 690, 595, 528, 469 cm^{-1}. HRMS-ESI+ *m/z* [*M*+H]$^+$ calcd for $C_8H_{10}BrNO_2S$: 263.9688, found: 263.9691.

1-{4-[Hydroxybis(4-methoxyphenyl)methyl]phenyl}ethan-1-one (11): ^{1}H NMR (400 MHz, CD_2Cl_2) δ = 2.56 (s, 3H, CCH$_3$), 2.84 (s, 1H, COH), 3.78 (s, 6H, C$_{Ar}$OC*H$_3$*), 6.81 – 6.88 (m, 4H, CCHC*H*COCH$_3$), 7.12 – 7.18 (m, 4H, CC*H*CHCOCH$_3$), 7.39 – 7.46 (m, 2H, CC*H*CHCCCH$_3$), 7.84 – 7.90 (m, 2H, CCHC*H*CCCH$_3$). ^{13}C NMR (101 MHz, CD_2Cl_2) δ = 27.05 (C*C*H$_3$), 55.81 (C$_{Ar}$O*C*H$_3$), 81.71 (COH), 113.82 (CCH*C*HCOCH$_3$), 128.35 (CCH*C*HCCCH$_3$), 128.36 (C*C*HCHCCCH$_3$), 129.59 (C*C*HCHCOCH$_3$), 136.45 (CCHCH*C*CCH$_3$), 139.37 (*C*CHCHCOCH$_3$), 152.99 (*C*CHCHCCCH$_3$), 159.52 (CCHCH*C*OCH$_3$), 198.04 (*C*CH$_3$). IR (KBr): ṽ = 3358, 2830, 1686, 1661, 1606, 1508, 1279, 1248, 1175, 1037, 908, 844, 824, 605, 582 cm^{-1}. HRMS-ESI- *m/z* [*M*-H]$^-$ calcd for $C_{23}H_{22}O_4$: 361.1445, found: 361.1447.

***rac*-Ethyl 1-{2-[(4-formylphenyl)bis(4-methoxyphenyl)methoxy]ethyl}piperidine-3-carboxylate (12a):** ^{1}H NMR (400 MHz, CD_2Cl_2) δ = 1.20 (t, *J*=7.1, 3H, $COOCH_2CH_3$), 1.34 – 1.47 (m, 1H, $NCH_2CH_2CH_{ax}H_{eq}$), 1.48 – 1.61 (m, 1H, $NCH_2CH_{ax}H_{eq}CH_2$), 1.64 – 1.74 (m, 1H, $NCH_2CH_2CH_{ax}H_{eq}$), 1.82 – 1.92 (m, 1H, $NCH_2CH_2CH_{ax}H_{eq}$), 2.05 (td, *J*=11.0, 3.1, 1H, $NCH_{ax}H_{eq}CH_2CH_2$), 2.20 (t, *J*=10.5, 1H, $NCH_{ax}H_{eq}CHCOO$), 2.52 (tt, *J*=10.5, 3.8, 1H, NCH_2CHCOO), 2.56 – 2.62 (m, 2H, NCH_2CH_2O), 2.69 (dt, *J*=10.5, 4.1, 1H, $NCH_{ax}H_{eq}CH_2CH_2$), 2.96 (br dd, *J*=11.1, 3.6, 1H, $NCH_{ax}H_{eq}CHCOO$), 3.06 – 3.19 (m, 2H, NCH_2CH_2O), 3.78 (s, 6H, $C_{Ar}OCH_3$), 4.04 – 4.10 (m, 2H, $COOCH_2CH_3$), 6.80 – 6.89 (m, 4H, $CCHCHCOCH_3$), 7.28 – 7.37 (m, 4H, $CCHCHCOCH_3$), 7.67 – 7.71 (m, 2H, CCHCHCCHO), 7.76 – 7.81 (m, 2H, CCHCHCCHO), 9.96 (s, 1H, CHO). ^{13}C NMR (101 MHz, CD_2Cl_2) δ = 14.59 ($COOCH_2CH_3$), 25.31 ($NCH_2CH_2CH_2$), 27.39 ($NCH_2CH_2CH_2$), 42.66 (NCH_2CHCOO), 54.84 ($NCH_2CH_2CH_2$), 55.77 ($C_{Ar}OCH_3$), 56.87 (NCH_2CHCOO), 58.97 (NCH_2CH_2O), 60.69 ($COOCH_2CH_3$), 62.50 (NCH_2CH_2O), 86.48 (NCH_2CH_2OC), 113.74 ($CCHCHCOCH_3$), 128.87 (CCHCHCCHO), 129.69 (CCHCHCCHO), 130.81 ($CCHCHCOCH_3$), 135.43 (CCHCHCCHO), 135.74 ($CCHCHCOCH_3$), 153.63 (CCHCHCCHO), 159.40 ($CCHCHCOCH_3$), 174.54 (COO), 192.31 (CHO). IR (KBr): ṽ = 2938, 2835, 1730, 1701, 1606, 1509, 1464, 1372, 1303, 1251, 1213, 1176, 1153, 1069, 1033, 823 cm^{-1}. HRMS-EI+ *m/z* [*M*]$^+$ calcd for $C_{32}H_{37}NO_6$: 531.2621, found: 531.2643.

(*S*)-Ethyl 1-{2-[(4-formylphenyl)bis(4-methoxyphenyl)methoxy]ethyl}piperidine-3-carboxylate [(*S*)-12a]: The analytical data are consistent with the racemic compound **12a**. $[\alpha]_D^{22}$ = -0.17 (c=2.26 g/100 ml in EtOH).

(*R*)-Ethyl 1-{2-[(4-formylphenyl)bis(4-methoxyphenyl)methoxy]ethyl}piperidine-3-carboxylate [(*R*)-12a]: The analytical data are consistent with the racemic compound **12a**. $[\alpha]_D^{22}$ = +0.15 (c=1.80 g/100 ml in EtOH).

***rac*-Ethyl 1-{2-[(4-acetylphenyl)bis(4-methoxyphenyl)methoxy]ethyl}piperidine-3-carboxylate (12b):** ^{1}H NMR (400 MHz, CD_2Cl_2) δ = 1.20 (t, *J*=7.1, 3H, $COOCH_2CH_3$), 1.35 – 1.47 (m, 1H, $NCH_2CH_2CH_{ax}H_{eq}$), 1.48 – 1.73 (m, 2H, $NCH_2CH_{ax}H_{eq}CH_2$ + $NCH_2CH_{ax}H_{eq}CH_2$), 1.82 – 1.93 (m, 1H, $NCH_2CH_2CH_{ax}H_{eq}$), 2.05 (td, *J*=10.9, 3.0, 1H, $NCH_{ax}H_{eq}CH_2CH_2$), 2.19 (t, *J*=10.6, 1H, $NCH_{ax}H_{eq}CHCOO$), 2.54 (s, 4H, NCH_2CHCOO + CCH_3), 2.59 (t, *J*=5.8, 2H, NCH_2CH_2O), 2.66 – 2.74 (m, 1H, $NCH_{ax}H_{eq}CH_2CH_2$), 2.91-2.99 (m, 1H, $NCH_{ax}H_{eq}CHCOO$), 3.06 – 3.18 (m, 2H, NCH_2CH_2O), 3.78 (s, 6H, $C_{Ar}OCH_3$), 4.04 – 4.10 (m, 2H, $COOCH_2CH_3$), 6.80 – 6.88 (m, 4H, $CCHCHCOCH_3$), 7.27 – 7.36 (m, 4H, $CCHCHCOCH_3$), 7.57 – 7.64 (m, 2H, $CCHCHCCCH_3$), 7.83 – 7.90 (m, 2H, $CCHCHCCCH_3$). ^{13}C NMR (101 MHz, CD_2Cl_2) δ = 14.43 ($COOCH_2CH_3$), 25.17 ($NCH_2CH_2CH_2$), 26.86 (CCH_3), 27.26 ($NCH_2CH_2CH_2$), 42.52 (NCH_2CHCOO), 54.69 ($NCH_2CH_2CH_2$), 55.62 ($C_{Ar}OCH_3$), 56.72 (NCH_2CHCOO), 58.83 (NCH_2CH_2O), 60.53 ($COOCH_2CH_3$), 62.33 (NCH_2CH_2O), 86.28 (NCH_2CH_2OC), 113.53 ($CCHCHCOCH_3$), 128.20 ($CCHCHCC(O)CH_3$), 128.33 ($CCHCHCCCH_3$), 130.60 ($CCHCHCOCH_3$), 135.87 ($CCHCHCCCH_3$ + $CCHCHCOCH_3$), 151.73 ($CCHCHCCCH_3$), 159.19 ($CCHCHCOCH_3$), 174.41 (COO), 197.84 (CCH_3). IR (KBr): ṽ = 2937, 2360, 1730, 1684, 1607, 1508, 1251, 1176, 1032, 827, 601 cm^{-1}. HRMS-ESI+ *m/z* [*M*+H]$^+$ calcd for $C_{33}H_{39}NO_6$: 546.2850, found: 546.2847.

***rac*-Ethyl 1-(2-{[4-(N,N-dimethylsulfamoyl)phenyl]bis[4-methoxyphenyl]methoxy}ethyl)piperidine-3-carboxylate (12c):** ^{1}H NMR (500 MHz, CD_2Cl_2) δ = 1.20 (t, *J*=7.1, 3H, $COOCH_2CH_3$), 1.36 – 1.47 (m, 1H, $NCH_2CH_2CH_{ax}H_{eq}$), 1.48 – 1.61 (m, 1H, $NCH_2CH_{ax}H_{eq}CH_2$), 1.65 – 1.74 (m, 1H, $NCH_2CH_{ax}H_{eq}CH_2$), 1.82 – 1.96 (m, 1H, $NCH_2CH_2CH_{ax}H_{eq}$),

2.05 (td, *J*=11.0, 3.0, 1H, $NCH_{ax}H_{eq}CH_2CH_2$), 2.20 (t, *J*=10.5, 1H, $NCH_{ax}H_{eq}CHCOO$), 2.52 (tt, *J*=10.5, 3.8, 1H, NCH_2CHCOO), 2.56 – 2.62 (m, 2H, NCH_2CH_2O), 2.64 – 2.72 (m, 7H, SO_2NCH_3 + $NCH_{ax}H_{eq}CH_2CH_2$), 2.95 (dd, *J*=11.2, 3.6, 1H, $NCH_{ax}H_{eq}CHCOO$), 3.06 – 3.16 (m, 2H, NCH_2CH_2O), 3.79 (s, 6H, $C_{Ar}OCH_3$), 4.04 – 4.10 (m, 2H, $COOCH_2CH_3$), 6.80 – 6.96 (m, 4H, $CCHCHCOCH_3$), 7.26 – 7.37 (m, 4H, $CCHCHCOCH_3$), 7.61 – 7.74 (m, 4H, CCHCHCS + CCHCHCS). ^{13}C NMR (125 MHz, CD_2Cl_2) δ = 14.44 ($COOCH_2CH_3$), 25.17 ($NCH_2CH_2CH_2$), 27.24 ($NCH_2CH_2CH_2$), 38.21 (NSO_2CH_3), 42.51 (NCH_2CHCOO), 54.69 ($NCH_2CH_2CH_2$), 55.64 ($C_{Ar}OCH_3$), 56.73 (NCH_2CHCOO), 58.82 (NCH_2CH_2O), 60.55 ($COOCH_2CH_3$), 62.36 (NCH_2CH_2O), 86.15 (NCH_2CH_2OC), 113.59 ($CCHCHCOCH_3$), 127.66 (CCHCHCS), 128.80 (CCHCHCS), 130.67 ($CCHCHCOCH_3$), 134.08 (CCHCHCS), 135.52 ($CCHCHCOCH_3$), 151.73 (CCHCHCS), 159.27 ($CCHCHCOCH_3$), 174.38 (COO). IR (KBr): ṽ = 2935, 2360, 2044, 1729, 1608, 1509, 1464, 1344, 1252, 1164, 1031, 951, 827, 752, 691, 584, 531 cm^{-1}. HRMS-ESI+ *m/z* $[M+H]^+$ calcd for $C_{33}H_{42}N_2O_7S$: 611.2786, found: 611.2786.

***rac*-Ethyl 1-(2-{imidazo[1,2-a]pyridin-6-ylbis(4-methoxyphenyl)methoxy}ethyl)piperidine-3-carboxylate (12d):** ^1H NMR (500 MHz, CD_2Cl_2) δ = 1.19 (t, *J*=7.1, 3H, $COOCH_2CH_3$), 1.37 – 1.49 (m, 1H, $NCH_2CH_2CH_{ax}H_{eq}$), 1.50 – 1.60 (m, 1H, $NCH_2CH_{ax}H_{eq}CH_2$), 1.66-1.74 (m, 1H, $NCH_2CH_{ax}H_{eq}CH_2$), 1.80 – 1.93 (m, 1H, $NCH_2CH_2CH_{ax}H_{eq}$), 2.07 (td, *J*=10.9, 3.0, 1H, $NCH_{ax}H_{eq}CH_2CH_2$), 2.20-2.30 (m, 1H, $NCH_{ax}H_{eq}CHCOO$), 2.53 (tt, *J*=10.4, 3.9, 1H, NCH_2CHCOO), 2.61 (td, *J*=5.7, 2.2, 2H, NCH_2CH_2O), 2.66 – 2.74 (m, 1H, $NCH_{ax}H_{eq}CH_2CH_2$), 2.99 (br dd, *J*=11.2, 3.6, 1H, $NCH_{ax}H_{eq}CHCOO$), 3.19 (qt, *J*=9.5, 5.7, 2H, NCH_2CH_2O), 3.79 (s, 6H, $C_{Ar}OCH_3$), 4.04 – 4.10 (m, 2H, $COOCH_2CH_3$), 6.83 – 6.89 (m, 4H, $CCHCHCOCH_3$), 7.03 (dd, *J*=9.5, 1.8, 1H, CCHCHCN), 7.32 – 7.42 (m, 5H, $CCHCHCOCH_3$ + CCHCHCN), 7.54 (d, *J*=1.2, 1H, CCHNCHCHN), 7.61 (t, *J*=1.0, 1H, CCHNCHCHN), 8.46 (dd, *J*=1.9, 1.0, 1H, CCHNCHCHN). ^{13}C NMR (126 MHz, CD_2Cl_2) δ = 14.42 ($COOCH_2CH_3$), 25.17 ($NCH_2CH_2CH_2$), 27.21 ($NCH_2CH_2CH_2$), 42.48 (NCH_2CHCOO), 54.70 ($NCH_2CH_2CH_2$), 55.64 ($C_{Ar}OCH_3$), 56.82 (NCH_2CHCOO), 58.88 (NCH_2CH_2O), 60.57 ($COOCH_2CH_3$), 62.46 (NCH_2CH_2O), 84.92 (NCH_2CH_2OC), 113.53 (CCHNCHCHN), 113.65 ($CCHCHCOCH_3$), 116.68 (CCHCHCN), 125.21 (CCHNCHCHN), 126.37 (CCHCHCN), 130.47 ($CCHCHCOCH_3$), 130.97 (CCHNCHCHN), 134.05 (CCHNCHCHN), 135.19 ($CCHCHCOCH_3$), 144.82 (CCHCHCN), 159.32 ($CCHCHCOCH_3$), 174.40 (COO). IR (KBr): ṽ = 2935, 1728, 1608, 1508, 1464, 1311, 1249, 1176, 1069, 1032, 923, 828, 808, 671, 619, 584 cm^{-1}. HRMS-ESI+ *m/z* $[M+H]^+$ calcd for $C_{32}H_{37}O_5N_3$: 544.2806, found: 544.2811.

***rac*-Ethyl 1-(2-{[4-(ethoxycarbonyl)phenyl]bis[4-methoxyphenyl]methoxy}ethyl)piperidine-3-carboxylate (12e):** ^1H NMR (500 MHz, CD_2Cl_2) δ = 1.20 (t, *J*=7.1, 3H, $CHCOOCH_2CH_3$), 1.35 (t, *J*=7.1, 3H, $C_{Ar}COOCH_2CH_3$), 1.37 – 1.45 (m, 1H, $NCH_2CH_2CH_{ax}H_{eq}$), 1.48 – 1.58 (m, 1H, $NCH_2CH_{ax}H_{eq}CH_2$), 1.64 – 1.72 (m, 1H, $NCH_2CH_{ax}H_{eq}CH_2$), 1.84 – 1.93 (m, 1H, $NCH_2CH_2CH_{ax}H_{eq}$), 2.04 (td, *J*=11.0, 3.0, 1H, $NCH_{ax}H_{eq}CH_2CH_2$), 2.14 – 2.24 (m, 1H, $NCH_{ax}H_{eq}CHCOO$), 2.51 (tt, *J*=10.5, 3.8, 1H, NCH_2CHCOO), 2.56 – 2.62 (m, 2H, NCH_2CH_2O), 2.66 – 2.73 (m, 1H, $NCH_{ax}H_{eq}CH_2CH_2$), 2.92 – 2.99 (m, 1H, $NCH_{ax}H_{eq}CHCOO$), 3.07 – 3.17 (m, 2H, NCH_2CH_2O), 3.78 (s, 6H, $C_{Ar}OCH_3$), 4.04 – 4.10 (m, 2H, $CHCOOCH_2CH_3$), 4.32 (q, *J*=7.1, 2H, $C_{Ar}COOCH_2CH_3$), 6.79 – 6.88 (m, 4H, $CCHCHCOCH_3$), 7.28 – 7.36 (m, 4H, $CCHCHCCH_2OCH_3$), 7.53 – 7.61 (m, 2H, CCHCHCCOO), 7.89 – 7.97 (m, 2H, CCHCHCCOO). ^{13}C NMR (126 MHz, CD_2Cl_2) δ = 14.43 ($CHCOOCH_2CH_3$), 14.53 ($C_{Ar}COOCH_2CH_3$), 25.16 ($NCH_2CH_2CH_2$), 27.25 ($NCH_2CH_2CH_2$), 42.50 (NCH_2CHCOO), 54.68 ($NCH_2CH_2CH_2$), 55.61 ($C_{Ar}OCH_3$), 56.71 (NCH_2CHCOO), 58.82 (NCH_2CH_2O), 60.53 ($CHCOOCH_2CH_3$), 61.22 ($C_{Ar}COOCH_2CH_3$), 62.31 (NCH_2CH_2O), 86.28 (NCH_2CH_2OC), 113.51 ($CCHCHCOCH_3$), 128.22 (CCHCHCCOO), 129.26 (CCHCHCCOO), 129.32 (CCHCHCCOO), 130.57 ($CCHCHCOCH_3$), 135.94 ($CCHCHCOCH_3$), 151.40 (CCHCHCCOO), 159.15 ($CCHCHCOCH_3$), 166.61 ($C_{Ar}COO$), 174.40 (CHCOO). IR (KBr): ṽ = 2938, 1718, 1608, 1509, 1465, 1275, 1251, 1176, 1103,

1033, 827, 765, 704 cm^{-1}. HRMS-ESI+ *m/z* $[M+H]^+$ calcd for $C_{34}H_{41}NO_7$: 576.2956, found: 576.2962.

***rac*-Ethyl 1-(2-{[4-(methoxymethyl)phenyl]bis[4-methoxyphenyl]methoxy}ethyl)piperidine-3-carboxylate (12f):** ^1H NMR (400 MHz, CD_2Cl_2) δ = 1.20 (t, *J*=7.1, 3H, $COOCH_2CH_3$), 1.31 – 1.46 (m, 1H, $NCH_2CH_2CH_{ax}H_{eq}$), 1.46 – 1.60 (m, 1H, $NCH_2CH_{ax}H_{eq}CH_2$), 1.63 – 1.72 (m, 1H, $NCH_2CH_{ax}H_{eq}CH_2$), 1.82 – 1.93 (m, 1H, $NCH_2CH_2CH_{ax}H_{eq}$), 2.03 (td, *J*=10.9, 3.0, 1H, $NCH_{ax}H_{eq}CH_2CH_2$), 2.18 (t, *J*=10.6, 1H, $NCH_{ax}H_{eq}CHCOO$), 2.51 (tt, *J*=10.5, 3.8, 1H, NCH_2CHCOO), 2.58 (t, *J*=6.0, 2H, NCH_2CH_2O), 2.70 (br dt, *J*=11.4, 3.8, 1H, $NCH_{ax}H_{eq}CH_2CH_2$), 2.91 – 3.04 (m, 1H, $NCH_{ax}H_{eq}CHCOO$), 3.06 – 3.21 (m, 2H, NCH_2CH_2O), 3.35 (s, 3H, CH_2OCH_3), 3.77 (s, 6H, $C_{Ar}OCH_3$), 4.04 – 4.10 (m, 2H, $COOCH_2CH_3$), 4.39 (s, 2H, $C_{Ar}CH_2OCH_3$), 6.76 – 6.89 (m, 4H, $CCHCHCOCH_3$), 7.21 – 7.30 (m, 2H, $CCHCHCCH_2OCH_3$), 7.29 – 7.37 (m, 4H, $CCHCHCOCH_3$), 7.38 – 7.46 (m, 2H, $CCHCHCCH_2OCH_3$). ^{13}C NMR (101 MHz, CD_2Cl_2) δ = 14.43 ($COOCH_2CH_3$), 25.16 ($NCH_2CH_2CH_2$), 27.28 ($NCH_2CH_2CH_2$), 42.51 (NCH_2CHCOO), 54.68 ($NCH_2CH_2CH_2$), 55.60 ($C_{Ar}OCH_3$), 56.71 (NCH_2CHCOO), 58.43 (CH_2OCH_3), 58.89 (NCH_2CH_2O), 60.52 ($COOCH_2CH_3$), 62.18 (NCH_2CH_2O), 74.67 (CH_2OCH_3), 86.22 (NCH_2CH_2OC), 113.37 ($CCHCHCOCH_3$), 127.48 ($CCHCHCCH_2OCH_3$), 128.59 ($CCHCHCCH_2OCH_3$), 130.38 ($CCHCHCOCH_3$), 136.93 ($CCHCHCOCH_3$), 137.37 ($CCHCHCCH_2OCH_3$), 145.12 ($CCHCHCCH_2OCH_3$), 158.94 ($CCHCHCOCH_3$), 174.43 (COO). IR (KBr): ṽ = 2936, 2835, 1730, 1608, 1582, 1509, 1464, 1413, 1373, 1302, 1250, 1177, 1154, 1093, 1034, 968, 915, 827, 732, 582 cm^{-1}. HRMS-ESI+ *m/z* $[M+H]^+$ calcd for $C_{33}H_{41}NO_6$: 548.3007, found: 548.3003.

***rac*-Ethyl 1-{2-[(4-cyanophenyl)bis(4-methoxyphenyl)methoxy]ethyl}piperidine-3-carboxylate (12g):** ^1H NMR (400 MHz, CD_2Cl_2) δ = 1.20 (t, *J*=7.1, 3H, $COOCH_2CH_3$), 1.33 – 1.47 (m, 1H, $NCH_2CH_2CH_{ax}H_{eq}$), 1.48 – 1.60 (m, 1H, $NCH_2CH_{ax}H_{eq}CH_2$), 1.64 – 1.74 (m, 1H, $NCH_2CH_{ax}H_{eq}CH_2$), 1.82 – 1.93 (m, 1H, $NCH_2CH_2CH_{ax}H_{eq}$), 2.05 (td, *J*=11.0, 2.7, 1H, $NCH_{ax}H_{eq}CH_2CH_2$), 2.20 (t, *J*=10.5, 1H, $NCH_{ax}H_{eq}CHCOO$), 2.51 (tt, *J*=10.3, 3.7, 1H, NCH_2CHCOO), 2.58 (td, *J*=5.8, 1.5, 2H, NCH_2CH_2O), 2.68 (td, *J*=8.0, 3.8, 1H, $NCH_{ax}H_{eq}CH_2CH_2$), 2.94 (dd, *J*=10.8, 3.6, 1H, $NCH_{ax}H_{eq}CHCOO$), 3.02 – 3.17 (m, 2H, NCH_2CH_2O), 3.78 (s, 6H, $C_{Ar}OCH_3$), 4.04 – 4.10 (m, 2H, $COOCH_2CH_3$), 6.74 – 6.91 (m, 4H, $CCHCHCOCH_3$), 7.23 – 7.38 (m, 4H, $CCHCHCOCH_3$), 7.51 – 7.61 (m, 2H, CCHCHCCN), 7.62 – 7.68 (m, 2H, CCHCHCCN). ^{13}C NMR (101 MHz, CD_2Cl_2) δ = 14.44 ($COOCH_2CH_3$), 25.15 ($NCH_2CH_2CH_2$), 27.22 ($NCH_2CH_2CH_2$), 42.51 (NCH_2CHCOO), 54.68 ($NCH_2CH_2CH_2$), 55.64 ($C_{Ar}OCH_3$), 56.73 (NCH_2CHCOO), 58.79 (NCH_2CH_2O), 60.55 ($COOCH_2CH_3$), 62.35 (NCH_2CH_2O), 86.12 (NCH_2CH_2OC), 110.67 (CCHCHCCN), 113.64 ($CCHCHCOCH_3$), 119.27 (ArCN), 128.79 (CCHCHCCN), 130.66 ($CCHCHCOCH_3$), 132.08 (CCHCHCCN), 135.22 ($CCHCHCOCH_3$), 152.16 (CCHCHCCN), 159.33 ($CCHCHCOCH_3$), 174.38 (COO). IR (KBr): ṽ = 2938, 2227, 1729, 1607, 1509, 1464, 1303, 1252, 1177, 1153, 1069, 1032, 827 cm^{-1}. HRMS-EI+ *m/z* $[M+H]^+$ calcd for $C_{32}H_{36}N_2O_5$: 529.2697, found: 529.2702.

***rac*-Ethyl 1-(2-{bis[4-(methoxymethyl)phenyl][4-methoxyphenyl]methoxy}ethyl)piperidine-3-carboxylate (12h):** ^1H NMR (400 MHz, CD_2Cl_2) δ = 1.20 (t, *J*=7.1, 3H, $COOCH_2CH_3$), 1.34 – 1.47 (m, 1H, $NCH_2CH_2CH_{ax}H_{eq}$), 1.46 – 1.62 (m, 1H, $NCH_2CH_{ax}H_{eq}CH_2$), 1.64 – 1.74 (m, 1H, $NCH_2CH_{ax}H_{eq}CH_2$), 1.84 – 1.93 (m, 1H, $NCH_2CH_2CH_{ax}H_{eq}$), 1.97 – 2.09 (m, 1H, $NCH_{ax}H_{eq}CH_2CH_2$), 2.19 (t, *J*=10.6, 1H, $NCH_{ax}H_{eq}CHCOO$), 2.45 – 2.55 (m, 1H, NCH_2CHCOO), 2.56 – 2.62 (m, 2H, NCH_2CH_2O), 2.66 – 2.75 (m, 1H, $NCH_{ax}H_{eq}CH_2CH_2$), 2.92 – 3.00 (m, 1H, $NCH_{ax}H_{eq}CHCOO$), 3.08 – 3.19 (m, 2H, NCH_2CH_2O), 3.36 (s, 6H, CH_2OCH_3), 3.78 (s, 3H, $C_{Ar}OCH_3$), 4.04 – 4.10 (m, 2H, $COOCH_2CH_3$), 4.40 (s, 4H, $C_{Ar}CH_2OCH_3$), 6.79 – 6.86 (m, 2H, $CCHCHCOCH_3$), 7.22 – 7.26 (m, 4H, $CCHCHCCH_2OCH_3$), 7.29 – 7.34 (m, 2H, $CCHCHCOCH_3$), 7.39 – 7.45 (m, 4H, $CCHCHCCH_2OCH_3$).

^{13}C NMR (101 MHz, CD$_2$Cl$_2$) δ = 14.43 (COOCH$_2$*CH$_3$*), 25.13 (NCH$_2$*C*H$_2$CH$_2$), 27.25 (NCH$_2$CH$_2$*C*H$_2$), 42.47 (NCH$_2$*C*HCOO), 54.65 (N*C*H$_2$CH$_2$CH$_2$), 55.60 (C$_{Ar}$O*C*H$_3$), 56.70 (N*C*H$_2$CHCOO), 58.43 (CH$_2$O*C*H$_3$), 58.84 (N*C*H$_2$CH$_2$O), 60.53 (COO*C*H$_2$CH$_3$), 62.26 (NCH$_2$*C*H$_2$O), 74.65 (*C*H$_2$OCH$_3$), 86.45 (N*C*H$_2$CH$_2$O*C*), 113.39 (CCH*C*HCOCH$_3$), 127.48 (CCH*C*HCCH$_2$OCH$_3$), 128.80 (C*C*HCHCCH$_2$OCH$_3$), 130.62 (C*C*HCHCOCH$_3$), 136.41 (*C*CHCHCOCH$_3$), 137.54 (CCHCH*C*CH$_2$OCH$_3$), 144.59 (*C*CHCHCCH$_2$OCH$_3$), 159.04 (CCHCH*C*OCH$_3$), 174.41 (COO). IR (KBr): ṽ = 2931, 1729, 1676, 1608, 1509, 1464, 1377, 1301, 1251, 1221, 1180, 1153, 1096, 1033, 967, 917, 812, 668, 639, 586. 542, 530 cm^{-1}. HRMS-ESI+ *m/z* [*M*+H]$^+$ calcd for $C_{34}H_{43}NO_6$: 562.3163, found: 562.3161.

***rac*-Ethyl 1-(2-{tris[4-(methoxymethyl)phenyl]methoxy}ethyl)piperidine-3-carboxylate (12i):** ^{1}H NMR (400 MHz, CD$_2$Cl$_2$) δ = 1.20 (t, *J*=7.1, 3H, COOCH$_2$*CH$_3$*), 1.35 – 1.45 (m, 1H, NCH$_2$CH$_2$C*H$_{ax}$*H$_{eq}$), 1.47 – 1.61 (m, 1H, NCH$_2$C*H$_{ax}$*H$_{eq}$CH$_2$), 1.63 – 1.75 (m, 1H, NCH$_2$CH$_{ax}$*H$_{eq}$*CH$_2$), 1.83 – 1.93 (m, 1H, NCH$_2$CH$_2$CH$_{ax}$*H$_{eq}$*), 1.98 – 2.10 (m, 1H, NC*H$_{ax}$*H$_{eq}$CH$_2$CH$_2$), 2.19 (t, *J*=10.6, 1H, NC*H$_{ax}$*H$_{eq}$CHCOO), 2.44 - 2.63 (m, 3H, NCH$_2$C*H*COO + NC*H$_2$*CH$_2$O), 2.70 (br d, *J*=11.3, 1H, NCH$_{ax}$*H$_{eq}$*CH$_2$CH$_2$), 2.96 (br d, *J*=11.2, 1H, NCH$_{ax}$*H$_{eq}$*CHCOO), 3.06 – 3.20 (m, 2H, NCH$_2$C*H$_2$*O), 3.36 (s, 9H, CH$_2$OC*H$_3$*), 4.04 – 4.10 (m, 2H, COOC*H$_2$*CH$_3$), 4.40 (s, 6H, C$_{Ar}$C*H$_2$*OCH$_3$), 7.22 – 7.29 (m, 6H, CCHC*H*CCH$_2$OCH$_3$), 7.38 – 7.46 (m, 6H, CC*H*CHCCH$_2$OCH$_3$). ^{13}C NMR (101 MHz, CD$_2$Cl$_2$) δ = 14.43 (COOCH$_2$*CH$_3$*), 25.13 (NCH$_2$*C*H$_2$CH$_2$), 27.24 (NCH$_2$CH$_2$*C*H$_2$), 42.47 (NCH$_2$*C*HCOO), 54.64 (N*C*H$_2$CH$_2$CH$_2$), 56.71 (N*C*H$_2$CHCOO), 58.44 (CH$_2$O*C*H$_3$), 58.45 (N*C*H$_2$CH$_2$O), 58.81 (COO*C*H$_2$CH$_3$), 62.40 (NCH$_2$*C*H$_2$O), 74.63 (*C*H$_2$OCH$_3$), 86.65 (N*C*H$_2$CH$_2$O*C*), 127.48 (CCH*C*HCCH$_2$OCH$_3$), 129.02 (C*C*HCHCCH$_2$OCH$_3$), 137.71 (CCHCH*C*CH$_2$OCH$_3$), 144.09 (*C*CHCHCCH$_2$OCH$_3$), 174.39 (COO). IR (KBr): ṽ = 2933, 2820, 1731, 1634, 1508, 1452, 1413, 1377, 1311, 1221, 1190, 1154, 1100, 1022, 969, 918, 797, 668, 519 cm^{-1}. HRMS-ESI+ *m/z* [*M*+H]$^+$ calcd for $C_{35}H_{45}NO_6$: 576.3320, found: 576.3316.

***rac*-Ethyl 1-{2-[(4-carbamoylphenyl)bis(4-methoxyphenyl)methoxy]ethyl}piperidine-3-carboxylate (12j):** ^{1}H NMR (400 MHz, tetrachloroethane-*d*$_2$) δ = 1.22 (t, *J*=7.1, 1H, 3H, COOCH$_2$*CH$_3$*), 1.33 – 1.46 (m, 1H, NCH$_2$CH$_2$C*H$_{ax}$*H$_{eq}$), 1.46 – 1.61 (m, 1H, NCH$_2$C*H$_{ax}$*H$_{eq}$CH$_2$), 1.65 – 1.81 (m, 1H, NCH$_2$CH$_{ax}$*H$_{eq}$*CH$_2$), 1.87 – 1.97 (m, 1H, NCH$_2$CH$_2$CH$_{ax}$*H$_{eq}$*), 1.99 – 2.12 (m, 1H, NC*H$_{ax}$*H$_{eq}$CH$_2$CH$_2$), 2.19 (t, *J*=10.7, 1H, NC*H$_{ax}$*H$_{eq}$CHCOO), 2.53 (tt, *J*=10.6, 3.8, 1H, NCH$_2$C*H*COO), 2.63 (t, *J*=6.0, 2H, NC*H$_2$*CH$_2$O), 2.73 (br d, *J*=11.2, 1H, NCH$_{ax}$*H$_{eq}$*CH$_2$CH$_2$), 2.91 – 3.01 (m, 1H, NCH$_{ax}$*H$_{eq}$*CHCOO), 3.07 – 3.20 (m, 2H, NCH$_2$C*H$_2$*O), 3.81 (s, 6H, C$_{Ar}$OCH$_3$), 4.04 – 4.17 (m, 2H, COOC*H$_2$*CH$_3$), 5.62 (br s, 1H, CONH$_2$), 6.10 (br s, 1H, CONH$_2$), 6.81 – 6.89 (m, 4H, CCHC*H*COCH$_3$), 7.29 – 7.37 (m, 4H, CC*H*CHCOCH$_3$), 7.55 – 7.62 (m, 2H, CC*H*CHCNH$_2$), 7.68 – 7.75 (m, 2H, CCHC*H*CNH$_2$). ^{13}C NMR (101 MHz, tetrachloroethne-*d*$_2$) δ = 14.16 (COOCH$_2$*CH$_3$*), 24.53 (NCH$_2$*C*H$_2$CH$_2$), 26.68 (NCH$_2$CH$_2$*C*H$_2$), 41.81 (NCH$_2$*C*HCOO), 53.99 (N*C*H$_2$CH$_2$CH$_2$), 55.22 (C$_{Ar}$O*C*H$_3$), 55.96 (N*C*H$_2$CHCOO), 58.17 (N*C*H$_2$CH$_2$O), 60.23 (COO*C*H$_2$CH$_3$), 61.72 (NCH$_2$*C*H$_2$O), 85.57 (NCH$_2$CH$_2$O*C*), 113.07 (CCH*C*HCOCH$_3$), 126.75 (CCH*C*HCNH$_2$), 128.00 (C*C*HCHCNH$_2$), 130.08 (C*C*HCHCOCH$_3$), 131.28 (CCHCH*C*CNH$_2$), 135.29 (*C*CHCHCOCH$_3$), 150.01 (*C*CHCHCC(O)NH$_2$), 158.27 (CCHCH*C*OCH$_3$), 168.84 (*C*NH$_2$), 174.14 (COO). IR (KBr): ṽ = 2937, 2835, 1728, 1664, 1609, 1509, 1465, 1383, 1302, 1251, 1176, 1156, 1071, 1033, 829, 767, 582 cm^{-1}. HRMS-ESI+ *m/z* [*M*+H]$^+$ calcd for $C_{32}H_{38}N_2O_6$: 547.2803, found: 547.2796.

***rac*-Ethyl 1-{2-[2-methoxy-1,1-bis(4-methoxyphenyl)-2-oxoethoxy]ethyl}piperidine-3-carboxylate (12k):** ^{1}H NMR (500 MHz, CD$_2$Cl$_2$) δ = 1.20 (t, *J*=7.1, 3H, COOCH$_2$*CH$_3$*), 1.34 – 1.46 (m, 1H, NCH$_2$CH$_2$C*H$_{ax}$*H$_{eq}$), 1.50 (dtt, *J*=13.0, 11.0, 3.9, 1H, NCH$_2$C*H$_{ax}$*H$_{eq}$CH$_2$), 1.65 (dp, *J*=15.1, 3.9, 1H, NCH$_2$CH$_{ax}$*H$_{eq}$*CH$_2$), 1.86 (dq, *J*=12.4, 4.0, 1H, NCH$_2$CH$_2$CH$_{ax}$*H$_{eq}$*), 2.02 (td, *J*=11.0, 3.0, 1H,

NC*H*$_{ax}$H$_{eq}$CH$_2$CH$_2$), 2.15 (br t, *J*=10.6, 1H, NC*H*$_{ax}$H$_{eq}$CHCOO), 2.43 – 2.52 (m, 1H, NCH$_2$C*H*COO), 2.56 (t, *J*=6.1, 2H, NC*H*$_2$CH$_2$O), 2.66 – 2.76 (m, 1H, NCH$_{ax}$*H*$_{eq}$CH$_2$CH$_2$), 2.90 – 2.98 (m, 1H, NCH$_{ax}$*H*$_{eq}$CHCOO), 3.30 (t, *J*=6.1, 2H, NCH$_2$C*H*$_2$O), 3.71 (s, 3H, COOCH$_3$), 3.79 (s, 6H, C$_{Ar}$OCH$_3$), 4.04 – 4.10 (m, 2H, COOC*H*$_2$CH$_3$), 6.79 – 6.87 (m, 4H, CCHC*H*COCH$_3$), 7.26 – 7.35 (m, 4H, CC*H*CHCOCH$_3$). ^{13}C NMR (126 MHz, CD$_2$Cl$_2$) δ = 14.41 (COOCH$_2$C*H*$_3$), 25.12 (NCH$_2$*C*H$_2$CH$_2$), 27.27 (NCH$_2$CH$_2$*C*H$_2$), 42.43 (NCH$_2$*C*HCOO), 52.64 (COO*C*H$_3$), 54.45 (N*C*H$_2$CH$_2$CH$_2$), 55.62 (C$_{Ar}$O*C*H$_3$), 56.33 (N*C*H$_2$CHCOO), 58.67 (N*C*H$_2$CH$_2$O), 60.50 (COO*C*H$_2$CH$_3$), 63.46 (NCH$_2$*C*H$_2$O), 86.51 (*C*COOCH$_3$), 113.46 (CCH*C*HCOCH$_3$), 130.06 (C*C*HCHCOCH$_3$), 133.51 (*C*CHCHCOCH$_3$), 159.66 (CCHCH*C*OCH$_3$), 172.95 (*C*OOCH$_3$), 174.42 (*C*OOCH$_2$CH$_3$). IR (KBr): ṽ = 2950, 1731, 1609, 1510, 1465, 1303, 1252, 1175, 1093, 1033, 831, 807, 781, 597, 570 cm^{-1}. HRMS-ESI+ *m/z* [*M*+H]$^+$ calcd for $C_{27}H_{35}O_7N$: 486.2486, found: 486.2499.

***rac*-Ethyl 1-[4-hydroxy-4,4-bis(4-methoxyphenyl)butyl]piperidine-3-carboxylate (12l):** ^{1}H NMR (400 MHz, CD$_2$Cl$_2$) δ = 1.28 (t, *J*=7.1, 3H, COOCH$_2$C*H*$_3$), 1.34 – 1.48 (m, 1H, NCH$_2$CH$_2$C*H*$_{ax}$H$_{eq}$CH), 1.54 – 1.77 (m, 4H, NCH$_2$C*H*$_{ax}$H$_{eq}$CH$_2$CH + NCH$_2$C*H*$_2$CH$_2$COH + NCH$_2$CH$_{ax}$*H*$_{eq}$CH$_2$CH), 1.87 (td, *J*=11.2, 3.6, 1H, NC*H*$_{ax}$H$_{eq}$CH$_2$CH$_2$CH), 1.94 – 2.07 (m, 2H, NCH$_2$CH$_2$CH$_{ax}$*H*$_{eq}$CH + NC*H*$_{ax}$H$_{eq}$CHCOO), 2.32 – 2.51 (m, 4H, NC*H*$_2$CH$_2$CH$_2$COH + NCH$_2$CH$_2$C*H*$_2$COH), 2.58 – 2.71 (m, 2H, NCH$_2$C*H*COO + NCH$_{ax}$*H*$_{eq}$CH$_2$CH$_2$CH), 2.82 – 2.93 (m, 1H, NCH$_{ax}$*H*$_{eq}$CHCOO), 3.79 (d, *J*=1.5, 6H, C$_{Ar}$OCH$_3$), 4.13 (q, *J*=7.1, 2H, COOC*H*$_2$CH$_3$), 6.80 – 6.89 (m, 4H, CCHC*H*COCH$_3$), 7.37 – 7.46 (m, 4H, CC*H*CHCOCH$_3$). ^{13}C NMR (101 MHz, CD$_2$Cl$_2$) δ = 14.56 (COOCH$_2$*C*H$_3$), 22.38 (NCH$_2$*C*H$_2$CH$_2$COH), 24.80 (NCH$_2$*C*H$_2$CH$_2$CH), 27.55 (NCH$_2$CH$_2$*C*H$_2$CH), 42.07 (NCH$_2$*C*HCOO), 43.28 (NCH$_2$CH$_2$*C*H$_2$COH), 58.78 (N*C*H$_2$CH$_2$CH$_2$CH), 55.58 (N*C*H$_2$CHCOO), 55.68 (d, C$_{Ar}$O*C*H$_3$), 59.88 (N*C*H$_2$CH$_2$CH$_2$COH), 60.85 (COO*C*H$_2$CH$_3$). 76.29 (*C*OH), 113.61 (d, CCH*C*HCOCH$_3$), 127.67 (d, C*C*HCHCOCH$_3$), 141.91 (d, *C*CHCHCOCH$_3$), 158.46 (d, CCHCH*C*OCH$_3$), 174.10 (COO). IR (KBr): ṽ = 3055, 2937, 2830, 1729, 1606, 1505, 1453, 1370, 1324, 1241, 1177, 1149, 1122, 1030, 851, 840, 818, 633, 591, 577 cm^{-1}. HRMS-ESI+ *m/z* [*M*+H]$^+$ calcd for $C_{26}H_{35}NO_5$: 442.2588, found: 442.2587.

***rac*-Ethyl 1-(2-{[4-(hydroxymethyl)phenyl]bis[4-methoxyphenyl]methoxy}ethyl)piperidine-3-carboxylate (12m):** ^{1}H NMR (500 MHz, CD$_2$Cl$_2$) δ = 1.20 (t, *J*=7.1, 3H, COOCH$_2$C*H*$_3$), 1.34 – 1.45 (m, 1H, NCH$_2$CH$_2$C*H*$_{ax}$H$_{eq}$), 1.47 – 1.59 (m, 1H, NCH$_2$C*H*$_{ax}$H$_{eq}$CH$_2$), 1.63 – 1.71 (m, 1H, NCH$_2$CH$_{ax}$*H*$_{eq}$CH$_2$), 1.82 – 1.91 (m, 1H, NCH$_2$CH$_2$CH$_{ax}$*H*$_{eq}$), 2.03 (td, *J*=11.1, 3.0, 1H, NC*H*$_{ax}$H$_{eq}$CH$_2$CH$_2$), 2.16 (t, *J*=10.6, 1H, NC*H*$_{ax}$H$_{eq}$CHCOO), 2.50 (tt, *J*=10.6, 3.9, 1H, NCH$_2$C*H*COO), 2.58 (td, *J*=6.0, 1.7, 2H, NC*H*$_2$CH$_2$O), 2.66 – 2.74 (m, 1H, NCH$_{ax}$*H*$_{eq}$CH$_2$CH$_2$), 2.90 – 2.98 (m, 1H, NCH$_{ax}$*H*$_{eq}$CHCOO), 3.06 – 3.19 (m, 2H, NCH$_2$C*H*$_2$O), 3.77 (s, 6H, C$_{Ar}$OCH$_3$), 4.03 – 4.09 (m, 2H, COOC*H*$_2$CH$_3$), 4.61 (s, 2H, C*H*$_2$OH), 6.78 – 6.87 (m, 4H, CCHC*H*COCH$_3$), 7.25 – 7.29 (m, 2H, CCHC*H*CCH$_2$OH), 7.30 – 7.35 (m, 4H, CC*H*CHCOCH$_3$), 7.42 – 7.45 (m, 2H, CC*H*CHCCH$_2$OH). ^{13}C NMR (126 MHz, CD$_2$Cl$_2$) δ = 14.43 (COOCH$_2$*C*H$_3$), 25.16 (NCH$_2$*C*H$_2$CH$_2$), 27.27 (NCH$_2$CH$_2$*C*H$_2$), 42.49 (NCH$_2$*C*HCOO), 54.67 (N*C*H$_2$CH$_2$CH$_2$), 55.60 (C$_{Ar}$O*C*H$_3$), 56.65 (N*C*H$_2$CHCOO), 58.87 (N*C*H$_2$CH$_2$O), 60.54 (CH$_2$COO*C*H$_2$CH$_3$), 62.14 (NCH$_2$*C*H$_2$O), 65.17 (CH$_2$OH), 86.22 (NCH$_2$CH$_2$O*C*), 113.38 (CCH*C*HCOCH$_3$), 126.77 (CCH*C*HCCH$_2$OH), 128.72 (C*C*HCHCCH$_2$OH), 130.37 (C*C*HCHCOCH$_3$), 136.87 (*C*CHCHCOCH$_3$), 140.08 (CCHCH*C*CH$_2$OH), 145.17 (*C*CHCHCCH$_2$OH), 158.94 (CCHCH*C*OCH$_3$), 174.45 (COO). IR (KBr): ṽ = 2936, 2360, 1729, 1608, 1508, 1302, 1250, 1176, 1153, 1070, 1033, 915, 827, 582 cm^{-1}. HRMS-ESI+ *m/z* [*M*+H]$^+$ calcd for $C_{32}H_{39}NO_6$: 534.2850, found: 534.2861.

***rac*- Ethyl 1-{2-[(4-{[(2-ethoxy-2-oxoethyl)amino]methyl}phenyl)bis(4-methoxyphenyl)methoxy]ethyl}piperidine-3-carboxylate (12n):** ^{1}H NMR (500 MHz, CD$_2$Cl$_2$)

δ = 1.20 (t, *J*=7.2, 3H, $CHCOOCH_2CH_3$), 1.24 (t, *J*=7.1, 3H, $CH_2COOCH_2CH_3$), 1.34 – 1.45 (m, 1H, $NCH_2CH_2H_{ax}H_{eq}$), 1.48 – 1.58 (m, 1H, $NCH_2CH_{ax}H_{eq}CH_2$), 1.67 (ddd, *J*=17.0, 7.4, 3.6, 1H, $NCH_2CH_{ax}H_{eq}CH_2$), 1.83 – 1.91 (m, 1H, $NCH_2CH_2CH_{ax}H_{eq}$), 2.03 (td, *J*=10.9, 2.4, 1H, $NCH_{ax}H_{eq}CH_2CH_2$), 2.18 (t, *J*=10.6, 1H, $NCH_{ax}H_{eq}CHCOO$), 2.51 (tt, *J*=10.5, 3.8, 1H, NCH_2CHCOO), 2.57 (t, *J*=6.0, 2H, NCH_2CH_2O), 2.66 – 2.73 (m, 1H, $NCH_{ax}H_{eq}CH_2CH_2$), 2.95 (br d, *J*=10.7, 1H, $NCH_{ax}H_{eq}CHCOO$), 3.11 (tt, *J*=9.6, 4.7, 2H, NCH_2CH_2O), 3.37 (s, 2H, CH_2NHCH_2COO), 3.75 (s, 2H, CH_2NHCH_2COO), 3.77 (s, 6H, $C_{Ar}OCH_3$), 4.03 – 4.11 (m, 2H, $CHCOOCH_2CH_3$), 4.14 (q, *J*=7.2, 2H, $CH_2COOCH_2CH_3$), 6.79 – 6.86 (m, 4H, $CCHCHCOCH_3$), 7.22 – 7.27 (m, 2H, $CCHCHCCH_2NH$), 7.30 – 7.35 (m, 4H, $CCHCHCOCH_3$), 7.37 – 7.41 (m, 2H, $CCHCHCCH_2NH$). ^{13}C NMR (126 MHz, CD_2Cl_2) δ = 14.44 (d, $CHCOOCH_2CH_3$ + $CHCOOCH_2CH_3$), 25.16 ($NCH_2CH_2CH_2$), 27.27 ($NCH_2CH_2CH_2$), 42.49 (NCH_2CHCOO), 50.66 (CH_2NHCH_2COO), 53.19 (CH_2NHCH_2COO), 54.67 ($NCH_2CH_2CH_2$), 55.59 ($C_{Ar}OCH_3$), 56.71 (NCH_2CHCOO), 58.89 (NCH_2CH_2O), 60.52 ($CHCOOCH_2CH_3$), 60.98 ($CH_2COOCH_2CH_3$), 62.17 (NCH_2CH_2O), 86.21 (NCH_2CH_2OC), 113.35 ($CCHCHCOCH_3$), 127.95 ($CCHCHCCH_2NH$), 128.62 ($CCHCHCCH_2NH$), 130.36 ($CCHCHCOCH_3$), 137.00 ($CCHCHCOCH_3$), 138.85 ($CCHCHCCH_2NH$), 144.53 ($CCHCHCCH_2NH$), 158.91 ($CCHCHCOCH_3$), 172.76 ($NHCH_2COO$), 174.43 (NCH_2CHCOO). IR (KBr): ṽ = 2948, 2835, 1731, 1608, 1582, 1509, 1464, 1440, 1367, 1302, 1250, 1176, 1071, 1034, 916, 828, 583 cm^{-1}. HRMS-ESI+ *m/z* $[M+H]^+$ calcd for $C_{36}H_{46}N_2O_7$: 619.3378, found: 619.3394.

***rac*-Ethyl 1-{2-[(4-{[(3-methoxy-3-oxopropyl)amino]methyl}phenyl)bis(4-methoxyphenyl)methoxy]ethyl}piperidine-3-carboxylate (12o):** ^1H NMR (500 MHz, CD_2Cl_2) δ = 1.20 (t, *J*=7.1, 3H, $COOCH_2CH_3$), 1.33 – 1.45 (m, 1H, $NCH_2CH_2CH_{ax}H_{eq}$), 1.48 – 1.59 (m, 1H, $NCH_2CH_{ax}H_{eq}CH_2$), 1.63 – 1.71 (m, 1H, $NCH_2CH_{ax}H_{eq}CH_2$), 1.83 – 1.91 (m, 1H, $NCH_2CH_2CH_{ax}H_{eq}$), 2.00 – 2.06 (m, 1H, $NCH_{ax}H_{eq}CH_2CH_2$), 2.17 (t, *J*=10.6, 1H, $NCH_{ax}H_{eq}CHCOO$), 2.46 – 2.54 (m, 3H, NCH_2CHCOO + $NHCH_2CH_2COO$), 2.57 (t, *J*=5.9, 2H, NCH_2CH_2O), 2.66 – 2.73 (m, 1H, $NCH_{ax}H_{eq}CH_2CH_2$), 2.87 (t, *J*=6.5, 2H, $NHCH_2CH_2COO$), 2.92 – 2.98 (m, 1H, $NCH_{ax}H_{eq}CHCOO$), 3.06 – 3.18 (m, 2H, NCH_2CH_2O), 3.64 (s, 3H, $COOCH_3$), 3.74 (s, 2H, $C_{Ar}CH_2NH$), 3.77 (s, 6H, $C_{Ar}OCH_3$), 4.04 – 4.10 (m, 2H, $CH_2COOCH_2CH_3$), 6.79 – 6.84 (m, 4H, $CCHCHCOCH_3$), 7.21 – 7.25 (m, 2H, $CCHCHCCH_2NH$), 7.30 – 7.35 (m, 4H, $CCHCHCOCH_3$), 7.36 – 7.40 (m, 2H, $CCHCHCCH_2NH$). ^{13}C NMR (126 MHz, CD_2Cl_2) δ = 14.43 ($COOCH_2CH_3$), 25.16 ($NCH_2CH_2CH_2$), 27.27 ($NCH_2CH_2CH_2$), 35.08 ($NHCH_2CH_2COO$) 42.49 (NCH_2CHCOO), 45.10 ($NHCH_2CH_2COO$), 51.78 ($COOCH_3$), 53.62 ($C_{Ar}CH_2NH$), 54.66 ($NCH_2CH_2CH_2$), 55.59 ($C_{Ar}OCH_3$), 56.70 (NCH_2CHCOO), 58.89 (NCH_2CH_2O), 60.52 ($CHCOOCH_2CH_3$), 62.15 (NCH_2CH_2O), 86.21 (NCH_2CH_2OC), 113.34 ($CCHCHCOCH_3$), 127.77 ($CCHCHCCH_2NH$), 128.59 ($CCHCHCCH_2NH$), 130.35 ($CCHCHCOCH_3$), 137.04 ($CCHCHCOCH_3$), 139.46 ($CCHCHCCH_2NH$), 144.30 ($CCHCHCCH_2NH$), 158.90 ($CCHCHCOCH_3$), 173.46 ($NH_2CH_2CH_2COO$), 174.43 (NCH_2CHCOO). IR (KBr): ṽ = 2936, 1607, 1560, 1508, 1406, 1302, 1250, 1176, 1068, 1035, 826, 583 cm^{-1}. HRMS-ESI+ *m/z* $[M+H]^+$ calcd for $C_{36}H_{46}N_2O_7$: 619.3378, found: 619.3381.

***rac*-Ethyl 1-(2-{[4-({[1-(ethoxycarbonyl)cyclopropyl]amino}methyl)phenyl]bis[4-methoxyphenyl]methoxy}ethyl)piperidine-3-carboxylate (12p):** ^1H NMR (400 MHz, CD_2Cl_2) δ = 0.98 (q, *J*=3.9, 2H, $NHCCH_{ax}H_{eq}$), 1.17 – 1.28 (m, 8H, $CHCOOCH_2CH_3$ + $CCOOCH_2CH_3$ + $NHCCH_{ax}H_{eq}$), 1.33 – 1.45 (m, 1H, $NCH_2CH_2CH_{ax}H_{eq}$), 1.46 – 1.62 (m, 1H, $NCH_2CH_{ax}H_{eq}CH_2$), 1.67 (dt, *J*=12.5, 3.7, 1H, $NCH_2CH_{ax}H_{eq}CH_2$), 1.82 – 1.92 (m, 1H, $NCH_2CH_2CH_{ax}H_{eq}$), 2.03 (td, *J*=10.9, 2.9, 1H, $NCH_{ax}H_{eq}CH_2CH_2$), 2.17 (t, *J*=10.6, 1H, $NCH_{ax}H_{eq}CHCOO$), 2.50 (tt, *J*=10.4, 3.8, 1H, NCH_2CHCOO), 2.57 (t, *J*=6.0, 2H, NCH_2CH_2O), 2.65 – 2.74 (m, 1H, $NCH_{ax}H_{eq}CH_2CH_2$), 2.91 – 2.99 (m, 1H, $NCH_{ax}H_{eq}CHCOO$), 3.11 (tt, *J*=5.8, 3.1, 2H, NCH_2CH_2O), 3.77 (s, 6H, $C_{Ar}OCH_3$), 3.82 (s, 2H, $C_{Ar}CH_2NH$), 4.03 – 4.14 (m, 4H, $CHCOOCH_2CH_3$ + $CCOOCH_2CH_3$), 6.78 – 6.85 (m, 4H,

CCHC*H*COCH$_3$), 7.18 – 7.26 (m, 2H, CCHC*H*CCH$_2$NH), 7.30 – 7.34 (m, 4H, CC*H*CHCOCH$_3$), 7.35 – 7.39 (m, 2H, CC*H*CHCCH$_2$NH). ^{13}C NMR (101 MHz, CD$_2$Cl$_2$) δ = 14.47 (d, NHC*C*H$_2$), 17.79 (CHCOOCH$_2$*C*H$_3$ + CCOOCH$_2$*C*H$_3$), 25.17 (NCH$_2$*C*H$_2$CH$_2$), 27.28 (NCH$_2$CH$_2$*C*H$_2$), 41.38 (NH*C*), 42.50 (NCH$_2$*C*HCOO), 51.74 (C$_{Ar}$*C*H$_2$NH), 54.67 (N*C*H$_2$CH$_2$CH$_2$), 55.59 (C$_{Ar}$O*C*H$_3$), 56.71 (N*C*H$_2$CHCOO), 58.89 (N*C*H$_2$CH$_2$O), 60.51 (CHCOO*C*H$_2$CH$_3$), 61.10 (CCOO*C*H$_2$CH$_3$), 62.17 (NCH$_2$*C*H$_2$O), 86.21 (NCH$_2$CH$_2$O*C*), 113.34 (CCH*C*HCOCH$_3$), 127.97 (CCH*C*HCCH$_2$NH), 128.55 (C*C*HCHCCH$_2$NH), 130.34 (C*C*HCHCOCH$_3$), 137.04 (*C*CHCHCOCH$_3$), 139.67 (CCHCH*C*CH$_2$NH), 144.31 (*C*CHCHCCH$_2$NH), 158.90 (CCHCH*C*OCH$_3$), 174.43 (CH*C*OO), 175.45 (C*C*OO). IR (KBr): ṽ = 2938, 1724, 1608, 1508, 1464, 1368, 1301, 1250, 1176, 1152, 1091, 1071, 1033, 969, 916, 827, 751, 733, 582 cm^{-1}. HRMS-ESI+ *m/z* [*M*+H]$^+$ calcd for $C_{38}H_{48}N_2O_7$: 645.3534, found: 645.3535.

***rac*-Ethyl 1-{2-[(4-{[(1-ethoxy-2-methyl-1-oxopropan-2-yl)amino]methyl}phenyl)bis(4-methoxyphenyl)methoxy]ethyl}piperidine-3-carboxylate (12q):** ^{1}H NMR (400 MHz, CD$_2$Cl$_2$) δ = 1.20 (t, *J*=7.1, 3H, CHCOOCH$_2$C*H$_3$*), 1.26 (t, *J*=7.1, 3H, CCOOCH$_2$C*H$_3$*), 1.31 (s, 6H, CC*H$_3$*), 1.34 – 1.44 (m, 1H, NCH$_2$CH$_2$C*H$_{ax}$*H$_{eq}$), 1.47 – 1.59 (m, 1H, NCH$_2$C*H$_{ax}$*H$_{eq}$CH$_2$), 1.63 – 1.72 (m, 1H, NCH$_2$CH$_{ax}$*H$_{eq}$*CH$_2$), 1.83 – 1.92 (m, 1H, NCH$_2$CH$_2$CH$_{ax}$*H$_{eq}$*), 2.03 (td, *J*=11.0, 3.0, 1H, NC*H$_{ax}$*H$_{eq}$CH$_2$CH$_2$), 2.18 (t, *J*=10.6, 1H, NC*H$_{ax}$*H$_{eq}$CHCOO), 2.50 (tt, *J*=10.4, 3.8, 1H, NCH$_2$C*H*COO), 2.58 (t, *J*=6.0, 2H, NC*H$_2$*CH$_2$O), 2.65 – 2.75 (m, 1H, NCH$_{ax}$*H$_{eq}$*CH$_2$CH$_2$), 2.91 – 2.99 (m, 1H, NCH$_{ax}$*H$_{eq}$*CHCOO), 3.11 (tt, *J*=6.4, 3.3, 2H, NCH$_2$C*H$_2$*O), 3.58 (s, 2H, C$_{Ar}$C*H$_2$*NH), 3.77 (s, 6H, C$_{Ar}$OCH$_3$), 4.04 – 4.10 (m, 2H, CHCOOC*H$_2$*CH$_3$), 4.14 (q, *J*=7.1, 2H, CCOOC*H$_2$*CH$_3$), 6.78 – 6.85 (m, 4H, CCHC*H*COCH$_3$), 7.22 – 7.27 (m, 2H, CCHC*H*CCH$_2$NH), 7.29 – 7.35 (m, 4H, CC*H*CHCOCH$_3$), 7.36 – 7.40 (m, 2H, CC*H*CHCCH$_2$NH). ^{13}C NMR (101 MHz, CD$_2$Cl$_2$) δ = 14.44 (CHCOOCH$_2$*C*H$_3$), 14.55 (CCOOCH$_2$*C*H$_3$), 25.17 (NCH$_2$*C*H$_2$CH$_2$), 25.58 (C*C*H$_3$), 27.28 (NCH$_2$CH$_2$*C*H$_2$), 42.50 (NCH$_2$*C*HCOO), 48.76 (C$_{Ar}$*C*H$_2$NH), 54.67 (N*C*H$_2$CH$_2$CH$_2$), 55.59 (C$_{Ar}$O*C*H$_3$), 56.71 (N*C*H$_2$CHCOO), 58.88 (N*C*H$_2$CH$_2$O), 59.43 (NH*C*CH$_3$), 60.51 (CHCOO*C*H$_2$CH$_3$), 61.02 (CCOO*C*H$_2$CH$_3$), 62.18 (NCH$_2$*C*H$_2$O), 86.21 (NCH$_2$CH$_2$O*C*), 113.35 (CCH*C*HCOCH$_3$), 128.05 (CCH*C*HCCH$_2$NH), 128.61 (C*C*HCHCCH$_2$NH), 130.36 (C*C*HCHCOCH$_3$), 137.02 (*C*CHCHCOCH$_3$), 139.65 (CCHCH*C*CH$_2$NH), 144.42 (*C*CHCHCCH$_2$NH), 158.91 (CCHCH*C*OCH$_3$), 174.43 (CH*C*OO), 177.14 (C*C*OO). IR (KBr): ṽ = 2979, 2937, 1729, 1608, 1582, 1509, 1464, 1302, 1250, 1176, 1140, 1071, 1033, 915, 826, 660, 582 cm^{-1}. HRMS-ESI+ *m/z* [*M*+H]$^+$ calcd for $C_{38}H_{50}N_2O_7$: 647.3691, found: 647.3688.

***rac*-4-{[4-({2-[3-(Ethoxycarbonyl)piperidin-1-yl]ethoxy}bis{4-methoxyphenyl}methyl)benzyl]amino}butanoic acid (12s):** ^{1}H NMR (400 MHz, MeOD) δ = 1.20 (t, *J*=7.1, 3H, COOCH$_2$C*H$_3$*), 1.36 – 1.47 (m, 1H, NCH$_2$CH$_2$C*H$_{ax}$*H$_{eq}$), 1.48 – 1.64 (m, 1H, NCH$_2$C*H$_{ax}$*H$_{eq}$CH$_2$), 1.70 (dt, *J*=13.3, 3.7, 1H, NCH$_2$CH$_{ax}$*H$_{eq}$*CH$_2$), 1.79 – 1.94 (m, 3H, NCH$_2$CH$_2$CH$_{ax}$*H$_{eq}$* + NHCH$_2$C*H$_2$*CH$_2$COOH), 2.08 (td, *J*=11.2, 3.0, 1H, NC*H$_{ax}$*H$_{eq}$CH$_2$CH$_2$), 2.17 – 2.31 (m, 3H, NC*H$_{ax}$*H$_{eq}$CHCOO + NHCH$_2$CH$_2$C*H$_2$*COOH), 2.52 (ddt, *J*=10.6, 7.6, 3.8, 1H, NCH$_2$C*H*COO), 2.64 (td, *J*=5.7, 3.0, 2H, NC*H$_2$*CH$_2$O), 2.70 – 2.78 (m, 1H, NCH$_{ax}$*H$_{eq}$*CH$_2$CH$_2$), 2.81 (t, *J*=6.9, 2H, NHC*H$_2$*CH$_2$CH$_2$COOH), 2.99 – 3.08 (m, 1H, NCH$_{ax}$*H$_{eq}$*CHCOO), 3.23 (qt, *J*=9.9, 5.7, 2H, NCH$_2$C*H$_2$*O), 3.77 (s, 6H, C$_{Ar}$OCH$_3$), 3.88 (s, 2H, C$_{Ar}$C*H$_2$*NH), 4.06 – 4.12 (m, 2H, CH$_2$COOC*H$_2$*CH$_3$), 6.82 – 6.88 (m, 4H, CCHC*H*COCH$_3$), 7.28 – 7.35 (m, 6H, CC*H*CHCOCH$_3$ + CCHC*H*CCH$_2$NH), 7.44 – 7.49 (m, 2H, CC*H*CHCCH$_2$NH). ^{13}C NMR (126 MHz, Methanol-*d$_4$*) δ = 14.52 (COOCH$_2$*C*H$_3$), 25.25 (NCH$_2$*C*H$_2$CH$_2$), 25.61 (NHCH$_2$*C*H$_2$CH$_2$COOH), 27.68 (NCH$_2$CH$_2$*C*H$_2$), 37.06 (NHCH$_2$CH$_2$*C*H$_2$COOH), 42.82 (NCH$_2$*C*HCOO), 49.80 (NH*C*H$_2$CH$_2$CH$_2$COOH), 53.00 (C$_{Ar}$*C*H$_2$NH), 55.19 (N*C*H$_2$CH$_2$CH$_2$), 55.71 (C$_{Ar}$O*C*H$_3$), 57.05 (N*C*H$_2$CHCOO), 59.34 (N*C*H$_2$CH$_2$O), 61.57 (CHCOO*C*H$_2$CH$_3$), 62.51 (NCH$_2$*C*H$_2$O), 87.51 (NCH$_2$CH$_2$O*C*), 114.09 (CCH*C*HCOCH$_3$), 129.49 (d, CCH*C*HCCH$_2$NH + C*C*HCHCCH$_2$NH), 131.30 (C*C*HCHCOCH$_3$), 135.94 (CCHCH*C*CH$_2$NH),

137.17 (*C*CHCHCOCH$_3$), 146.80 (*C*CHCHCCH$_2$NH), 160.19 (CCHCH*C*OCH$_3$), 175.36 (*C*OOCH$_2$CH$_3$), 181.74 (CH$_2$*C*OOH). IR (KBr): ṽ = 2939, 1730, 1609, 1578, 1508, 1465, 1404, 1302, 1250, 1176, 1153, 1070, 1033, 916, 828, 582 cm^{-1}. HRMS-ESI+ *m/z* [*M*+H]$^+$ calcd for $C_{36}H_{46}N_2O_7$: 619.3378, found: 619.3382.

***rac*-4-{[4-({2-[3-(Ethoxycarbonyl)piperidin-1-yl]ethoxy}bis{4-methoxyphenyl}methyl)benzyl]amino}benzoic acid (12t):** ^{1}H NMR (400 MHz, CD$_2$Cl$_2$) δ = 1.20 (t, *J*=7.1, 3H, COOCH$_2$C*H$_3$*), 1.35 – 1.47 (m, 1H, NCH$_2$CH$_2$C*H$_{ax}$*H$_{eq}$), 1.56 – 1.73 (m, 2H, NCH$_2$C*H$_{ax}$*H$_{eq}$CH$_2$ + NCH$_2$CH$_{ax}$*H$_{eq}$*CH$_2$), 1.89 – 1.99 (m, 1H, NCH$_2$CH$_2$CH$_{ax}$*H$_{eq}$*), 2.08 (td, *J*=10.9, 4.2, 1H, NC*H$_{ax}$*H$_{eq}$CH$_2$CH$_2$), 2.20 (t, *J*=11.0, 1H, NC*H$_{ax}$*H$_{eq}$CHCOO), 2.60 – 2.76 (m, 3H, NCH$_2$C*H*COO + NC*H$_2$*CH$_2$O), 2.90 (br d, *J*=11.5, 1H, NCH$_{ax}$*H$_{eq}$*CH$_2$CH$_2$), 3.09 – 3.17 (m, 1H, NCH$_{ax}$*H$_{eq}$*CHCOO), 3.21 (t, *J*=6.0, 2H, NCH$_2$C*H$_2$*O), 3.76 (s, 6H, C$_{Ar}$OCH$_3$), 4.04 – 4.10 (m, 2H, COOC*H$_2$*CH$_3$), 4.22 (s, 2H, C$_{Ar}$C*H$_2$*NH), 6.54 – 6.65 (m, 2H, CC*H*CHCCOOH), 6.77 – 6.85 (m, 4H, CCHC*H*COCH$_3$), 7.15 – 7.23 (m, 2H, CCHC*H*CCH$_2$NH), 7.27 – 7.36 (m, 4H, CC*H*CHCOCH$_3$), 7.37 – 7.45 (m, 2H, CC*H*CHCCH$_2$NH), 7.77 – 7.87 (m, 2H, CCHC*H*CCOOH). ^{13}C NMR (101 MHz, CD$_2$Cl$_2$) δ = 14.55 (COOCH$_2$*C*H$_3$), 24.56 (NCH$_2$*C*H$_2$CH$_2$), 27.32 (NCH$_2$CH$_2$*C*H$_2$), 41.75 (NCH$_2$*C*HCOO), 47.91 (C$_{Ar}$*C*H$_2$NH), 54.33 (N*C*H$_2$CH$_2$CH$_2$), 55.74 (C$_{Ar}$O*C*H$_3$), 56.02 (N*C*H$_2$CHCOO), 58.40 (N*C*H$_2$CH$_2$O), 60.86 (COO*C*H$_2$CH$_3$), 61.42 (NCH$_2$*C*H$_2$O), 86.57 (NCH$_2$CH$_2$O*C*), 112.02 (C*C*HCHCCOOH), 113.58 (CCH*C*HCOCH$_3$), 119.69 (CCHCH*C*COO), 127.71 (CCH*C*HCCH$_2$NH), 129.16 (C*C*HCHCCH$_2$NH), 130.45 (d, *J*=2.9, C*C*HCHCOCH$_3$), 132.39 (CCH*C*HCCOOH), 136.88 (d, *J*=4.5, *C*CHCHCOCH$_3$), 137.35 (CCHCH*C*CH$_2$NH), 144.91 (*C*CHCHCCH$_2$NH), 152.58 (*C*CHCHCCOO), 159.12 (CCHCH*C*OCH$_3$), 170.67 (C$_{Ar}$*C*OO), 174.24 (CH*C*OO). IR (KBr): ṽ = 2935, 2836, 1728, 1606, 1508, 1464, 1413, 1372, 1303, 1251, 1175, 1113, 1072, 1033, 918, 827, 776, 701, 583 cm^{-1}. HRMS-ESI- *m/z* [*M*-H]$^-$ calcd for $C_{39}H_{44}N_2O_7$: 651.3076, found: 651.3078.
